# Stress Granule-Defective Mutants Deregulate Stress Responsive Transcripts

**DOI:** 10.1371/journal.pgen.1004763

**Published:** 2014-11-06

**Authors:** Xiaoxue Yang, Yi Shen, Elena Garre, Xinxin Hao, Daniel Krumlinde, Marija Cvijović, Christina Arens, Thomas Nyström, Beidong Liu, Per Sunnerhagen

**Affiliations:** 1 School of Life Science and Engineering, Harbin Institute of Technology, Harbin, China; 2 Department of Chemistry and Molecular Biology, Lundberg Laboratory, University of Gothenburg, Göteborg, Sweden; 3 Department of Mathematical Sciences, Chalmers University of Technology, Göteborg, Sweden; 4 Department of Mathematical Sciences, University of Gothenburg, Göteborg, Sweden; Brigham and Women's Hospital Harvard School of Medicine, United States of America

## Abstract

To reduce expression of gene products not required under stress conditions, eukaryotic cells form large and complex cytoplasmic aggregates of RNA and proteins (stress granules; SGs), where transcripts are kept translationally inert. The overall composition of SGs, as well as their assembly requirements and regulation through stress-activated signaling pathways remain largely unknown.

We have performed a genome-wide screen of *S. cerevisiae* gene deletion mutants for defects in SG formation upon glucose starvation stress. The screen revealed numerous genes not previously implicated in SG formation. Most mutants with strong phenotypes are equally SG defective when challenged with other stresses, but a considerable fraction is stress-specific. Proteins associated with SG defects are enriched in low-complexity regions, indicating that multiple weak macromolecule interactions are responsible for the structural integrity of SGs. Certain SG-defective mutants, but not all, display an enhanced heat-induced mutation rate. We found several mutations affecting the Ran GTPase, regulating nucleocytoplasmic transport of RNA and proteins, to confer SG defects. Unexpectedly, we found stress-regulated transcripts to reach more extreme levels in mutants unable to form SGs: stress-induced mRNAs accumulate to higher levels than in the wild-type, whereas stress-repressed mRNAs are reduced further in such mutants.

Our findings are consistent with the view that, not only are SGs being regulated by stress signaling pathways, but SGs also modulate the extent of stress responses. We speculate that nucleocytoplasmic shuttling of RNA-binding proteins is required for gene expression regulation during stress, and that SGs modulate this traffic. The absence of SGs thus leads the cell to excessive, and potentially deleterious, reactions to stress.

## Author Summary

When cells encounter harsh conditions, they face an energy crisis since the stress will reduce their energy production, and at the same time cause extra demands on energy expenditure. To tackle this dilemma, cells under stress form giant agglomerates of RNA and protein, called stress granules. In these, mRNA molecules are kept silent, preventing waste of energy on producing proteins not needed under these conditions. A few mRNAs, encoding proteins required for the cell to survive, stay outside of stress granules and escape this silencing. This mechanism can protect plants and microbes against cold spells or heat shocks, and human cells exposed to oxidative damage or toxic drugs. We have investigated which genes are necessary to form stress granules, and their impact on the stress response. We discovered that mutant cells unable to form stress granules overreacted to stress, in that they produced much higher levels of the induced mRNAs. We think this means that gene regulatory proteins are sequestered inside stress granules, inhibiting their action. Stress granules may thus function as moderators that dampen the stress response, safeguarding the cell against excessive reactions.

## Introduction

Eukaryotic cells exposed to physical, chemical, or nutritional stress, activate a variety of responses. For short-term survival, rapid events mainly regulated at the post-translational level, such as phosphorylation, protein binding and relocalization, dominate. For optimization of medium-term survival and recovery, the expression program needs to change rapidly towards proteins needed for survival during the stress phase, at the expense of products linked to proliferation. Although transcriptional initiation is a major regulatory level, in particular during chronic stress, a substantial fraction of the expression change occurs on the post-transcriptional level. This is because protein synthesis consumes a major fraction of the cell's total energy production. Hence, translation needs to be tightly regulated under stress conditions, which invariably confer an energy drain on the cell. Consequently, following an increase in stress level, *S. cerevisiae* cells decrease the stability [1]–[3] and translation rate [4]–[6] of most mRNAs. Under such conditions, transcripts encoding proteins needed for stress survival are instead stabilized and their translation enhanced. This transition can be mediated through a shift in translation initiation factors [7]. As part of the post-transcriptional stress response program, eukaryotic cells form two types of large cytoplasmic granules containing RNA and proteins. Processing bodies (PBs) are present constitutively, but increase in number upon stress. They contain components of the mRNA degradation and silencing machineries. Stress granules (SGs) only form under conditions of severe stress. They are considered to consist of stalled translation initiation complexes, and contain initiation factors and 40S subunit components, as well as other mRNAs binding proteins; however 40S components are absent from budding yeast SGs formed after glucose deprivation [8]. Although certain proteins are found in PBs but not in SGs and *vice versa*, there is also a number of proteins common to PBs and SGs [9], [10], indicating the possibility of dynamic exchange of material between the two types of granules. Mutants deficient in PB formation most often also have SG deficiencies, whereas the opposite does not hold: a mutant deficient in SG formation does not necessarily have reduced PB numbers [11]. Upon stress, the number of PBs increases before the appearance of SGs; the latter are frequently observed physically close to or overlapping with PBs. These observations have led to a model where the formation of SGs is contingent on the prior formation of PBs in their immediate vicinity [11]. However, this model has recently been challenged. In yeast, SGs have been observed to be regulated by different signaling pathways than PBs [12], and individual SGs can be observed microscopically to arise independently of pre-existing PBs [13]. In mammalian cells, knockdown of specific mRNAs suppressed formation of PBs but not SGs [14].

More recently, several indications have appeared that SGs not only sequester transcripts and translation components, but also signaling and catalytic proteins. Members of the yeast TORC1 signaling complex, Tor1 (mTOR in mammalian cells) and Kog1 (Raptor), are found in SGs after heat stress [15]. In mammalian cells, SGs sequester RACK, a MAPKKK modulator under certain stress conditions, suppressing activation of p38 signaling that would otherwise promote apoptosis [16]. The G3BP1 (*S. cerevisiae* Bre5) and USP10 (*S. cerevisiae* Ubp3) proteins are both SG components. Under normal conditions, G3BP1 binds to and inhibits USP10, but in stress, when both proteins become incorporated into SGs, their interaction changes and this unleashes the activity of USP10 [17]. The homologous proteins in fission yeast, Nxt3 and Ubp3, are also SG components [18], providing an example of evolutionary conservation of function. A possible connection between SGs and neurological disorders has attracted interest, as several neuronal proteins with roles in RNA binding and/or protein aggregation have been localized to SGs [19]. For instance, FRMP, a translational regulator implicated in fragile X syndrome, localizes to SGs under stress [20]. These findings have extended the view of SGs from being sites for mRNA storage, sorting, silencing, and reactivation, into centers for modulation of a variety of cellular stress responses.

There are several notable species-specific differences in how signaling pathways affect SG formation. Phosphorylation of the translation initiation factor eIF2α is both necessary and sufficient in mammalian cells [21], whereas eIF2α phosphorylation is not required for SG formation after heat shock in budding yeast [22], nor under any condition tested in fission yeast [23], [24]. Also, mounting evidence indicates that the signaling pathways needed for SG formation are stress-specific. In fission yeast, protein kinase A (PKA) is required for SG formation after glucose starvation, but not after hyperosmotic shock [24]. In *S. cerevisiae*, different stress conditions lead to formation of SGs of different composition, and with different assembly requirements. Thus, SGs can form after azide treatment independently of PB formation ability or of the mRNA-binding proteins Pbp1 (Ataxin-2 homolog) and Pub1 (TIA-1 homolog), but not after glucose deprivation [25]. Further evidence for a determined assembly order of SGs comes from the observation that in budding yeast cells upon mild heat shock, translation factors form foci at sites where SGs will later form when the temperature is raised further [26].

Previously, only few studies of the global requirements for formation and disassembly of SGs in mammalian cells and in yeast have been performed. An RNAi screen for genes necessary for normal SG formation in human cultured cells yielded an overrepresentation of functions related to O-linked glycosylation [14]. Recently, a global screen in *S. cerevisiae* for mutants defective in SG disassembly identified autophagy involving the yeast homolog of valosin-containing protein (VCP) as the pathway for clearance of SGs from the cell [27].

In this work, we have screened a genomic library of haploid deletion mutants, representing all non-essential genes of *S. cerevisiae*, for their ability to form SGs under nutrient stress induced by 2-deoxyglucose (2-DG). Mutants identified in the primary screen as deficient or hyperproficient in SG formation were also tested for SG formation under other stress agents. Most of these mutants display a general defect in SG formation, whereas the defect in others is specific for certain stress types. The protein products of the genes that we identify as required for SG formation are enriched for low-complexity regions (LCRs). SG defects are associated with enhanced mutability upon heat stress. We find functions related to the Ran^GTP^/Ran^GDP^ system, driving nucleocytoplasmic transport, to be necessary for regulated formation of SG under stress. Importantly, we observe here that mutants unable to form SGs are also deficient in their ability to modulate changes gene expression associated with the stress response; stress-activated transcripts are hyperinduced, whereas those down-regulated by stress are repressed even further than in the wild-type. This strongly suggests that not only is SG formation regulated by stress-activated signaling pathways, but SGs actively moderate the stress response at the transcript level.

## Results

### Genome-wide high-content gene deletion mutant screen for deficient SG formation

**Figure 1:**
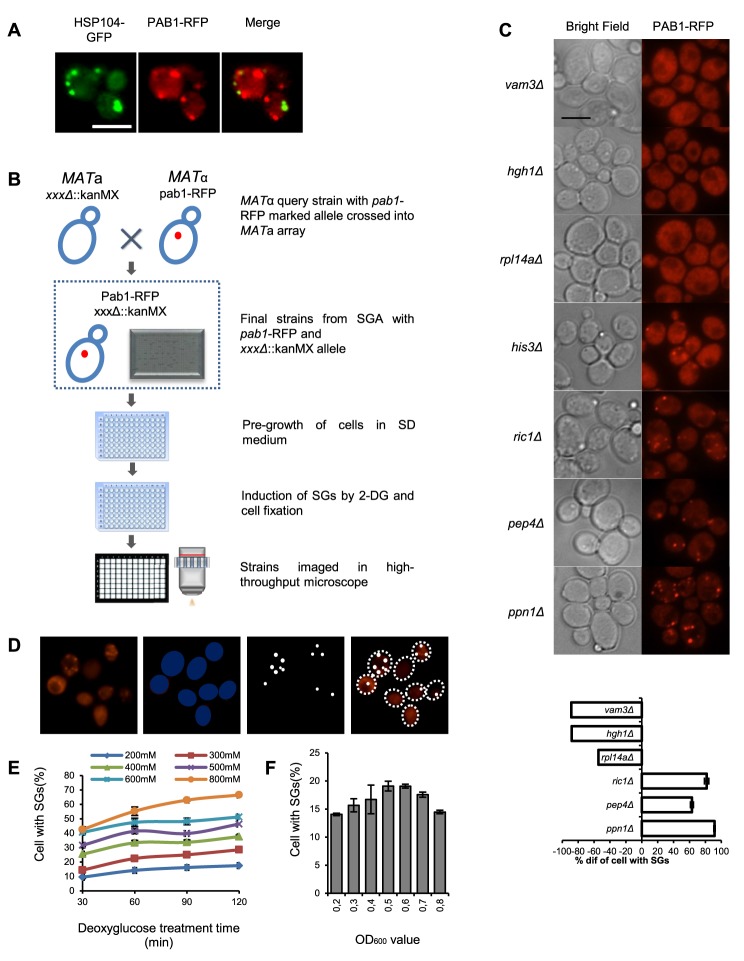
Workflow of genome-wide screen for SG-defective mutants. **A**) Stress granules induced by 400 mM 2-DG as visualized by Pab1-RFP do not co-localize with Hsp104-GFP granules. Scale bar, 5 µm. **B**) Construction of haploid strain set containing gene deletions and Pab1-RFP, transfer to microtiter plates for storage, pre-growth of cells, induction of SGs by 2-DG, fixation and visualization. **C**) Upper panel: Micrographs of mutants displaying fewer SGs than wt (*vam3Δ*, *hgh1Δ*, *rpl14aΔ*); wt level (*his3Δ*); or more SGs than wt (*ric1Δ*, *pep4Δ*, *ppn1Δ*). Left column, bright field; right column, RFP fluorescence. Scale bar, 5 µm. Lower panel: Values are normalized such that the fraction of Pab1 granule-positive cells in wt cells for the stress condition in question is set to 0. A value of −100% means that there are no cells of that mutant with SGs; a value of −50% means that the fraction of SG-positive cells in that mutant is half of the fraction in the wt under the same conditions; a value of +100% means that the fraction is twice that in the wt. **D**) Identification and quantification of SGs by automated image analysis. **E**) Fraction of SG-positive cells as a function of 2-DG concentration. Cells were grown to A_600 nm_ = 0.5 and SGs were recorded at the indicated times of 2-DG exposure. **F**) Fraction of SG-positive cells as a function of culture density. Cells were exposed to 400 mM 2-DG concentration for 30 min.

To be able to perform a genome-wide screen, we defined the stress conditions under which to perform it, applying specific criteria. First, we wanted a robust induction of SGs. Second, for the purposes of a high-throughput genome-wide screen, with robotic handling in microtiter plate format, stress induction needed to be quick and synchronized between many samples. This consideration ruled out any scheme that included centrifugation and aspiration of medium during the induction procedure. Third, in order to minimize a potential influence from proteins and pathways regulating formation of cytoplasmic aggregates of damaged proteins, we wanted conditions where there was a clear physical separation between SGs and such other protein foci. To this end, we used RFP-labeled Pab1 (poly A-binding protein) as a marker for SGs, and GFP-labeled Hsp104 as a marker for aggregates of damaged proteins (Fig. 1 A). We evaluated treatments established to induce SG formation in *S, cerevisiae* or other organisms, including glucose deprivation, arsenite, hyperosmotic shock by elevated Na^+^ or K^+^ concentration, heat, oxidative stress through H_2_O_2_, ethanol, stationary phase, and combinations of these (Fig. S1). The most reproducible and stable appearance of SGs was found to occur after glucose deprivation, or after a combination of 8% ethanol and heat shock. However, only after glucose deprivation was a clear physical separation observed between Pab1-RFP and Hsp104-GFP foci (Fig. 1 A and Fig. S1). We selected glucose starvation as the stress agent, but needed to modify the procedure further to eliminate washing and centrifugation steps. Therefore, we substituted removal of glucose for addition of the glucose competitor, 2-DG, to a final concentration of 400 mM, into the medium. This treatment was found to induce SGs in about 35% of wt cells, and this number was relatively stable over a period of 60 min. (Fig. 1 E).

For the screening purposes, we constructed a genome-wide set of *S. cerevisiae* haploid genomic deletion mutant strains expressing Pab1 carboxy-terminally tagged with RFP from the *PAB1* chromosomal locus in the BY4741 background as described in Materials and Methods (Fig. 1 B). All resultant strains were screened, as described in Materials and Methods, for the fraction of cells foci positive for Pab1-RFP after 2-DG exposure, using automated image analysis (Fig. 1 D,E). The result was quantitated as the deviation from the level in wt cells, which was set to ±0% (Fig. 1 C).

We selected 194 mutants (Table S1) which fulfilled the following two criteria: a) the absolute value of the SG phenotype in 2-DG, decreased or increased, exceeded 20%; b) the difference to the level in wt cells under the same conditions was statistically significant (P<0.05). Out of this set, a total of 73 null mutants (Table S2 A) with strongly decreased SG phenotypes were verified by manual microscopy. The confirmation rate (a statistically decreased SG phenotype was also observed by visual inspection) was high, with increasing rates up to 100% for the most extreme phenotypes (Table 1). This verifies that the image analysis algorithm efficiently and selectively identifies SGs. Our screening approach could potentially score mutants expressing lower levels of Pab1 as SG negative. To test how much this was a concern, we measured Pab1 levels in nine mutants identified as SG-defective by western blotting. All these mutants (except one, *arc1Δ*) have normal Pab1 levels, showing that deficient Pab1 expression was not the explanation why these mutants scored as SG-defective (Fig. S2). We further used another established SG marker, Pbp1, to verify that the phenotypes scored for Pab1-positive SGs also applied to other SG components. For the tested mutants, the relative changes in Pbp1-positive SGs upon glucose starvation largely paralleled those for Pab1-positive SGs (Fig. S3), with the exception of *top3*Δ.

**Table 1:** Rates of manual confirmation of large-scale screening hits with decreased SG numbers.

SG phenotype difference from wt (%)	No. mutants	No. manually confirmed	Confirmation rate (%)
−90–−100	8	8	100
−80–−90	7	7	100
−70–−80	5	4	80
−60–−70	5	4	80
−50–−60	17	12	71
−40–−50	18	15	83
−30–−40	21	14	67

Numbers refer to SGs observed under standard screening conditions (exposure to 400 mM 2-DG for 90 min).

**Figure 2:**
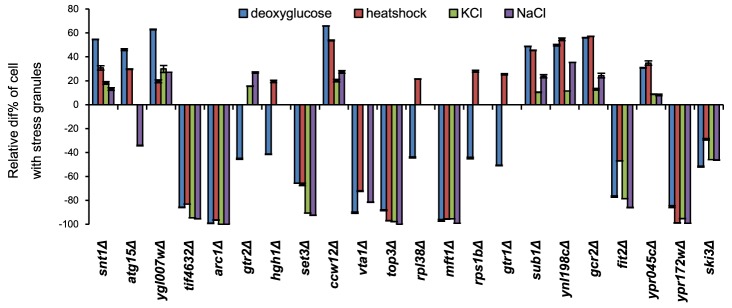
SG phenotypes of individual mutants are mostly general for different stress types. The indicated mutant cells were exposed to the following stress conditions: 400 mM 2-DG (90 min); 44°C (45 min); 1.5 M KCl (90 min); 1.5 M NaCl (90 min). SG phenotype is shown as the deviation from wt level (*his3Δ*) scaled as in Fig. 1 C. Error bars indicate standard error for 25 independent determinations (about 80 cells per image) for each data point.

It is known that defects in a particular stress signaling pathway can affect SG formation ability specifically for that stress condition [24] whereas other mutations cause a general SG deficiency. We investigated the SG phenotype of the 194 mutants using other stress conditions: heat shock (44°C for 45 min) or hyperosmosis (1.5 M KCl or 1.5 M NaCl for 90 min). As seen in Fig. 2 and Fig. S4, some of the mutants display a strong phenotype uniformly across all the investigated stress conditions (*e.g. tif4632Δ*, *arc1Δ*, *set3Δ*, *top3Δ*, *mft1Δ*, *ypr172wΔ*, and *ski3Δ*). For *tif4632Δ* (lacking the gene encoding eIF4G2, one of two isoforms of the translation initiation factor eIF4G in yeast), a defect has previously been observed in glucose starvation [11]. However, a substantial fraction were specific to either 2-DG or to various combinations of the stress conditions (*e.g. gtr2Δ*, *ncs6Δ*, and *hgh1Δ*) (Fig. 2, Fig. S4). Among the set of 73 mutants with verified phenotypes in 2-DG, 10 showed defects under all four stress conditions, and a further 10 under at least three of the four conditions. Mutants with a strong phenotype in 2-DG were more likely to display phenotypes also in other stress conditions (Table S1). Over half of the mutants, 40, were unique to 2-DG. The greatest degree of overlap with the 2-DG SG phenotype was found with NaCl stress (24 mutants); all the mutants that displayed a defect in KCl were also defective in NaCl (Fig. S4).

### Mutants with defective SG formation represent dissimilar functional classes, with enrichment of functions in translation and RNA metabolism

**Figure 3:**
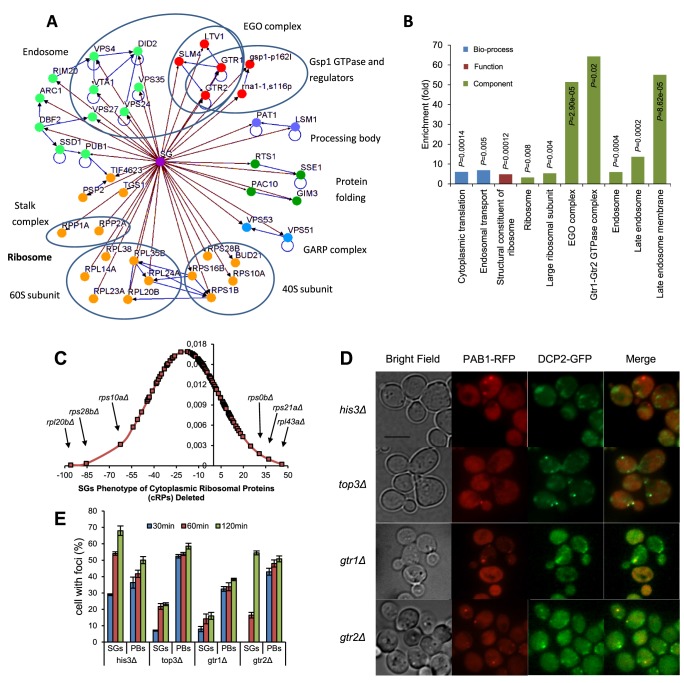
Overview of screening results. **A**) Network analysis of SG-defective mutants. Mutants showing SG formation defect (nodes linked with brown arrows) were grouped into modules based on their known physical interactions (blue arrows) and published cellular components information. Functional groups and cellular components are shown in different colored nodes. Blue circles indicate subunits or complexes within groups. **B**) Functional enrichment analysis of SG-defective mutants. Confirmed SG-defective mutants were analyzed for enrichment of the GO biological process, function and component categories. Enriched groups were scored by comparing to a background list of SGA-V2+slower array (4691 genes) using a cut-off of P<0.05. **C**) Distribution of SG phenotypes among cytoplasmic ribosomal protein mutants. **D,E**) Formation of PBs is undisturbed in the SG-defective mutants studied after addition of 400 mM 2-DG in wt and mutant cells. Data represent mean ± standard error of measurement of three independent determinations. **D**) Micrographs of mutants expressing both Dcp2-GFP (PB marker) and Pab1-RFP (SG marker) **E**) Time course of appearance of SGs and PBs.

A survey of this group of mutants with a verified decrease in SG number reveals a heterogeneous composition of biochemical and cellular functions, a reflection of the complexity of the functions required to assemble SGs, and to regulate their formation under different stress conditions. The network overview of functional clusters in this group (Fig. 3 A) displays large groups related to protein translation and endosomal vesicles, and the interrelated groups of the EGO/GSE complex and the Ran GTPase (Gsp1) with regulators.

GO Term analysis showed that there was a considerable enrichment for gene products involved in cytoplasmic protein translation (12/73, 16.4% vs. 2.4% in the background; P = 2.8×10^−5^). Among these, there is a moderate enrichment of mutants lacking cytoplasmic ribosomal proteins (cRPs). The distribution of relative SG scores of all cRP mutants (Fig. 3 C) contains both increased and negative values; the average value being slightly negative. A few cRP mutants are outliers with strong SG defects, notably *rpl20bΔ*, *rps10aΔ*, and *rps28bΔ*. Mapping the physical position of the corresponding proteins in the ribosome, we observe that these three are scattered in the 40S and 60S subunits (Fig. S5). The location thus does not immediately explain why they are so strongly required for SG formation. Further, the *tif4632Δ* mutant is almost completely devoid of SGs, in line with previous observations [11]. Another strongly SG-defective mutant implicated in translation is *arc1Δ* (human AIMP1), lacking a tRNA synthetase-binding protein.

Mutants representing members of the overlapping EGO/GSE complexes were also notable in this group (*gtr1Δ, gtr2Δ*, *slm4Δ*, *ltv1Δ*; 4/73, 5.5% vs. 0.1% in the background; P = 5.4×10^−6^). The members of the EGO complex were found in a genetic screen for failure to resume growth after rapamycin treatment [28]. Finally, we noted that gene products involved in endosomal transport were enriched in this mutant set (8/73, 11.0% vs. 1.2% in the background; P = 6.6×10^−4^).

### Gene products absent in mutants with increased SG formation are enriched for microautophagy and are located in intracellular vesicles

In the screen, we also observed a large number of mutants with an increased fraction of SG-positive cells (≥20% higher than wt), indicating either a deregulation in SG formation or a defect in SG breakdown. To verify that the increased number of Pab1-positive aggregates seen in these mutants really represent *bona fide* SGs, we pretreated six of them with cycloheximide (Chx) before, as well as during, exposure to 2-DG, a scheme previously shown to prevent SG induction by stress [29]. As expected if they are actually SGs, the Chx pretreatment did completely block formation of aggregates in these mutants (Fig. S6). GO term analysis of the 56 mutants in this group that were confirmed by manual analysis (Table S2 B) revealed that several of them are linked to autophagy/microautophagy (Atg15, Vam6, Vam7, Vtc4, Meh1, Ypt7, Pep4; 7/56, 12.5% vs. 0.6% in the background; P = 1.4×10^−5^). Many of the mutations are in genes encoding proteins located in internal membranes; the vacuolar membrane (8/56, 14.3% vs. 2.4% in the background; P = 4.9×10^−3^) or the ER (5/56, 8.9% vs. 0.8% in the background; P = 5.2×10^−3^). The ontology of this mutant group is thus largely compatible with defects in breakdown of SGs using vesicular transport and autophagy/microautophagy. We did not consider this mutant group further in this report.

### PB formation is intact in most SG-defective mutants

The functional interplay between SGs and PBs is not fully characterized; one model is that formation of SGs is secondary to formation of PBs [11]. If so, we should not necessarily expect all SG-defective mutants to have defective PB formation as well.

To examine whether this holds true for the mutants with impaired SG formation found in our genome-wide screen, we transformed selected mutant strains with a plasmid [30] expressing the PB marker Dcp2-GFP. After glucose deprivation, the localization of Dcp2-GFP was in one to five spots per cell, in some cases in the immediate vicinity of Pab1-RFP, as expected (Fig. 3 D). For *gtr1Δ*, *gtr2Δ*, and *top3Δ* mutants, PB formation was essentially normal, except for a slight depression in *gtr1Δ* mutants (Fig. 3 E). Thus, our observations do not contradict the view that SG formation is contingent on PB formation, but not *vice versa*.

### Low-complexity regions are enriched in the proteins required for SG formation

The question whether there are common sequence motifs and other structural features of proteins contained in SGs or essential for formation of SGs has attracted interest since the realization that TIA-1 and TIA-R, both containing prion-like aggregation-driving domains, are critical for assembly of mammalian SGs [21]. Low-complexity regions (LCRs) in RNA-binding proteins (RBPs) make them prone to aggregation *in vitro* [31].

We wanted to test these suppositions in our large set of SG defective null mutants obtained from an unbiased genome-wide screen. Analysis of the number of LCRs [32] in the proteins deleted in the 60 mutants with the strongest SG-defective phenotype shows that 100% of those contain at least one LCR, vs. 70% of all yeast proteins. This protein group contains on average 4.1 LCRs per molecule, vs. 2.1 for all yeast proteins, and LCRs are thus strongly enriched among these proteins (Table 2). It has also been proposed that intrinsically disordered domains (IDRs) are essential to drive the aggregation process in SG formation [33]. We examined the *S. cerevisiae* genes predicted by Malinovska et al. [33] to encode proteins disordered in their entire length, but did not find them to be enriched among our SG-defective mutants (Table 2). However, a moderate overrepresentation of proteins containing IDRs longer than 30 amino acid residues [34] was observed (Table 2). Only one of the SG-defective proteins (Bem2) was found to carry a prion-like domain [35]. Thus, our data gives support for a possible role of LCRs in SG formation, but not for the other putative aggregation-driving protein domains.

**Table 2:** Occurrence of low-complexity regions (LCRs), intrinsically disordered domains (IDRs), disordered binding domains, and prion-like domains among proteins associated with strong SG-defective phenotypes.

		Total no.	Average no. per gene	Fraction of proteins with at least one domain (%)
**LCRs** [32]	All genes (5888)	12111	2.1	70
	SG-defective (54)*	223	4.1	100
**IDRs** [34]	All genes (5888)	NA	NA	15–45** [74]
	SG-defective (54)	5982	111	59**
**IDPs** *** [33]	All genes (4312)	121	0.028	
	SG-defective (194)	5	0.026	
**Prion-like domains** [35]	All genes (5888)	NA	NA	NA
	SG-defective (54)	1	0.02	2

Domains were identified as previously described: LCRs [32], intrinsically disordered domains (IDRs) [33], [34], and prion-like domains [35].

* Domains are shown in Fig. S9.

** Fraction of proteins with at least one domain over 30 residues long (%).

*** Intrinsically disordered proteins; proteins predicted to be disordered throughout their entire length.

NA, not analyzed.

### Low SG formation ability confers high mutability, but only in specific mutants

**Figure 4:**
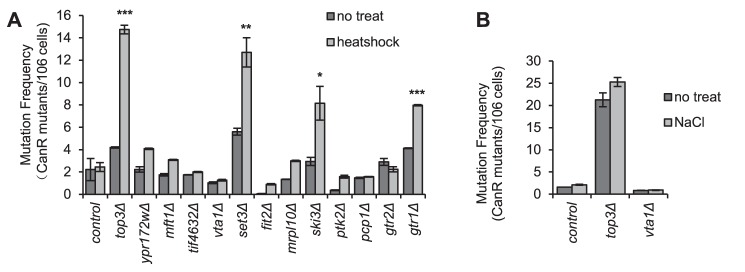
Stress-induced mutation rate in strains with SG defects. The indicated mutant strains, selected for having strongly reduced ability to accumulate SGs (Fig. 2, Fig. S4) were shocked by either heat or NaCl. The frequency of Can^R^ cells was recorded thereafter as detailed in Materials and Methods, and compared to the mutation frequency of untreated cells of the same strain. **A**) Mutation rate after heat shock (44°C for 1 h). Statistically significant increases in heat-induced mutation rate in mutants compared to wt are marked with the respective numbers of stars. P values are listed in Table S3. **B**) Mutation rate after NaCl shock (1.5 M for 1 h). Data are mean ± standard error of measurement of three independent determinations.

Heat stress increases mutation frequency in budding yeast, which has been suggested to result from increased ROS production in mitochondria [36]. This mutation peak can be suppressed by overexpression of Pbp1 or Dhh1, both of which cause increased SG formation [15]. The authors concluded that SGs formed under stress serve as a dampening factor of some aspects of the heat-induced stress response, which attenuate the mutagenic effects of the heat shock. We were interested to see if the converse was true, that the absence of SGs under stress *per se* would enhance the mutation rate. We also wanted to investigate the generality of this phenomenon, and so recorded the frequency of inactivating mutations in the *CAN1* gene, after heat shock (60 min at 44°C, as applied by Takahara, 2012 [15]) for 12 mutants with strongly negative SG phenotypes upon heat shock (Fig. 2, Fig. S4) but otherwise without any obvious functional bias. As seen in Fig. 4 A, indeed a marked increase in canavanine resistant (can^R^) clones (3–15 fold) was observed for four of the mutants; *top3Δ*, *set3Δ*, *ski3Δ* and *gtr1Δ*. However, for the remaining 8 mutants, no significant increased mutation frequency was found; thus, an increased heat-induced mutation rate does not occur in all SG-defective mutants. We wanted to see if other stress types normally causing SG formation would similarly lead to an elevated mutation frequency in SG defective mutants. Thus, we examined *top3Δ* and *vta1Δ* mutants for the frequency of can^R^ clones after hyperosmotic shock (1.5 M NaCl). Both mutants are unable to form SGs under these conditions (Fig. 2). However, no increased mutation frequency was observed neither in the wt nor in either mutant (Fig. 4 B), indicating that the stress-induced mutability may be specific to heat shock.

### Mutants affected in regulation of nucleocytoplasmic RNA transport through the Ran GTPase are defective in SG formation

**Figure 5:**
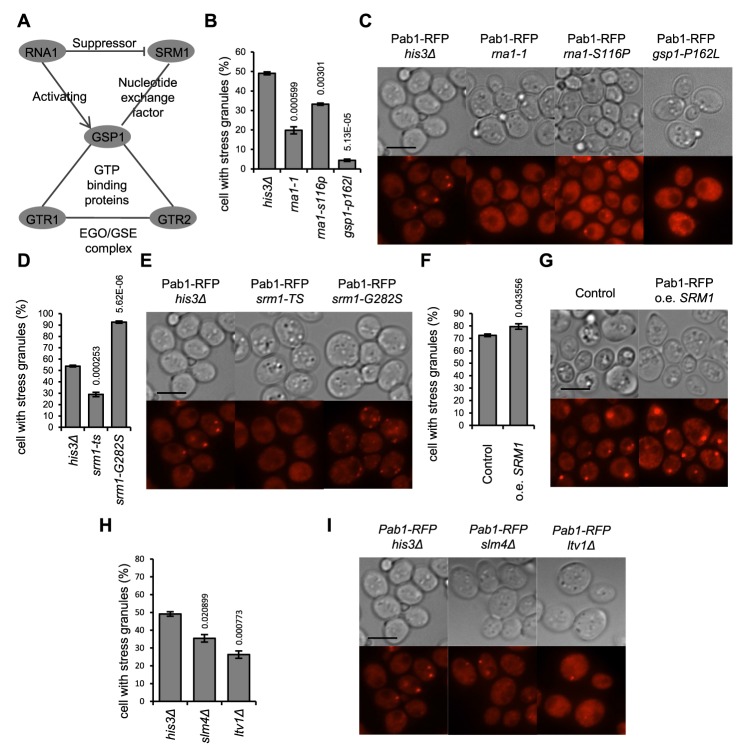
Mutants defective in control of the Ran GTPase homolog have a deregulated SG stress response. **A**) Schematic representation of the relations between the proteins concerned. **B–I**: SG phenotype was recorded after 90 min in 400 mM 2-DG. Numbers above bars in diagrams indicate P-values for a change relative to the wt phenotype. **B**,**C**) Mutations in the Ran homolog (*gsp1-P162I*) and its GTPase activating protein Rna1 (*rna1-1*, *rna1-S116P*) confer strong SG defects. **D**,**E**) Different mutant alleles of the Gsp1 GDP/GTP exchange factor Srm1 (*srm1-ts*, *srm1-G282S*) have opposing effects on SG formation. **F**,**G**) Increased *SRM1* dosage enhances SG accumulation after challenge with 400 mM 2-DG. **H**,**I**) SG phenotypes of other mutants (*slm4Δ*, *ltv1Δ*) functionally linked to the EGO/GSE complex and to *gtr1*/*gtr2* but not to Ran^GDP/GTP^ function. In A, C, and F, data are mean ± standard error of measurement of three independent determinations; in E, five independent determinations.

Gtr1 and Gtr2 have been implicated both in activation of TORC1 as members of the GSE complex [37], and as regulators of the budding yeast Ran homolog, Gsp1, required for a large fraction of all nucleocytoplasmic transport of protein and RNA in the cell. We wanted to examine if the SG defects of *gtr1Δ* and *gtr2Δ* mutants were due to interactions with the TOR pathway, with Ran functions, or both. As many of the Ran function genes are essential (*GSP1*, *RNA1*, *SRM1*), we resorted to temperature-sensitive (ts) alleles of those [38]. Cells carrying the *gsp1-P162I* allele are highly defective in SG formation in glucose starvation at the restrictive temperature (36°C) (Fig. 5 B). *RNA1* encodes the GTPase-activating protein for Gsp1 (RanGAP). The *rna1-1* allele likewise confers decreased SG numbers; the *rna1-S116P* only moderately so (Fig. 5 B). Srm1 (Prp20) encodes the guanine nucleotide exchange factor (GEF) of Gsp1 (RCC1 in human cells). In glucose starvation, the *srm1-G282S* allele instead causes a marked increase of the SG-positive fraction, to 90% of all cells, whereas the *srm1-ts* allele gives lower SG numbers (Fig. 5 D). These results show that those mutants with a functional connection both with Gtr1 and Gtr2 and with the Ran nucleocytoplasmic transport system, are also affected in their SG forming ability. The other EGO/GSE mutants, *slm4Δ* and *ltv1Δ*, also did display reduced SG numbers, though with weaker phenotypes (Fig. 5 H,I).

### Strains with *gtr1* and *gtr2* mutations recover growth slowly and lose viability faster after glucose starvation

**Figure 6:**
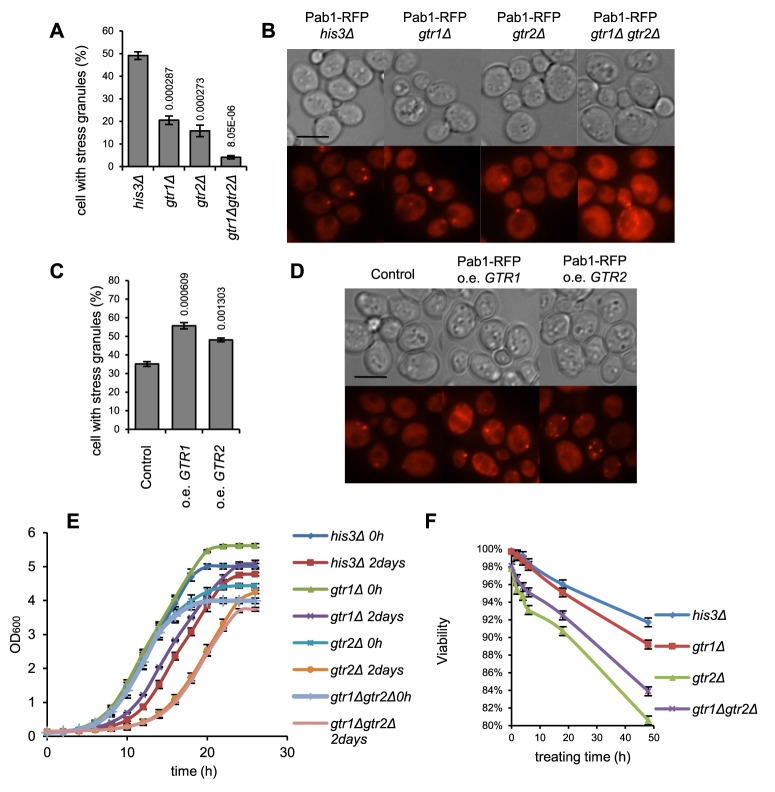
SG formation, recovery and post-stress survival is related to gene dosage of *GTR1* and *GTR2*. **A**,**B**) The *gtr2Δ* mutant has a strong SG defect on glucose starvation, which is aggravated in the *gtr1Δ gtr2Δ* double mutant. The SG phenotype was assessed 90 min after exposure to 400 mM 2-DG. **C**,**D**) Increased dosage of *GTR1* or *GTR2* confers an increased SG response; stress treatment as in A and B. **E**) The *gtr2Δ* mutant and the *gtr1Δ gtr2Δ* double mutant display delayed recovery of growth after glucose starvation. Cells were grown to mid-log phase, and either transferred to SC medium with 400 mM 2-DG for 48 h and then diluted into medium with 2% glucose, or diluted straight into glucose-containing medium. Growth curves were recorded in a BioScreen™ as described [71]. **F**) The *gtr2Δ* mutant and the *gtr1Δ gtr2Δ* double mutants lose viability under glucose starvation. Mid-log phase cells were transferred to SC medium with 400 mM 2-DG at 20°C and kept for the indicated times, before plating on SC agar medium with glucose but without 2-DG for viability counting. E,F: average of four (E) or two (F) independent experiments are shown. Error bars indicate standard error.

It was previously shown that *gtr2* mutants recover slowly from rapamycin-induced arrest, mimicking a state of amino acid starvation [28]. More recently, the mammalian Gtr1 homolog RagA was also implicated in glucose homeostasis [39]. Since SGs are presumed to play a regulatory role in the transitions between stress and favorable conditions, and given the defects in glucose starvation-induced SG formation that we observed in the *gtr1* and *gtr2* mutants, we wanted to see if this defect would affect growth and survival after glucose starvation in these single mutants and also in the double mutant, *gtr1Δ gtr2Δ*. First, we examined the *gtr1Δ gtr2Δ* mutant SG phenotype in glucose starvation (Fig. 2 A, B) which was found to be more exacerbated than in the single mutants. To analyze the growth behavior, cells in log phase (wt, *gtr1Δ*, *gtr2Δ*, or *gtr1Δ gtr2Δ*) were glucose-starved for 48 h by addition of 2-DG, and the rate of recovery of growth in medium without 2-DG was recorded. We found that both the *gtr2Δ* and the *gtr1Δ gtr2Δ* mutants recover appreciably slower than the wt (Fig. 6 E). We also investigated loss of viability of the same strains in medium containing 2-DG. As seen in Fig. 6 F, loss of viability occurs fastest in the *gtr2Δ* and the *gtr1Δ gtr2Δ* mutants. We conclude that Gtr1 and Gtr2 affect growth recovery and maintenance of viability in glucose starvation, with Gtr2 having the bigger effect.

### SG formation defects exaggerate stress-induce gene expression changes

It has also been shown that SGs physically incorporate, and potentially dampen the activation of, signaling proteins [15], [16], [40]. Now having access to mutants deficient in SG formation from an unbiased genome-wide screen, we wanted to test the possibility that the SG-forming ability under stress is mechanistically linked to the ability to raise and regulate various aspects of the cellular stress response. To do this, we investigated the expression pattern of stress-responsive genes in mutant backgrounds which we had shown to be unable to form SGs under different stresses (heat shock, hyperosmotic shock, and glucose deprivation) (Fig. 2, Fig. S4).

**Figure 7:**
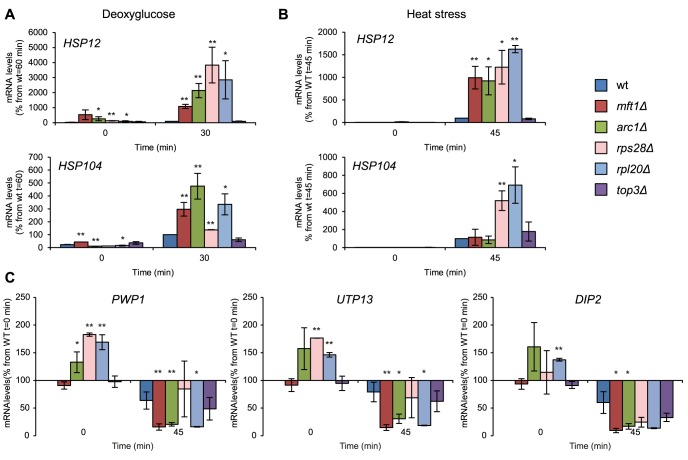
Deregulated stress-induced gene expression in SG-defective mutants. Gene expression analysis for stress-induced (A, B) and stress-repressed genes (C) in wt and SG-defective mutants (*mft1Δ*, *arc1Δ*, *rps28bΔ*, *rpl20bΔ*, *top3Δ*) exposed to stress. The degree of induction or repression of stress-responsive genes was determined using qPCR. To normalize, the levels of *ACT1* mRNA was used as a reference, and final values were represented as a percentage of the maximum value in wt cells as indicated in each graph. Data represent at least two independent experiments. Data points and error bars show mean and standard deviation. Statistical analyses were performed using Student's t-test of comparisons between wt and mutants in the same time point (* p<0.05, ** p<0.01). **A**) Induction of the *HSP12* or *HSP104* transcripts in cells exposed to 400 mM 2-DG. **B**) As in A, but cells were exposed to heat shock (44°C). **C**) Repression of the *PWP1*, *UTP13* and *DIP2* transcripts after 45 min at 44°C.

Hsp12 changes its conformation and associates with the plasma membrane to stabilize it under stress. Hsp12 is strongly upregulated under several stresses, including heat shock, hyperosmotic shock and glucose deprivation, and is regulated through the RAS-PKA pathway [41]. We measured induction of *HSP12* expression in *mft1Δ*, *arc1Δ*, *rpl20Δ*, *rps28Δ*, and *top3Δ* mutants after two different stress conditions: 2-DG (400 mM) and heat stress (44°C). In four out of these five mutants (*mft1Δ*, *arc1Δ*, *rpl20Δ*, and *rps28Δ*), we observed a dramatic hyperinduction of *HSP12*. At 60 min. after addition of 2-DG, the level of *HSP12* mRNA was between 10 and 40 times higher than in the wt (Fig. 7 A). Similar results were obtained by heat shock: after 45 min at 44°C, the *HSP12* mRNA was upregulated in the same four SG-defective mutants to levels 10 to 16 times higher than in the wt (Fig. 7 B). The *HSP104* transcript in the same mutant set followed a related pattern in glucose starvation and heat shock as *HSP12* (Fig. 7 A,B).

*PWP1*, *UTP13*, and *DIP2* all encode proteins involved in rRNA processing, and form part of the RiBi regulon [42]. They are under positive control by the TOR pathway via protein kinase Sch9 [43]; under stress conditions their expression decreases concomitant with diminished TOR signaling. When challenged by heat stress, the expression response in these mutants (same set as in Fig. 7 A) is similar for all three reporter genes (Fig. 7 C). Thus, for *PWP1*, *UTP13*, and *DIP2* alike, the mRNA levels decreased to 65–80% of their pre-stress levels at 45 min. at 44°C in the wt. In contrast, the expression of *PWP1* and *UTP13* in *mft1Δ*, *arc1Δ*, *rps28Δ*, and *top3Δ* mutants in heat stress decreased to between 10 and 60% of the level in undisturbed wt cells. The expression of *DIP2* was more reduced than in wt in all the mutants, including *rpl20Δ*. In summary, gene expression response to heat stress resulting from reduced TOR signaling in these SG-defective mutants (*mft1Δ*, *arc1Δ*, *rps28Δ*, *top3Δ*) is hyperactivated, with the change in expression level between different mutants highly correlated between the three reporter genes.

Having observed deregulation of the stress response in heat shock and glucose starvation, we wanted to also address hyperosmotic shock. *STL1* encodes a plasma membrane-bound glycerol transporter, is strongly induced after hyperosmotic shock, and is controlled by the HOG pathway [44]. After severe hyperosmotic shock (1.5 M NaCl), where SGs are clearly visible, the expression level of *STL1* in wt cells was quite low for an extended time after application of stress (Fig. S7 D). This made it difficult to assess to what extent an induction had taken place, and so we therefore chose to investigate *STL1* mRNA levels after moderate hyperosmotic shock (0.4 M NaCl). Under these conditions, *mft1Δ* and *arc1Δ* mutants demonstrated somewhat elevated levels after stress (Fig. S7 A). We then wanted to see if maybe differences similar to what we observed after heat or glucose starvation stress could be seen at the protein level after hyperosmotic shock.

As the role of SGs in the stress response is generally perceived to be in cytoplasmic silencing and storage of mRNA, it appeared reasonable to also consider the impact on the post-transcriptional level. We tested the induction of β–galactosidase protein from a *STL1-LacZ* promoter fusion construct after moderate (0.4 M NaCl) hyperosmotic shock in the same SG-defective mutant backgrounds as above. As seen in Fig. S7 C, the induction of *STL1*-lacZ upon hyperosmosis is further (1.5–4 fold) elevated in all mutants compared to the wt in the time interval 30–180 min after hyperosmotic shock. The exception is *mft1Δ*, where instead *STL1*-lacZ fails to induce upon hyperosmosis (Fig. S7 B). The levels of *STL1*-driven *lacZ* transcript, however, were not appreciably altered in the mutants (except again in *mft1Δ*). We infer that in the case of *STL1*-lacZ, the deranged regulation is likely to be on the translational level.

As negative controls, we examined in the same fashion mutants with no observed SG phenotype, *ybl059wΔ* and *ybl060wΔ*, as well as mutants with increased numbers of SG, *ccw12Δ* and *ynl198cΔ*. No abnormal *STL1*-lacZ induction was seen in *ybl059wΔ* and *ybl060wΔ*, whereas slightly elevated induction was observed in *ccw12Δ* and *ynl198cΔ* (Fig. S7 C).

We had thus demonstrated that most mutants which show a strongly reduced ability to form SGs upon stress, either induce stress response genes excessively, or in exceptional cases (*STL1*-lacZ induction in *mft1Δ*) are unable to mount a stress-activated gene expression response at all. It also appears that this deregulation occurs both at the transcriptional and post-transcriptional levels. We then wanted to determine if these defects were caused by altered activity in the stress-activated signaling pathway upstream of the gene expression response. We examined activation of the HOG pathway, measured as Hog1 phosphorylation, under moderate (0.4 NaCl) or severe (1.5 M NaCl) hyperosmotic shock, in wt and SG-defective mutant cells. We also measured regulation of the TOR pathway through phosphorylation of eIF2α (Sui2) after heat shock in SG-defective mutants. Reduced Tor1 activity leads to increased phosphorylation of Gcn2, the protein kinase targeting eIF2α [45]. No differences in phosphorylation level after stress of either Hog1 or eIF2α between wt and mutants were observed, however (Fig. S8). We conclude that the cause of the deregulated stress-induced gene expression in SG-defective mutants is not alterations in signal transduction *per se*, but must instead lie at the levels of transcription or stability of target mRNAs.

## Discussion

The study of SGs has proceeded mainly through characterization of single proteins that are SG components, or required for SG formation. Our genome-wide screen for mutants defective in SG formation provides an in essence unbiased global view, and has identified proteins of unexpected cellular functionalities. We acknowledge, however, that the screen has technical limitations, and that some mutants could have been scored as SG defective *e.g.* because of low Pab1 expression levels. Conceptually, we can divide the proteins involved in SG formation into at least three classes: (**a**) structural SG components with properties that facilitate assembly of the entire SG (“drivers” of SG formation). Examples are the mammalian TIA-1 and TIA-R proteins, which carry prion-like domains that promote aggregation [21]. Defective expression of those confers decreased SG formation. (**b**) “Passenger” proteins that are found in SGs but whose absence does not confer overall SG defects. The fission yeast multi-KH domain protein Vgl1 is found in SGs, but deletion of the corresponding gene does not abolish SG formation [23], providing an example of this second class. (**c**) Members of signaling pathways required for SG formation under specific stress conditions. Subunits of both PKA [40] and TOR kinase [15] have been localized to *S. cerevisiae* SGs. By definition, then, only members of groups (a) and (c) could score in our screen as deficient in SG formation. Using cross-linking to mRNA and mass spectroscopy, Mitchell et al. [46] recently identified 120 RNA-binding proteins in yeast, of which 36 were localized to SGs and/or PBs. Out of these 36, three (Psp2, Bre5, and Tif4632) also turned up in our mutant screen as SG defective (Table S1). They are thus candidate members of group (a) as their absence from SGs prevents SGs from forming at all.

It is further interesting to compare our mutant set with the human genes previously identified in an RNAi screen for reduced SG formation [14] (Tables S1 and S2). Direct gene-to-gene ortholog searches revealed knockdown of *G3BP1* (ortholog *BRE5*) or *PDCD6IP* (programmed cell death 6 interacting protein; ortholog *RIM20*) and mutation in the respective yeast orthologs to cause reduced SG formation in both organisms. In addition, the human genes *RPL37A* (yeast homolog *RPL43A*), *RPL21* (yeast *RPS21A*), and *SMDP3* (yeast *PPN1*) were identified by Ohn et al. (2008) [14]; here however we show mutation of the corresponding yeast orthologs to cause increased SG numbers in 2-DG. In some cases, functionally closely related genes were found to affect SGs in the two organisms: human *CTSC* (cathepsin C) vs. yeast *PEP4* (cathepsin D), human *PPP2R1A* (protein phosphatase 2 regulatory subunit A) vs. yeast *RTS1* (phosphatase 2A regulatory subunit type B′). There are also parallels in functional groups; *e.g.* several different cRPs are found to affect SG formation in both organisms.

In addition, we scored SG hyperproficient mutants. Such mutants could experience a higher general stress level because of an underlying general cellular defect, or could be defective in SG breakdown and turnover. In neurodegenerative disorders, RNA-protein aggregates, assumed to be related to SGs, accumulate in neurons and contribute to pathogenesis [47], [48]. This suggests that also the SG removal process may have implications for health. Recently, it was shown that disassembly of SGs in *S. cerevisiae* occurs through autophagy in a Cdc48-dependent way [27]. In their study, Buchan et al. [27] found an enrichment of mutants deficient in autophagy, vacuolar fusion and related processes among those with increased numbers of spontaneous SGs resulting from defective turnover. We find only two individual mutants in our screen with increased SG numbers after glucose stress to overlap with Buchan et al., namely *ice2Δ* and *meh1Δ* (member of the EGO/GSE complex). However, in our screen we find several representatives of pathways connected to granule disassembly, such as endosomal transport, microautophagy and protein degradation, broadly along the same line as the findings of Buchan et al. [27].

### Are there protein domains driving aggregation and SG formation?

Proteins with an ability to drive SG formation based on their content of aggregation-prone domains could be suspected to act as assembly factors. An examination of the requirements for formation of RNA granules in cell-free systems has identified proteins with low-complexity regions as capable of holding such large structures together [31]. Our finding that LCRs are highly enriched in the proteins affected in SG-defective mutants lends support to this notion that multiple weak interactions between LCRs are a driving force behind aggregation of SGs [31], [49]. On the other hand, only a minority of these SG-defective mutants encode proteins that have been shown to actually reside in SGs. Thus, many could be indirectly acting (class “c”), making the interpretation of this finding less clear.

Mammalian TIA-1 and TIA-R, carrying prion-like domains, have been shown to have a key role in SG formation [21]. Recently, the presence of intrinsically disordered regions (IDRs) in proteins from genetic model organisms has been mapped and proposed as a determining factor for assembly of SGs [33]. We did find a moderate enrichment of IDRs, but not of prion-like domains among the SG-deficient mutants identified here (Table 2). For instance, the translation initiation factor eIF4G has been found to be an SG component in all species studied. We discovered the *tif4632Δ* mutant, lacking the eIF4G2 isoform, to have a strong SG defect. The Tif4632 protein contains IDRs, and it is conceivable that they serve as drivers for nucleation of SGs [8], given this strong requirement of eIF4G2 for SG formation.

### Stress conditions and SG formation

We have found gene products common to all stresses applied, as well as others that are specific to one or few stress agents. Earlier work investigating individual SG components has yielded results in line with this: thus, eIF3 components are found in budding and fission yeast SGs after hyperosmotic shock or heat stress, but not after glucose deprivation [11], [22], [24]. On the other hand, in a directed study in budding yeast of preselected mutant alleles, alterations in the TOR or PKA pathways were reported not to affect formation of SGs under several different stress conditions [12], demonstrating that not every pathway component is necessarily required for SG induction.

SGs formed in budding yeast after NaN_3_ treatment contain translation initiation factor components not found after glucose starvation, including components of eIF3 (Rpg1 and Prt1), eIF4 (eIF1A, eIF4B, eIF5B), and eIF5B [25]. The sets of proteins required for SG formation (assembly factors) under diverse stresses are likewise non-identical. After glucose deprivation, *pbp1Δ* and *pub1Δ* mutants are defective in SG formation, but not after NaN_3_ treatment [25]. In line with this, the majority of the mutants identified in this screen are SG defective in response to some, but not all, stress conditions. However, SG defects common to all stress conditions tested was most frequent among the mutants with the most severe SG defect in 2-DG, suggesting that such mutants lack components fundamental to all SG assembly.

### Elevated stress-induced mutation frequency only in some SG-defective mutants

In four of the tested SG-defective mutants (*top3Δ*, *set3Δ*, *ski3Δ*, and *gtr1Δ*), the induced mutability upon heat stress was substantially increased compared to the wt (Fig. 4). In some cases the change was even higher than what was earlier observed when the SG amount was artificially manipulated by other means (Chx, overexpression of *PBP1* or *DHH1*) [15]. However, we observed no altered induced mutation frequency for the remaining SG-defective mutants. Desequestration of TOR pathway signaling components by the lack of SGs under heat stress does lead to more rapid reactivation of TOR signaling in the recovery phase [15]. Mutants lacking SGs are predicted by the model proposed by Takahara and Maeda [15] to have more TOR signaling components active, and to display an increased mutation rate upon heat stress. However, in view of our present study which extends to more SG-defective mutants, most of which are affected in functions unrelated to the TOR pathway, this does not necessarily confer an elevated heat-induced mutation rate. While our results therefore give some support to the notion that SGs moderate heat-induced hypermutability, it is clear that the mechanism, remains ambiguous, and additional yet unidentified factors are required for this to occur.

### Nucleocytoplasmic transport functions are linked to SG formation

The *gtr1* and *gtr2* mutants both appeared in our screen. The corresponding proteins are closely related unconventional G proteins with metazoan homologs; RagA and RagB (Gtr1-like), RagC and RagD (Gtr2-like) [50]. Gtr1 and Gtr2 are established in at least two plausible roles to affect SG formation. On one hand, they regulate the *S. cerevisiae* Ran homolog Gsp1, and its paralog Gsp2, which are required for the bulk of RNA processing and nucleocytoplasmic transport. *GTR1* was originally identified as a suppressor of a mutant allele of *SRM1* [51]. On the other hand, Gtr1 and Gtr2 are part of the EGO/GSE complex, which is required for microautophagy [28], [37], [52]. EGO/GSE members are Gtr1, Gtr2, Meh1, Slm4, and Ltv1 [28], [37], [52]. EGO/GSE is implicated as a TOR signaling activator. Thus, the mammalian Gtr1/Gtr2 homologs RagA – D bind Raptor (*S. cerevisiae* homolog Kog1) and so mediate signaling from growth-promoting amino acid levels to mTOR [53]. In yeast, Gtr1 in its GTP-loaded state binds Tco89 and this interaction is required for activation of TORC1 [37].

The explanation for the observed SG defect in *gtr1* and *gtr2* mutants could thus lie in their involvement in nucleocytoplasmic RNA transport, activation of the TOR pathway and microautophagy, or both. Mutations along the Ran^GDP/GTP^ axis (*gsp1-P162I*, *rna1-1*, *rna1-S116P*, *srm1-ts*, *srm1-G282S*) invariably affected SG formation (Fig. 5). As haploid null alleles are not available for these genes, it is difficult to judge from the phenotype strength of the different point mutants with various expressivities the exact degree of involvement in SG formation of the corresponding gene product. However, it does appear to be significant, as at least one allele of each of these genes gives a strong SG phenotype, *gsp1* itself strongest among them. On the other hand, mutations in remaining EGO/GSE components (*slm4Δ*, *ltv1Δ*) also affected SG levels, albeit moderately. Recently, the mammalian Gtr1/Gtr2 homologs, RagA – D, were shown to play a role in glucose sensing through the mTOR pathway, as newborn mice expressing the constitutively active form RagA^GTP^ become hypoglycemic [39]. It therefore appears plausible that the effects we observed under glucose starvation in *gtr1* and *gtr2* mutations on SG formation, as well as recovery rate and survival (Fig. 6) are mediated through defective TOR signaling.

There is some support for a nuclear role of EGO/GSE. Thus, Gtr1, Gtr2, Prp20 and Gsp1 co-fractionate with chromatin. The *gtr1* and *gtr2* mutants display derepression at telomeres. Gtr2 physically interacts with Rvb1 and Rvb2, members of the Ino80 chromatin remodeling complex, and *gtr1* is synthetically lethal with *ino80* [54]. Further, Ltv1 has been implicated in nucleocytoplasmic export of the ribosomal 40S subunit [55]. Gtr1 also physically associates with Rpc19, a subunit of RNApolI and RNApolIII, and with Nop8, an rRNA processing enzyme [56]. Gtr1 could thus link ribosome biogenesis with signals for cell growth. In keeping with this interpretation, rRNA synthesis, as well as RNApolIII activity, are reduced in *gtr1Δ* mutants [56]. Gtr1 and Gtr2 could be the focal point of both the TOR signaling and nuclear functions. In line with this, the *gtr1 gtr2* double mutant displayed quite a strong SG defect (Fig. 6 A).

Intriguingly, a majority, 30 out of the 54 genes deleted in the verified SG-defective mutants encode nuclear proteins, whereas only 8 are reported as predominantly cytoplasmic. This points at a role for nucleocytoplasmic communication in regulation of SGs. Previous observations also hint at an essential role for nucleocytoplasmic transport in regulated SG formation. Importins α1, α4, α5, and β1 are located in mammalian SGs [57]. The ubiquitin-like protein Mdy2 (homologous to human Rad23) localizes to heat-induced budding yeast SGs, and is required for the heat-induced stress response. Interestingly, removing the nuclear localization signal from Mdy2 prevents its association with Pab1 in SGs [58], indicating that nucleocytoplasmic transport of Mdy2 is essential for its role in the heat-induced SGs and stress response.

### SG formation and stress-induced gene expression are linked processes

Recent data indicate that the fate of a transcript is determined by RBPs associating with the nascent mRNA molecule, a process dubbed co-transcriptional mRNA imprinting. Cytoplasmic mRNA degradation factors shuttle into the nucleus to bind to chromatin, thereby enhancing the synthesis rate of novel transcripts [59]. In this view, nucleocytoplasmic transport of RNA and proteins is central to control of gene expression. As stated above, we have implicated the Ran-based system for nucleocytoplasmic transport in SG formation (SG phenotypes found in *gtr1*, *gtr2*, *gsp1*, *rna1*, and *srm1* mutants). It likely that formation of SGs, which sequester many RBPs including mRNA degradation factors, will dampen their cycling back into the nucleus. Thus, in the absence of SGs, more degradation factors and other RBPs will be free to move into the nucleus. If this cycling of RBPs into the nucleus is required to mount a transcriptional response upon stress, then this may be the explanation why we observe a hyperinduction of stress-activated mRNAs in SG-defective mutants (Fig. 7 A, B). Conversely, in the case of stress-repressed mRNAs that are hyperrepressed in SG-defective mutants (Fig. 7 C), RBPs with a role in repression may accompany the transcript.

This model then predicts that every SG-defective mutant should display deregulation of stress-regulated genes. The number of mutants investigated here is insufficient to establish this as a general principle but the model appears viable for a large fraction of SG-defective mutants. The *top3Δ* mutant did not conform fully to this pattern however; while it did display hyperrepression (Fig. 7 C), as well as hyperinduction of *STL1*-lacZ in severe hyperosmosis (Fig. S7 D), it induced the *HSP12* and *HSP104* genes similar to wt (Fig. 7 A,B). It remains a possibility that certain SG-defective mutants, where the fundamental role of the cognate protein is in other cellular functions than RNA-related processes (topoisomerase III is primarily implicated in DNA recombination), are not affected in stress-related transcription.

We therefore propose a view where, not only do stress-responsive signaling pathways affect SG formation, but SGs themselves can also influence the outcome of the stress response. In support of this, it was recently shown that in mammalian cells, formation of large SGs by overexpression of G3BP takes place before eIF2α phosphorylation through protein kinase R. In this setting, formation of SGs can be the cause, but not the effect, of eIF2α phosphorylation [60]. Sequestration of signaling components in SGs has previously been proposed to affect cytoplasmic signaling events from the TOR pathway in budding yeast [15], or MTK1-SAPK in mammalian cells [16]. Our data indicate that such effects also can influence the levels of stress-related transcripts. This could occur through increased transcription rates or increased mRNA half-lives; both mechanisms are compatible with our data. Given the requirement for nucleocytoplasmic traffic for SG formation evident from previous and our present data as discussed in the above section, it is natural to speculate that nuclear events are also involved, through the action of RBPs shuttling into the nucleus.

Our global genetic screen has unraveled several unforeseen components required for regulated SG accumulation. We found *arc1Δ* mutants to be almost completely unable to form SGs under any stress condition tested (Fig. 2). *ARC1* encodes a tRNA and aminoacyl-tRNA synthetase binding protein [61], and is the only gene with a specific role in tRNA metabolism to appear in our screen with a strong SG-defective phenotype. This finding is intriguing, as the mammalian stress-activated ribonuclease angiogenin cuts tRNAs, generating small RNA fragments that inhibit initiation of translation, and are essential for angiogenin-dependent SG accumulation [62]. Arc1 has no sequence similarity to angiogenin and has no recognizable RNase domains. However, it is conceivable that it performs another function in an analogous process in yeast. Elucidation of the role of Arc1 and the other ostensibly diverse proteins identified here by genetics as required for stress-induced SG formation, will lead to a broader appreciation of the function in stress response modulation of these large cellular aggregates, which have so long defied exhaustive biochemical characterization.

## Materials and Methods

### *S. cerevisiae* strains and growth conditions

Strains were from the BY4741 background and were grown at 30°C or at the indicated temperatures. Cells were grown in synthetic defined (SD): 0.14% yeast nitrogen base (YNB; Difco) without amino acids; 0.5% ammonium sulphate; 1% succinic acid; 2% D-glucose; 20 mg/l each of histidine, methionine, lysine, and uracil; 100 mg/l of leucine (pH 5.8), or in synthetic complete (SC); SD plus 0.079% synthetic complete drop out mix (Difco).

### Construction of a genome-wide collection of *S. cerevisiae* haploid deletion mutants expressing Pab1-RFP

To efficiently incorporate the SG marker (*Pab1-RFP*) into the yeast single deletion collection (SGA-V2), we applied the yeast synthetic genetic array (SGA) methodology, which allows the *Pab1-RFP* marker to be combined into a single haploid cell through standard mating and meiotic recombination *via* a robotic SGA procedure [63], [64] using the Singer RoToR HDA system (Singer Instruments).

### Increased gene dosage

Wt cells were transformed with the centromeric vector p5586 [65] carrying the gene of interest including its native promoter and terminator sequences, and compared with wt cells carrying empty p5586.

### Genome-wide high content screening with quantitation of SG numbers

Gene deletion strains expressing Pab1-RFP were transferred to 96-well microtiter plates and frozen at −80°C. Prior to screening for SG formation, one 10 µl sample per strain was allowed to grow in 200 µl of SD at 30°C for 2–3 days without shaking. Then, each preculture was diluted 75-fold into a final volume of 215 µl of SD medium and grown with shaking at 30°C. After 16 h, when the OD_600_ of most wells had reached 0.5, 2-deoxyglucose (2-DG) was added to a final concentration of 400 mM. After 2 h of incubation with continued shaking, cells were fixed for 30 min. at room temperature by adding formaldehyde to a final concentration of 3.7%. Cells were then washed twice with PBS and stored at room temperature in 200 µl PBS. For quantification of SGs, fixed cells were imaged in ImageXpress MICRO (MDC), automated cellular imaging and analysis system. After image acquisition, images were quantified by MetaXpress (Version 3.1) software. A MetaXpress sub-program was designed for the quantification of SG phenotypes, which has the ability to automatically query images from our database, extract key SG morphology features and export the measurements as a separate output. For manual quantification, cells were grown in logarithmic phase in 15 ml Falcon tubes to an OD_600 nm_ of 0.5 before application of stress, formaldehyde-fixed and washed as described above before microscopy.

### Functional enrichment and interaction network analysis

The functional enrichment analysis was based on the result from Gene Ontology Term Finder [66], and P-values were calculated using a hypergeometric distribution with multiple hypothesis correction. Manually confirmed hits were analyzed for enrichment of Gene Ontology (GO) biological processes, molecular function, and cellular component categories by comparing with a background set list representing SGA-V2 plus an array of slow-growing mutants (4691 genes). A cut-off of P<0.05 was used. The interaction network diagram of SG-defective mutants was extracted from the interaction analysis using Osprey 1.2.0 [67] and the physical interactions between confirmed hits were added according to the BioGRID interaction database [68].

### Bioinformatic analyses

To identify domains implicated in protein aggregation several bioinformatics algorithms was used: GBA [32] for low-complexity regions (LCRs), IUPRED and ANCHOR [33], [34] for intrinsically disordered domains (IDRs) and PAPA [35] for prion-like domains.

Visualization of the position of cytoplasmic ribosomal proteins in the ribosome was done in UCSF Chimera [69]. Ribosome structures (40S subunit; PDB ID: 3U5G; 60S subunit PDB ID: 3U5I) [70] were downloaded from the RCSB Protein Data Bank (http://www.rcsb.org/pdb/home/home.do).

### Determination of growth rate and lag phase after recovery from glucose deprivation

Determination of growth parameters was performed in a Bioscreen Analyzer V as described [71] after recovery from treatment with 400 mM 2-DG. Strains were run in 5 duplicates on separate plates, with five wild-type (wt) strains on each plate as controls.

### Mutability assay

Induced mutation frequencies were estimated as the forward mutation frequency of the *CAN1* gene [72]. Wt or mutant cells were grown in SC until late log phase (OD_600_ = 1.0) and exposed to stress as indicated. Cells were washed with sterile water and plated on SC lacking arginine (SC-Arg) with or without L-canavanine sulfate (60 µg/ml). Canavanine-resistant colonies were counted after 7 days at 30°C and normalized for total viable cells on SC without canavanine.

### Quantification of stress-induced gene expression and signaling

Wt or mutant cells were treated as specified for different time periods, and subsequently flash-frozen on dry ice/ethanol and harvested. mRNA levels of the indicated stress-responsive genes were determined using real-time quantitative PCR (qPCR). Total RNA was obtained from cell pellets using phenol-chloroform extraction and ethanol precipitation. RNA was treated for 15 min at 25°C with DNase I RNase-free (Roche) according to the manufacturer's protocol. Then cDNA was synthesized in 20 µl reactions containing 25 ng/µl of DNase I treated RNA, 5 µM of Oligo d(T) (Thermo Scientific), 10 units/µl of SuperScript II Reverse Transcriptase (Invitrogen), 1× First Strand Buffer, 10 mM DTT, and 0.8 mM dNTPs. Afterwards, qPCR was performed in a reaction final volume of 10 µl using the Eva Green (Solis) for fluorescent labeling, 2.5 µl cDNA and 0.2 µM of the corresponding oligonucleotides. Real-time PCR reactions were performed under the following conditions: 95°C for 15 min to activate the polymerase, followed by 40 cycles of 10 s at 95°C, 20 s at 60°C and 10 s at 72°C. At the end of the amplification cycles, a melting curve analysis was conducted to verify the specificity of the reaction. For each analyzed primer pair, a negative control was included and a standard curve was made with serial dilutions of cDNA samples pool (1/2, 1/5, 1/10, 1/25, 1/50, 1/100 and 1/500).

Alternatively, the protein product level was determined using a lacZ assay, where β-galactosidase activity was determined and normalized for cell number as described [73].

Hog1 Tyr176 phosphorylation was assayed by western blot using α-phospho-p38 antibodies (Cell Signaling Technologies). eIF2α (Sui2) Ser51 phosphorylation was determined using α-phospho-eIF2α antibodies (Biosource).

## Supporting Information

Figure S1 Co-localization of Hsp104 and Pab1 after different types of stress. Wild-type cells (*his3*) containing the genomic tags Pab1-RFP and Hsp104-GFP was grown at 30°C in synthetic defined media until exponential phase (OD_600_ = 0.5). Then, cells were allowed to grow to stationary phase overnight, exposed to heat shock (44°C, 45 min), osmotic stress (1.5 M NaCl or 1.5 M KCl, 90 min), or heat plus glucose starvation (400 mM 2-DG at 44°C, 45 min). Non-stressed cells were included as a control. After incubation with continued shaking, cells were fixed and imaged as described in Materials and Methods.
(PDF)

Figure S2 Pab1 levels in SG-defective mutants. Wt (*his3*) or mutant cells expressing Pab1-RFP were grown at 30°C in synthetic defined media until exponential phase (OD_600_ = 0.5). Half of each culture was then either treated with 400 mM 2-DG for 90 min, or grown for the same time with addition of 2-DG. After western blot, protein extracts were probed by α-RFP antibodies (GenScript) and using α-Pgk1 antibody (Abcam) as a loading control.
(PDF)

Figure S3 Detection of Pbp1-positive SGs in mutants with SG defects or supernumerary SGs. Strains were transformed with a plasmid expressing Pbp1-mCherry (Text S1) and grown at 30°C in synthetic defined medium without uracil until exponential phase (OD_600_ = 0.5). Glucose was removed by washing the cells twice with medium without glucose and inoculating in the same medium, and samples were taken at the indicated times thereafter. For quantification of the fraction of Pbp1-positive SGs, unfixed live cells were imaged in Axio Vision and manual quantification of number of cells containing SG was performed (a total of 100–300 cells from were counted for each sample). Values represent two independent experiments; range indicates standard deviation. SG-Pab1 defective mutants: *arc1Δ*, *top3Δ*, *hgh1Δ* and *vam3Δ*. SG-Pab1 supernumerary mutants: *brp1Δ*, *ccw12Δ*, *pep4Δ* and *ric1Δ*.
(PDF)

Figure S4 SG phenotypes in different stresses. **A**: Mutants were selected that had>20% SG phenotype deviation from the wt in the large-scale screen in 2-DG, and where the difference was statistically significant (P<0.05). Cells were exposed to the indicated stress conditions: 400 mM 2-DG for 90 min; 44°C for 45 min; 1.5 M KCl for 90 min; or 1.5 M NaCl for 90 min, and the SG phenotype quantitated. **B**: Venn diagram showing how SG-defective phenotypes in different stress conditions are shared in individual mutants.
(PDF)

Figure S5 Position in the ribosome of cRPs associated with strong SG defects. Red, Rpl20b; green, Rpl24a; blue, Rps10a. 60S subunit is shown in cyan; 40S subunit in beige.
(PDF)

Figure S6 Cycloheximide inhibits SG induction in mutants with supernumerary SGs. Strains were grown at 30°C in synthetic defined media until exponential phase (OD_600_ = 0.5). Where indicated, Chx (100 µg/ml) was added to the culture and incubated 20 min before adding 2-DG to a final concentration of 400 mM. After 90 min of incubation with continued shaking, cells were fixed for 30 min at room temperature by adding formaldehyde to a final concentration of 4%, and cells were then washed twice with PBS. Quantification of SGs was done manually on fixed cells imaged in Axio Vision. 100–300 cells were counted in each sample.
(PDF)

Figure S7 Deregulated *STL1* expression in SG-defective mutants after hyperosmotic shock. A, B: For each experiment, mRNA levels were examined by quantitative RT-PCR and were expressed as a percentage from the maximum value reached by wt cells (indicated in each graph). *ACT1* was used as a reference gene. The data shown are the mean and standard deviation of at least three independent experiments. **A**) Induction of native *STL1* mRNA in wt and SG-defective mutant cells exposed to 0.4 M NaCl. **B**) Induction of *STL1*-LacZ mRNA in wt and SG-defective mutant cells exposed to 0.4 M NaCl. **C**) Induction of *STL1*-LacZ after hyperosmotic shock (0.4 M NaCl). A β-galactosidase assay was performed to determine the expression of the *STL1*-LacZ fusion from a 2 µm plasmid containing the *STL1* promoter fused to LacZ [7]. β-galactosidase activity was expressed in Miller units [73]. Wt and SG-defective mutants (*mft1Δ*, *arc1Δ*, *rps28bΔ*, *rpl20bΔ*, *top3Δ*) were examined, and in addition mutants with no SG phenotype (*ybl059wΔ, ybl060wΔ*) or with supernumerary SGs (*ccw12Δ*, *ynl198wΔ*). **D**) Expression of *STL1*-lacZ in mutants with SG defects (*mft1Δ*, *arc1Δ*, *top3Δ*) or with no SG phenotype (*ybl060wΔ*) after severe hyperosmotic shock (1.5 M NaCl).
(PDF)

Figure S8 Hog1 and eIF2α phosphorylation after stress in SG-defective mutants is not different from wt. Phosphorylation of Hog1 was used as an indicator of HOG pathway activity, and phosphorylation of eIF2α (Sui2) as an indicator of TOR pathway activity. Wt, *mft1Δ*, *arc1Δ*, and *top3Δ* were analyzed. Cells were exposed to hyperosmotic stress (0.4 M or 1.5 M NaCl) or heat stress (44°C) as indicated, and samples were recovered at times indicated in each panel. Protein extracts were obtained in the presence of phosphatase inhibitors and were analyzed by western blot using specific antibodies (see Materials and Methods). **A**) Phosphorylation of Hog1 during hyperosmotic stress in wt and SG-defective mutants. **B**) Phosphorylation of eIF2α (Sui2) during heat stress in SG-defective mutants. The *gcn2Δ* mutant was included as a negative control (Gcn2 is the eIF2α kinase).
(PDF)

Figure S9 Location of LCRs in proteins associated with SG defects. The locations of LCRs [32] within the 54 proteins associated with strongly SG-defective phenotypes are shown.
(PDF)

Table S1 Mutants with the most strongly deviating SG phenotype (increased or decreased) from the large-scale screen. SG phenotype relative values, as defined in Fig. 1 C legend, from the large-scale screen using 2-DG, are shown. SG phenotype values given for heat shock, 1.5 M KCl, and 1.5 M NaCl treatment are from screens directed at this set of mutants, using semi-automated analysis as for the large-scale screen. The presence of genes or gene products recovered in previously published related screens are shown in the respective columns: proteins found in SGs or PBs by mass spectrometry analysis [46], mutants forming SGs in the absence of stress [27], and homologous human genes identified as important for SG formation by RNA interference screening [14].
(XLSX)

Table S2 Mutants with manually confirmed SG phenotypes. Mutants from the large-scale screen (Table S1) with decreased (tab 1) or increased (tab 2) SG numbers were re-examined manually, using 2-DG as the stressor. The cases where the phenotype was confirmed were included in this table. In addition, some mutants were examined that did not appear from the large-scale screen; temperature-sensitive mutants (taken from a separate mutant collection) and deletion mutants with functions in certain functional categories as appeared in the large-scale screen, GSE/EGO complex and Ran ^GDP/GTP^ function. Presence of genes or gene products from related screens are shown as in Table S1.
(XLSX)

Table S3 Statistical significance P values for changes in heat-induced mutation rates in wt and mutant strains. The P values corresponding to the data in Fig. 4A are listed.
(DOCX)

Text S1 Supporting information reference.
(DOCX)
