# Supplementary material for: Stress Granule-Defective Mutants Deregulate Stress Responsive Transcripts
Source: PLoS Genet. 2014 Nov 6;10(11):e1004763. doi: 10.1371/journal.pgen.1004763 (PMC4222700; doi:10.1371/journal.pgen.1004763)
Supplement: Figure S1 — Co-localization of Hsp104 and Pab1 after different types of stress. Wild-type cells (his3) containing the genomic tags Pab1-RFP and Hsp104-GFP was grown at 30°C in synthetic defined media until exponential phase (OD600 = 0.5). Then, cells were allowed to grow to stationary phase overnight, exposed to heat shock (44°C, 45 min), osmotic stress (1.5 M NaCl or 1.5 M KCl, 90 min), or heat plus glucose starvation (400 mM 2-DG at 44°C, 45 min). Non-stressed cells were included as a control. After incubation with continued shaking, cells were fixed and imaged as described in Materials and Methods. (PDF) [file pgen.1004763.s001.pdf]

Supplementary Figure S1

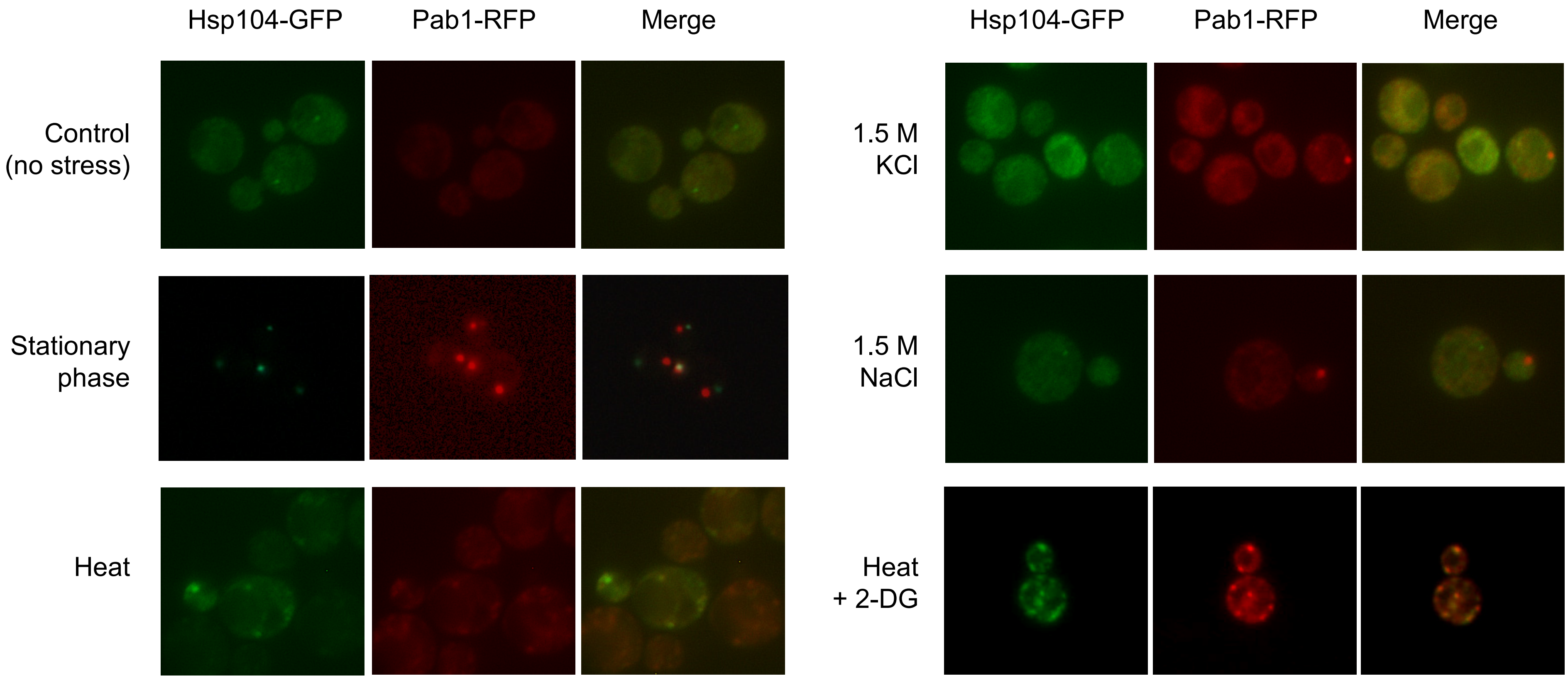

Co-localization of Hsp104 and Pab1 after different types of stress

Wild-type cells containing the genomic tags Pab1-RFP and Hsp104-GFP were grown at 30ºC in synthetic defined media until exponential phase (OD_{600} = 0.5). Then, cells were allowed to grow to stationary phase overnight, exposed to heat shock (44ºC, 45 min), osmotic stress (1.5 M NaCl or 1.5 M KCl, 90 min), or heat plus glucose starvation (400 mM 2-DG at 44ºC, 45 min). Non-stressed cells were included as a control. After incubation with continued shaking, cells were fixed and imaged as described in Materials and Methods.
