# Supplementary material for: Stress Granule-Defective Mutants Deregulate Stress Responsive Transcripts
Source: PLoS Genet. 2014 Nov 6;10(11):e1004763. doi: 10.1371/journal.pgen.1004763 (PMC4222700; doi:10.1371/journal.pgen.1004763)
Supplement: Figure S2 — Pab1 levels in SG-defective mutants. Wt (his3) or mutant cells expressing Pab1-RFP were grown at 30°C in synthetic defined media until exponential phase (OD600 = 0.5). Half of each culture was then either treated with 400 mM 2-DG for 90 min, or grown for the same time with addition of 2-DG. After western blot, protein extracts were probed by α-RFP antibodies (GenScript) and using α-Pgk1 antibody (Abcam) as a loading control. (PDF) [file pgen.1004763.s002.pdf]

Supplementary Figure S2

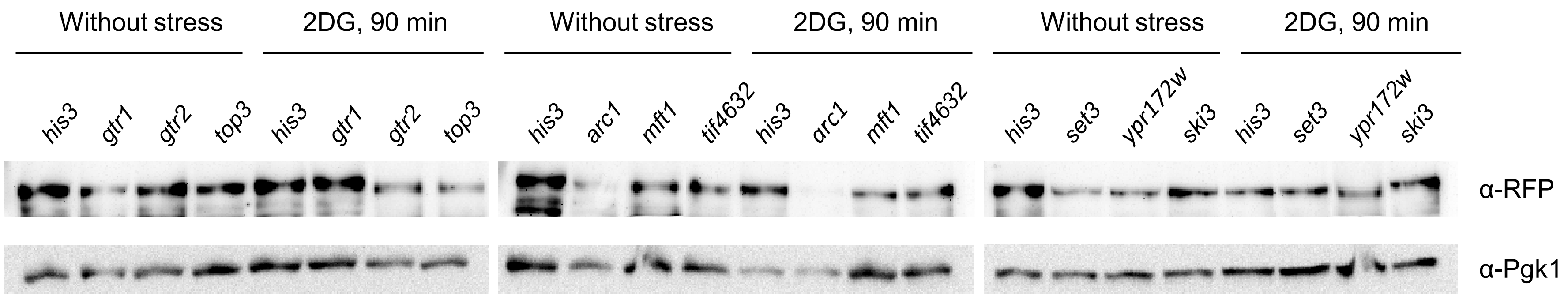

Pab1 levels in SG-defective mutants

Wt (his3) or mutant cells expressing Pab1-RFP were grown at 30ºC in synthetic defined media until exponential phase (OD_{600} = 0.5). Half of each culture was then either treated with 400 mM 2-DG for 90 min, or grown for the same time with addition of 2-DG. After western blot, protein extracts were probed by α-RFP antibodies (GenScript) and using α-Pgk1 antibody (Abcam) as a loading control.
