# Supplementary material for: Stress Granule-Defective Mutants Deregulate Stress Responsive Transcripts
Source: PLoS Genet. 2014 Nov 6;10(11):e1004763. doi: 10.1371/journal.pgen.1004763 (PMC4222700; doi:10.1371/journal.pgen.1004763)
Supplement: Figure S3 — Detection of Pbp1-positive SGs in mutants with SG defects or supernumerary SGs. Strains were transformed with a plasmid expressing Pbp1-mCherry (Text S1) and grown at 30°C in synthetic defined medium without uracil until exponential phase (OD600 = 0.5). Glucose was removed by washing the cells twice with medium without glucose and inoculating in the same medium, and samples were taken at the indicated times thereafter. For quantification of the fraction of Pbp1-positive SGs, unfixed live cells were imaged in Axio Vision and manual quantification of number of cells containing SG was performed (a total of 100–300 cells from were counted for each sample). Values represent two independent experiments; range indicates standard deviation. SG-Pab1 defective mutants: arc1Δ, top3Δ, hgh1Δ and vam3Δ. SG-Pab1 supernumerary mutants: brp1Δ, ccw12Δ, pep4Δ and ric1Δ. (PDF) [file pgen.1004763.s003.pdf]

Supplementary Figure S3

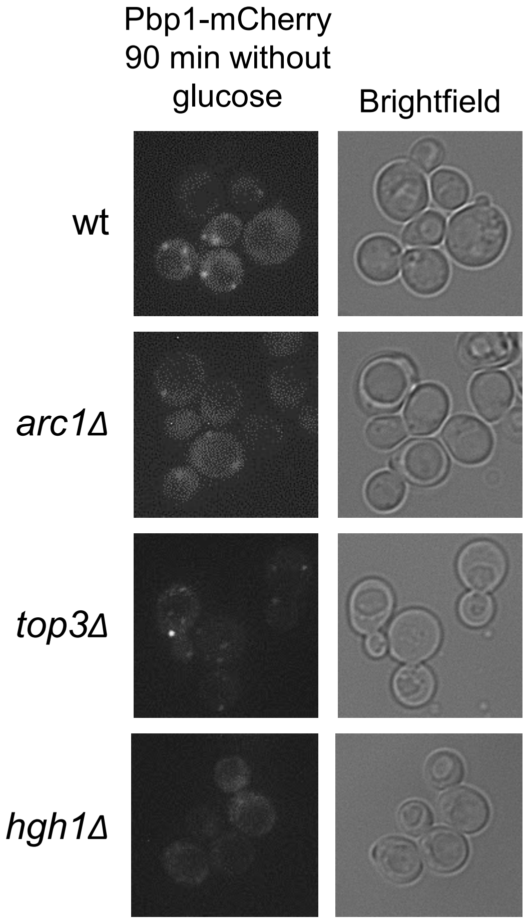

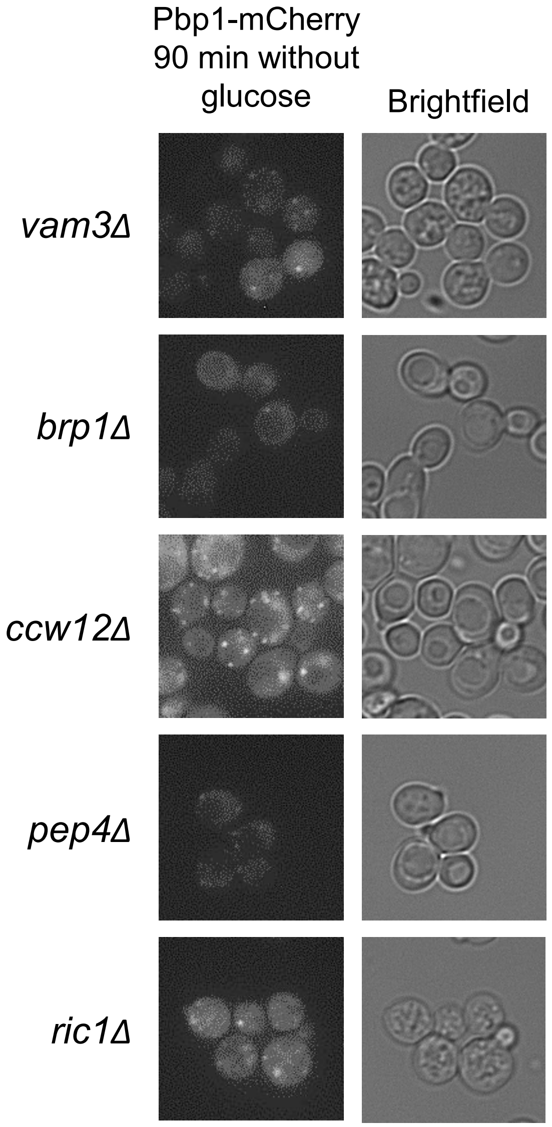

Detection of Pbp1-positive SGs in mutants with SG defects or supernumerary SGs

Strains were transformed with a plasmid expressing Pbp1-mCherry (Swisher 2010) and grown until exponential phase. Glucose was removed, and samples were taken at the indicated times thereafter. Unfixed live cells were imaged and manual quantification of number of cells containing SG was performed.
SG-Pab1 defective mutants: *arc1Δ*, *top3Δ*, *hgh1Δ,* and *vam3Δ*.
SG-Pab1 supernumerary mutants: *brp1Δ*, *ccw12Δ*, *pep4Δ* and *ric1Δ*.

Pbp1-mCherry (SD medium without glucose)			
	15 min	90 min	135 min
wt	15.2 ± 3.1	36.2 ± 4.8	37.3 ± 7.7
arc1	3.9 ± 1.0	30.1 ± 6.4	34.2 ± 9.1
top3	38.7 ± 9.9	50.4 ± 6.8	41.5 ± 7.2
hgh1	3.5 ± 4.3	22.1 ± 4.5	16.5 ± 3.0
vam3	9.6 ± 8.4	54.0 ± 8.2	54.8 ± 10.2
brp1	5.2 ± 4.4	16.7 ± 6.8	41.6 ± 6.3
ccw12	14.0 ± 14.7	61.5 ± 5.0	65.2 ± 14.5
pep4	12.6 ± 10.8	56.0 ± 20.6	59.3 ± 21.8
ric1	27.2 ± 16.9	59.3 ± 18.4	56.4 ± 7.7
