# Supplementary material for: Stress Granule-Defective Mutants Deregulate Stress Responsive Transcripts
Source: PLoS Genet. 2014 Nov 6;10(11):e1004763. doi: 10.1371/journal.pgen.1004763 (PMC4222700; doi:10.1371/journal.pgen.1004763)
Supplement: Figure S4 — SG phenotypes in different stresses. A: Mutants were selected that had>20% SG phenotype deviation from the wt in the large-scale screen in 2-DG, and where the difference was statistically significant (P<0.05). Cells were exposed to the indicated stress conditions: 400 mM 2-DG for 90 min; 44°C for 45 min; 1.5 M KCl for 90 min; or 1.5 M NaCl for 90 min, and the SG phenotype quantitated. B: Venn diagram showing how SG-defective phenotypes in different stress conditions are shared in individual mutants. (PDF) [file pgen.1004763.s004.pdf]

Supplementary Figure S4

A

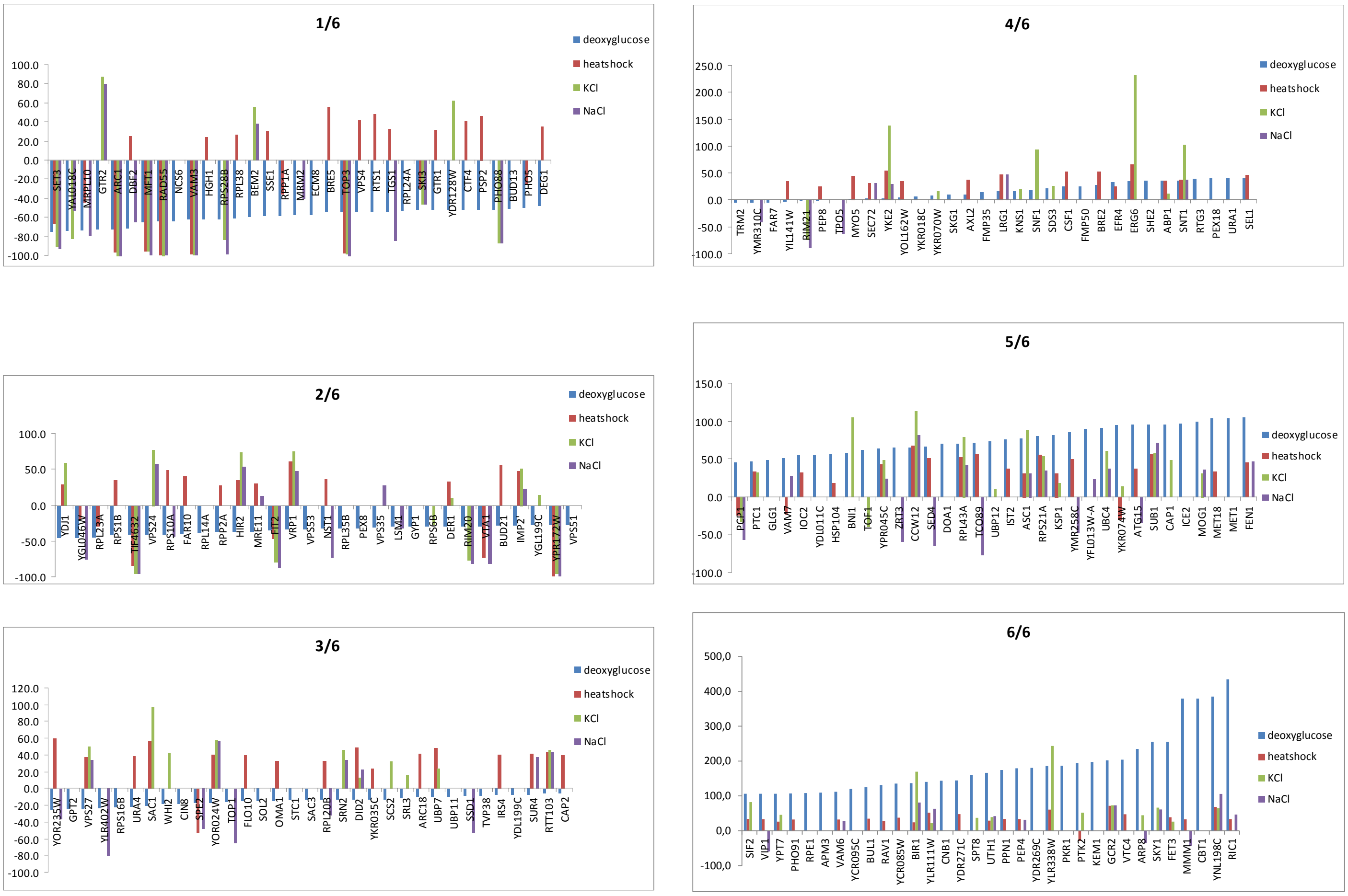

B

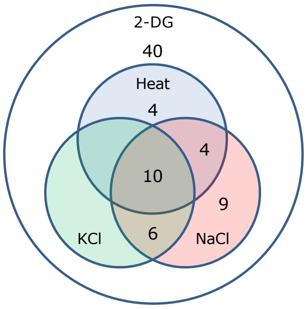

SG phenotypes in different stresses

A Mutants were selected that had > 20 % SG phenotype deviation from the wt in the large-scale screen in 2-DG, and where the difference was statistically significant (P < 0.05). Cells were exposed to the indicated stress conditions: 400 mM 2-DG for 90 min; 44oC for 45 min; 1.5 M KCl for 90 min; or 1.5 M NaCl for 90 min, and the SG phenotype quantitated

B Venn diagram showing how SG-defective phenotypes in different stress conditions are shared in individual mutants
