# Supplementary material for: Stress Granule-Defective Mutants Deregulate Stress Responsive Transcripts
Source: PLoS Genet. 2014 Nov 6;10(11):e1004763. doi: 10.1371/journal.pgen.1004763 (PMC4222700; doi:10.1371/journal.pgen.1004763)
Supplement: Figure S5 — Position in the ribosome of cRPs associated with strong SG defects. Red, Rpl20b; green, Rpl24a; blue, Rps10a. 60S subunit is shown in cyan; 40S subunit in beige. (PDF) [file pgen.1004763.s005.pdf]

Supplementary Figure S5

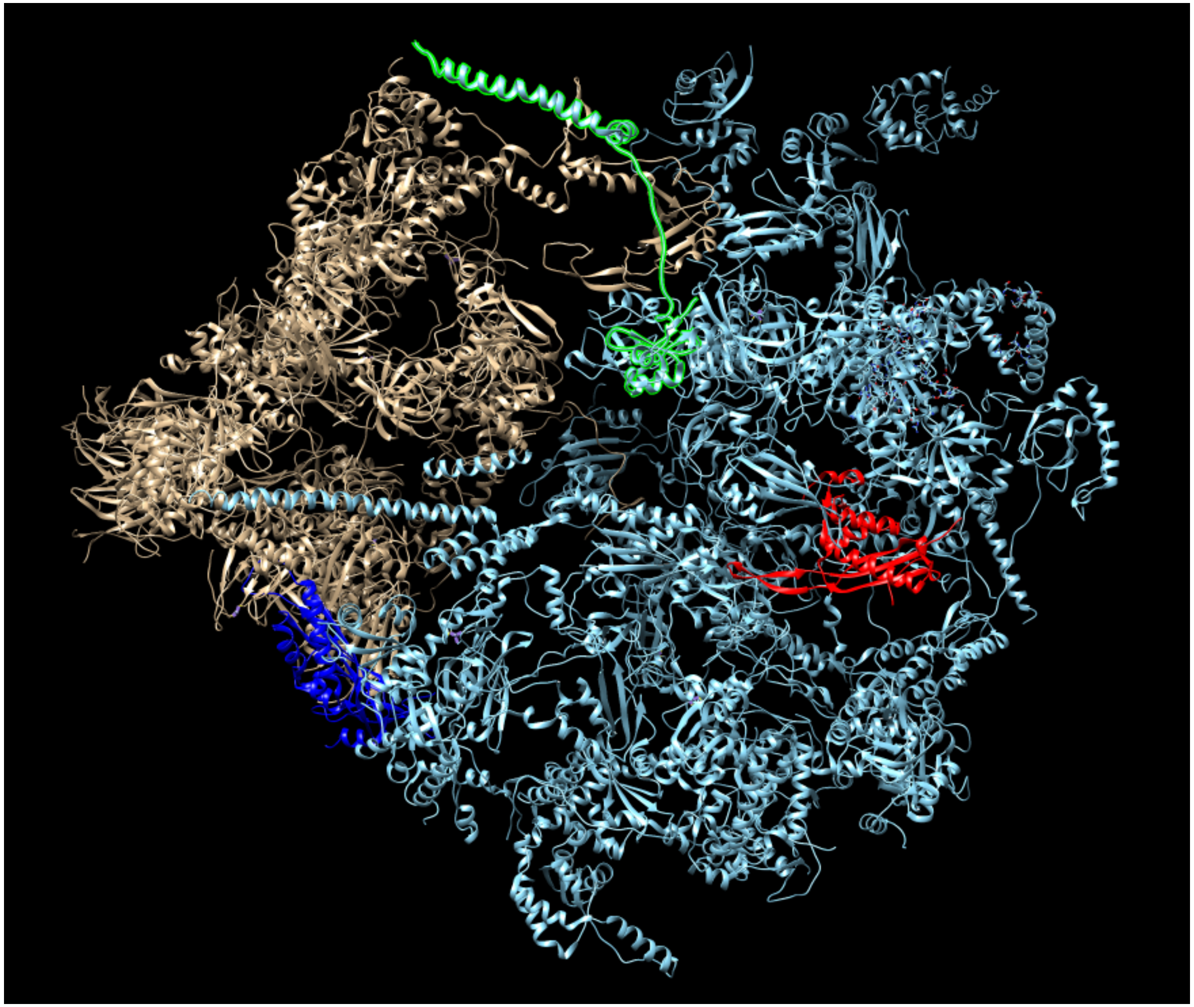

Position in the ribosome of cRPs associated with strong SG-defects

Red, Rpl20b; green, Rpl24a; blue, Rps10a. 60S subunit is shown in cyan; 40S subunit in beige.
