# Supplementary material for: Stress Granule-Defective Mutants Deregulate Stress Responsive Transcripts
Source: PLoS Genet. 2014 Nov 6;10(11):e1004763. doi: 10.1371/journal.pgen.1004763 (PMC4222700; doi:10.1371/journal.pgen.1004763)
Supplement: Figure S6 — Cycloheximide inhibits SG induction in mutants with supernumerary SGs. Strains were grown at 30°C in synthetic defined media until exponential phase (OD600 = 0.5). Where indicated, Chx (100 µg/ml) was added to the culture and incubated 20 min before adding 2-DG to a final concentration of 400 mM. After 90 min of incubation with continued shaking, cells were fixed for 30 min at room temperature by adding formaldehyde to a final concentration of 4%, and cells were then washed twice with PBS. Quantification of SGs was done manually on fixed cells imaged in Axio Vision. 100–300 cells were counted in each sample. (PDF) [file pgen.1004763.s006.pdf]

Supplementary Figure S6

	% cells with SGs	
Mutant	2DG	2DG+CHX
his3	23	2
brp1	71	2
ypr045c	63	0
atg15	43	3
ccw12	76	0
snt1	64	1
sub1	65	0

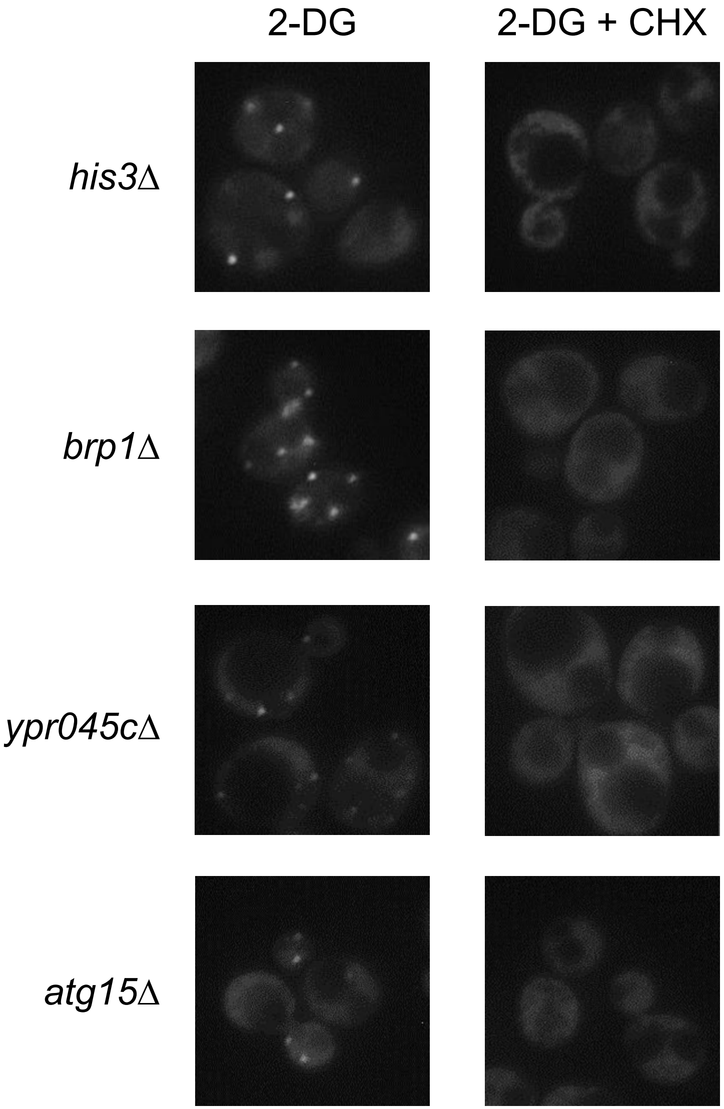

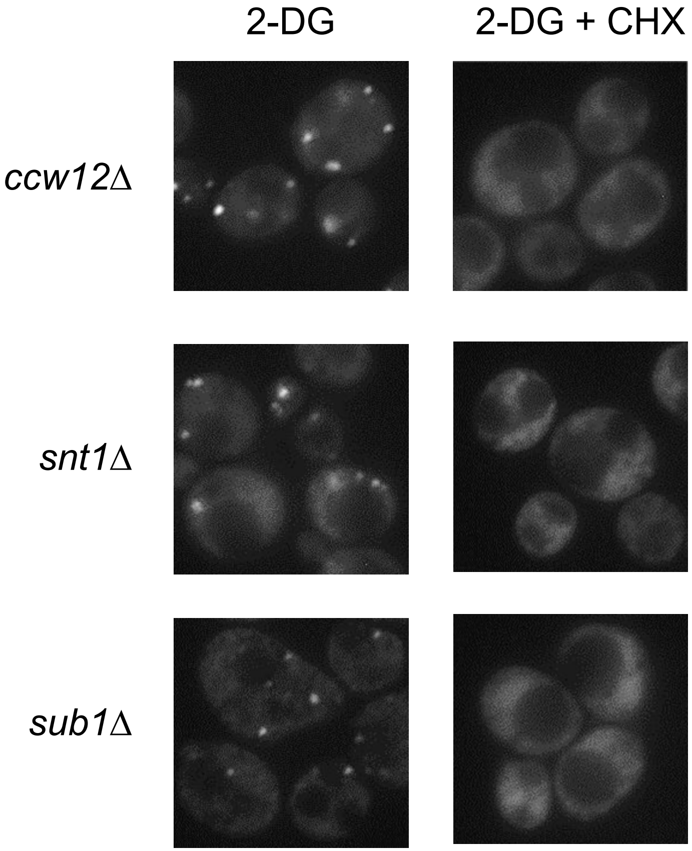

Cycloheximide inhibits SG induction in mutants with supernumerary SGs

Mutants with increased SG numbers in 2-DG (90 min) with or without pretreatment with cycloheximide
