# Supplementary material for: Stress Granule-Defective Mutants Deregulate Stress Responsive Transcripts
Source: PLoS Genet. 2014 Nov 6;10(11):e1004763. doi: 10.1371/journal.pgen.1004763 (PMC4222700; doi:10.1371/journal.pgen.1004763)
Supplement: Figure S7 — Deregulated STL1 expression in SG-defective mutants after hyperosmotic shock. A, B: For each experiment, mRNA levels were examined by quantitative RT-PCR and were expressed as a percentage from the maximum value reached by wt cells (indicated in each graph). ACT1 was used as a reference gene. The data shown are the mean and standard deviation of at least three independent experiments. A) Induction of native STL1 mRNA in wt and SG-defective mutant cells exposed to 0.4 M NaCl. B) Induction of STL1-LacZ mRNA in wt and SG-defective mutant cells exposed to 0.4 M NaCl. C) Induction of STL1-LacZ after hyperosmotic shock (0.4 M NaCl). A β-galactosidase assay was performed to determine the expression of the STL1-LacZ fusion from a 2 µm plasmid containing the STL1 promoter fused to LacZ [7]. β-galactosidase activity was expressed in Miller units [73]. Wt and SG-defective mutants (mft1Δ, arc1Δ, rps28bΔ, rpl20bΔ, top3Δ) were examined, and in addition mutants with no SG phenotype (ybl059wΔ, ybl060wΔ) or with supernumerary SGs (ccw12Δ, ynl198wΔ). D) Expression of STL1-lacZ in mutants with SG defects (mft1Δ, arc1Δ, top3Δ) or with no SG phenotype (ybl060wΔ) after severe hyperosmotic shock (1.5 M NaCl). (PDF) [file pgen.1004763.s007.pdf]

## Supplementary Figure S7

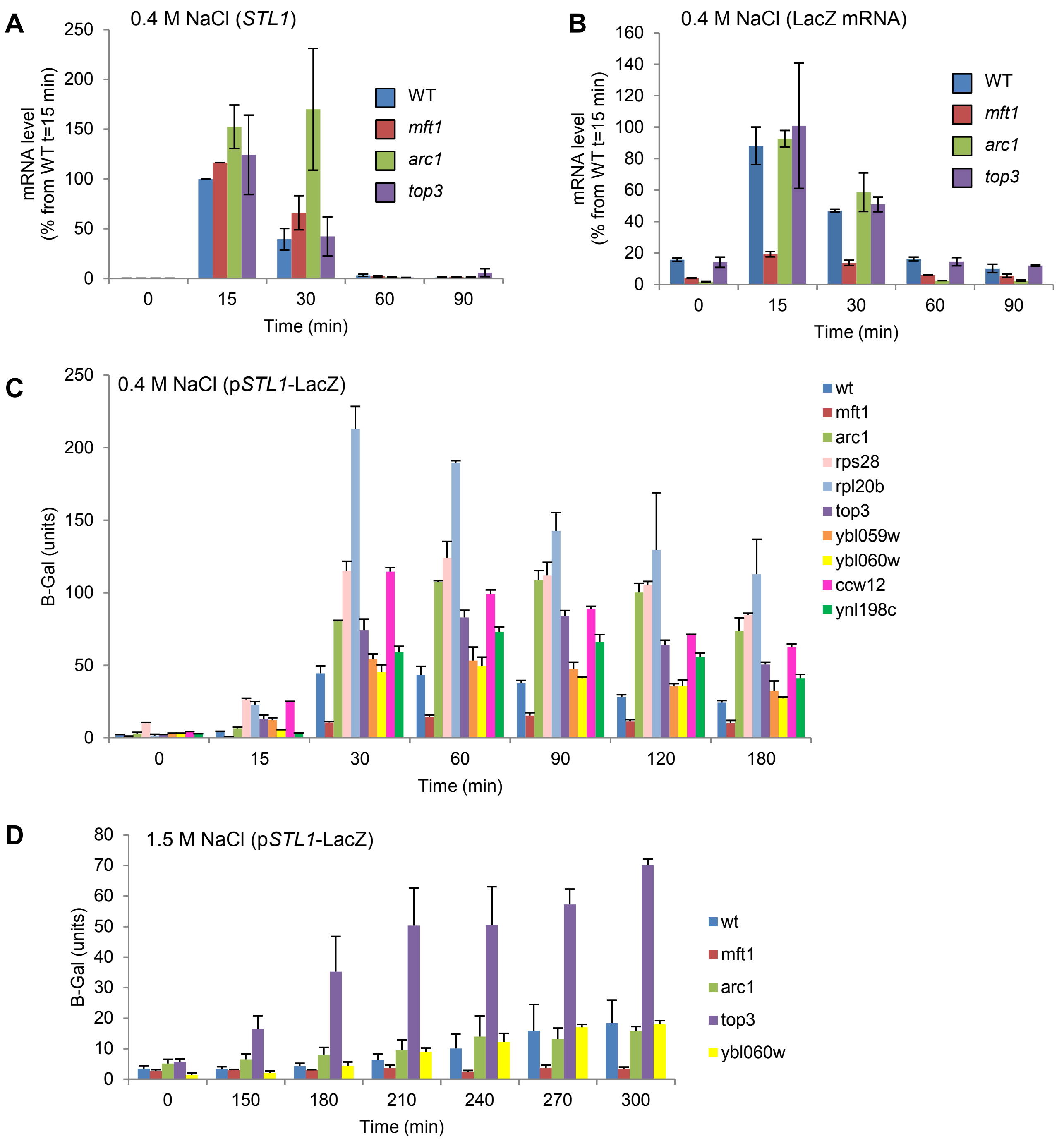


*Deregulated STL1 expression in SG-defective mutants after hyperosmotic shock*

**A**) Induction of native *STL1* mRNA in wt and SG-defective mutant cells exposed to 0.4 M NaCl.
**B**) Induction of *STL1*-LacZ mRNA in wt and SG-defective mutant cells exposed to 0.4 M NaCl.
**C**) Induction of *STL1*-LacZ after hyperosmotic shock (0.4 M NaCl). Wt and SG-defective mutants (*mft1Δ*, *arc1Δ*, *rps28bΔ*, *rpl20bΔ*, *top3Δ*) were examined, and in addition mutants with no SG phenotype (*ybl059wΔ, ybl060wΔ*) or with supernumerary SGs (*ccw12Δ*, *ynl198wΔ*).
**D**) Expression of *STL1*-lacZ in mutants with SG defects (*mft1Δ*, *arc1Δ*, *top3Δ*) or with no SG phenotype (*ybl060wΔ*) after severe hyperosmotic shock (1.5 M NaCl).
