# Supplementary material for: Stress Granule-Defective Mutants Deregulate Stress Responsive Transcripts
Source: PLoS Genet. 2014 Nov 6;10(11):e1004763. doi: 10.1371/journal.pgen.1004763 (PMC4222700; doi:10.1371/journal.pgen.1004763)
Supplement: Figure S8 — Hog1 and eIF2α phosphorylation after stress in SG-defective mutants is not different from wt. Phosphorylation of Hog1 was used as an indicator of HOG pathway activity, and phosphorylation of eIF2α (Sui2) as an indicator of TOR pathway activity. Wt, mft1Δ, arc1Δ, and top3Δ were analyzed. Cells were exposed to hyperosmotic stress (0.4 M or 1.5 M NaCl) or heat stress (44°C) as indicated, and samples were recovered at times indicated in each panel. Protein extracts were obtained in the presence of phosphatase inhibitors and were analyzed by western blot using specific antibodies (see Materials and Methods). A) Phosphorylation of Hog1 during hyperosmotic stress in wt and SG-defective mutants. B) Phosphorylation of eIF2α (Sui2) during heat stress in SG-defective mutants. The gcn2Δ mutant was included as a negative control (Gcn2 is the eIF2α kinase). (PDF) [file pgen.1004763.s008.pdf]

Supplementary Figure S8

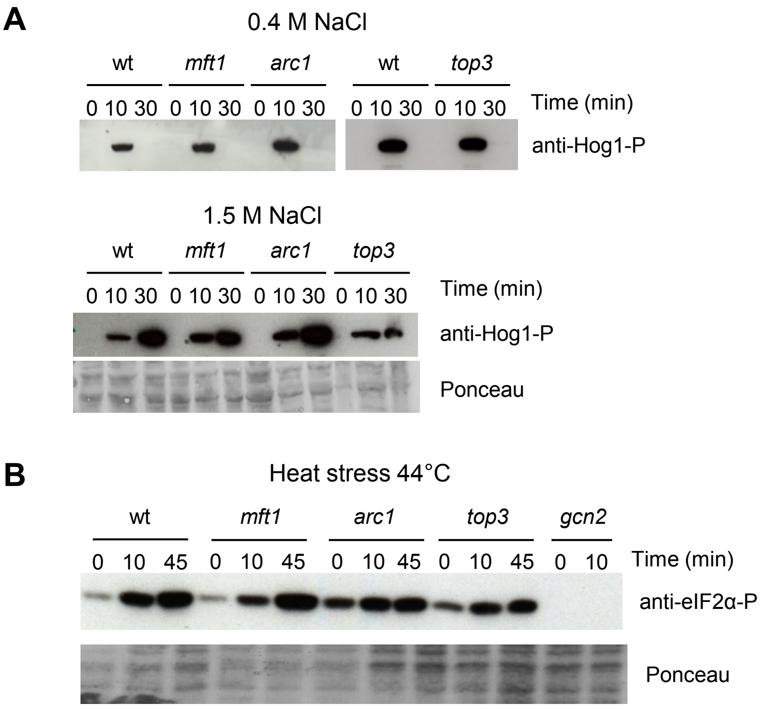

HOG and TOR signaling activity after stress, measured as Hog1 and eIF2α phosphorylation, respectively, in wt and SG-defective mutants

Wt, *mft1Δ*, *arc1Δ*, and *top3Δ* were analyzed. Cells were exposed to hyperosmotic stress (0.4 M or 1.5 M NaCl) or heat stress (44°C) as indicated, and samples were recovered at times indicated in each panel. Protein extracts were obtained in the presence of phosphatase inhibitors and were analyzed by western blot using specific antibodies (see Materials and Methods).

A) Phosphorylation of Hog1 during hyperosmotic stress in wt and SG-defective mutants

B) Phosphorylation of eIF2α (Sui2) during heat stress in SG-defective mutants. The *gcn2Δ* mutant was included as a negative control (Gcn2 is the eIF2α kinase).
