# Supplementary material for: Stress Granule-Defective Mutants Deregulate Stress Responsive Transcripts
Source: PLoS Genet. 2014 Nov 6;10(11):e1004763. doi: 10.1371/journal.pgen.1004763 (PMC4222700; doi:10.1371/journal.pgen.1004763)
Supplement: Figure S9 — Location of LCRs in proteins associated with SG defects. The locations of LCRs [32] within the 54 proteins associated with strongly SG-defective phenotypes are shown. (PDF) [file pgen.1004763.s009.pdf]

Supplementary Figure S9

Location of LCRs in proteins associated with SG defects

The locations of LCRs (identified according to Li and Kahveci, 2006), within the 54 proteins associated with strong SG-defective phenotypes are shown.

Graph Based Algorithm

Low complexity regions masked by 'X':

>YBL087C | RPL23A
MSGNGAQGTKFRISLGLPVGAIMNCADNSGARNLYIIAVKGSGSRLNRLPAASLGDMVMA
TVKKGKPELRKKVMPAIVVRQAKSWRRRDGVFLYFEDNAGVIANPKGEMKGSAITGPVGK
ECADLWPRVASNSGXXX

>YAL018C | LDS1
MSFTGSLALAGIGGLVYKFGGGQSYEKLPYVNIPFNQYLDKVYKKHFSKVMSRTXXXXXX
XXXXXXXXXXXMYPFKGFLEFNTNXXXXXXXXXXXXXXXLIMFALVSFVYWATITPMYTA
FLIVLGPIGLFIAIFHSFLQANVFTLLFMRLSHFNNHLVEVCLEKNGLEENLSEVKPIKY
YAPINSIXXXXXXXXXXXXXXXXXXXXXXXXXXXISFFPLIGPILFHILISPFITQIYFTK
VLRLQNFDNIQRRENIYLHAGQYASFGFLAGLIESVPILAGFAISTNTIGSVLFNLDHPM
VPENLVETQAEIEAAPQDINQQPNQ

>YDL081C | RPP1A (RPLA1)
MSTESALSYAALILADSEIEISSEKLLTLTNAANVPVENIWADIFAKALDGQNLKDLLVN
FSAGXXXXXXXXXXXXXXXXXEAEAEKEEEEAKEESDDDMGFGLFD

>YDL136W |RPL35B (SOS2)
MAGVKAYELRTKSKEQLASQLVDLKKELAELKVQKLSRPSLPKIKTVRKSIACVLTVINE
QQREAVRQLYKGXXXXXXXXXXXXXXXXXXXLTKFEASQVTEKQRKKQIAFPQRKYAIKA

>YDR076W | RAD55
MSLGIPLSQLIVESPKPLSSGITGLDEILNLGFQARSIYEIFGPPGIGKTNFXXXXXXXX
XXXXXXXXINDDKILWIETFQEMPINILRERFQKFKIVEENVKRVRITKFGQLLYFFQNL
FKLSQSVRYKLVIIDGFSQLVCDHLCTLSKRXXXMIDKTIHELKCRHLILIFTVMTKYTH
STGSTIIVLNDCMNTAFQSNEFESLEEYYEILDDGSNFFVNSNNERRKNNVHILKSALVA
NIAMGSKDSTWEVFLRDRIGLFRDWNEQVDETVFVKSKRVKASSSQSNEGCTTIKEMRIN
KRNFENLRIAIVFNLHGEDRKREGRNLKRSRSSDDRNYIVKFDFDKATGQLRDIIDLKPD
TANIASFPTLSTSSSSCSQVFNNIDSNDNPLPNAEGKEEIIYDSEG

>YLR181C | VTA1
MASNAARVVATAKDFDKVGLGIIGYYLQLYAVELILSEEDRSQEMTALATELLDTIEAFK
KEIGGESEAEDSDKSLHVMNTLIHDQEKAKIYMLNFTMSLYNEKLKQLKDGPWDVMLKRS
LWXXXXXXXXXXXXXXXNISETSTNSLQKRIKYCKIYLSKLAKGEIGSSDEKTLDYADFA
DDSEEIKXXXXXXXXXXXXXXXXDKVEGLAPKDQTTSYEPVXXXXXXXXXXXXXXXXXQT
VDKNEDAITKDEQQVVKKEVDLTRPSAPSEPAAAEHKSYXXXXXXXXXXXXXXXXXIEQIQKL
AKYAISALNYEXXXXXXXXXXXXXXXXNSI

>YML062C | MFT1 (MFT52)
MPLSQKQIDQVRTKVHYSEVDTPFNKYLDILGKVTKLTGSIINGTLSNDDSKIEKLTEQN
ISQLKESAHLRFLDLQSSIDTKKVADENWETCQQETLAKLENLKDKLPDIKSIHSKLLLR
IGKLQGLYDSVQVINREVEGLSEGRTSLVVTRAEWEKELGTDLVKFLIEKNYLKLVDPGL
KKDSSEERYRIYDDFSKGPKELESINASMKSDIENVRQEVSSYKEKWLRDAEIFGKITSI
FKEELLKRDGLLNEAEGDNXXXXXXXXXXXXXXXXFKRQRSMVEVNTIENVDEKEESDHE
YDDQEDEENEEEDDMEVDVEDIKEDNEVDGESSQQEDNSRQGNNXXXXXXXXXXXXXXXX
XXXXXXXXXXXXXXXXXXXXXFSAXXXVEEVK

>YML063W | RPS1B (PLC2, RP10B)
MAVXXXXXXXXXXXXXXXXVVDPFTRKEWFDIKAPSTFENRNVGKTLVNKSTGLKNASDA
LKGRVVEVCLADLQGSEDHSFRKVKLRVDEVQGKNLLTNFHGMDFTTDKLRSMVRKWQTL
IEANVTVKTSDDYVLRIFAIAFTRKQANQVKRHSYXXXXXXXXXXKVISEILTREVQNST
LAQLTSKLIPEVINKEIENATKDIFPLQNIHVRKVKLLKQPKFDVGALMALHXXXXXXXX
XXXXXXFKDEVLETV

>YML121W | GTR1
MSSNNXXXXXXXXXXXXXKSSMRSIIFSNYSAFDTRRLGATIDVEHSHLRFLGNMTLNLW
DCGGQDVFMENYFTKQKDHIFQMVQVLIHVFDVESTEVLKDIEIFXXXXXXXXXXXXXXX
XFVLLHKMDLVQLDKREELFQIMMKNXXXXXXXXXXXXXXXXXTSIWDESLYKAWSQIVC
SLIPNMSNHQSNLKKFKEIMNALEIILFERTTFLVICSSNGXXXXXXXXXXXXXXVLLDP
KRFEKISNIMKNFKQSCTKLKSGFKTLILXXXXXXXXLSSNMVCFIVLKDMNIPQELVLE
NIKKAKEFFQ

>YML017W | PSP2 (MRS15)
MGTNNTSNNNGTTKKMSLEEFLGNDTLGESVWDEEDINLDAISNTTNIDILKQTKAGEHQ
RDGHQQHPHGGHGPMNRSRFSNAGPFGGGSMGDFANHHHPLQHQQGXXXXXXXXXXXXXX
XXXXXXXXXXXXXTKFIKFKLFWEINKNPSISTLKSGSIFDQNFKRDSKVAFVELYTSRD
MDKILNYWTTPLKEIYHITTAPAEFEDFKDYSTKVKLLTDPKDDAGKPFITKTQRSKSNP
FGSAKPVDTQSKILDIEEKMENLHVEDTTTLRASLIPSSDSMATTATGSKITILKKQTPT
EEESHSATPTPKPLSYSEVVERSVVNETSKKGTPLSKLDSPALELQSKPDKSDEFKGGDE
QGFEKGGDDKAQLDVSNDKDKGSETDVDKQFTFKNVEXXXXXXXXXXXXXXXXXXXXFXX
XXXXXXXXXXXXXXXXXXXXXXXXXXXXXSSYXXXXXXXXXXXXXXXXXXXXXXXXXXXX
XXXXXXXXLTSDSSLDASGNKKNDFTNSTSNTQQYSIFKPASGFLGQGNNDSIRNNGRGN
YNSSGMNGGSRGRGFGRGRGFXXXXXXXXXXXXXXXXXXXXXXXNNRTTDMPL

>YNL091W | NST1
MPPNXXXXXXXXXXXXXXXXXGNSDXXXXXXXXXXXXXXXXXLYHTESDYPTSRVIKRAP
NGDVIVEPINXXXXXXXXXXXXXXXXXXXXXXXXXXXXXXXXXESLSPEEKKTILRIEKE
EVFNVIRNYQDDHSCSCSVCGRRXXXXXXXXXXXXXXXXXXXXXXXPETNPIKFHLGIIK
ELQISKNQQQNDLSSTKGEVVKNFLXXXXXXXXXXXXXXXXXXXXXXXXXAHNETADNTS
LLEENLNNIHINKTSSEISANFNSVSDEELQQKYSNFTKTFISSHPKIAEEYVQKMMMYP
NIRALTDDLMNSNGQGFLNAIEDFVRDGQIQASKKDDSITEDEASSTDLTDPKEFTTMLH
SGKPLTEDEYADLQRNIAERMTNAYDTASKKFKDVSQLEKELFTRFMSGRDKKSFRELII
QSFKNKFDGELGPSVLAATLSSCFSSQSKDTXXXXXXXXXXXXXXXXXXXXXXXXXXXXX
XXXEGIEAVEKPEHDEKSNGIRETLHLSYDHDHKRQNHPHHHYXXXXXXXXXXXXXXXYI
SDIELPHDPHKHFHRXXXXXXXXXXXXXXXXXXXXXXXXXTYDSGLDETDRLEEGRKLIQ
IAITKLLQSRIMASYHXXXXXXXXXXXXXXXXXXXRXXXXXXXXXXXXXXXXXXXXXXXX
XXXXXXXXKRXXXXXXXXXXXXXXXXXXXXAXXXXXXXXXXXXXXXXXXRLXXXXXXXXX
XXXXXXXXXXLXXXXXXXXXXXXXXXXXXXXXXXXEKEEEERQRLIAEDALRKQKLNEEQ
TSANILSAKPFTENGVGNPVSSQSHPNMTNYQEDNSCSINDEILKMVNSVAASKPVSPTG
FNVHDLLLPSTNNQMPAMEQSHLPQPGNQNNHFGTTTIPNALDLATKSSLQTENNYLMNS
QTLENTSLLMHNNSSPTKLLPNDFGLSSWGGLTNTMSINPTCKPPVIQTSEMESQAHKSS
PQATMPSFGLPNGGTHRKSFTDELNTLTSMLXXXXXXXXXXXXXXXPPSQRSVWNDQKSS
FSGPSTAGNFNNSSIQSGMLLAPTLGSVESFPNRTSIWDSSTTPMMNKSELSGRNITSTA
QDSPAFMASNIWSSNSQYNSPYLTSNVLQSPQISSGVDESHILDSIYNTYLAISPQDSLN
PYIAIGTLFQNLVGLNLDYSTFINKLISMQGAYNCEFFTDNNGSITHVRFARQTPAGHSK
GLLNQLFSGLNDPTATPFTSRPHTSTRASFPIASSTTQTS

>YNR050C | LYS9 (LYS13)
MGKNVLLLGSGFVAQPVIDTLAANDDINVTVACRTLANAQALAKPSGSKAISLDVTDDSA
LDKVLADNDVVISLIPYTFHPNVVKSAIRTKTDVVTSSYISPALXXXXXXXXXXXXXXXX

XXGLDPGIDHLYAVKTIDEVHRAGGKLKSFLSYCGGLPAPEDSDNPLGYKFSWSSRGVLL
ALRNSAKYWKDGKIETVSSEDLMATAKPYFIYPGYAFVCYPNRDSTLFKDLYHIPEAETV
IRGTLRYQGFPEFVKALVDMGMXXXXXXXXXXXXXXXXXALKQYLGAKSTSKEDLIASID
SKATWKDDEDRERILSGFAWLGLFSDAKITPRGNALDTLCARLEELMQYEDNERDMVVLQ
HKFGIEWADGTTETRTSTLVDYGKVGGYSSMAATVGYPVAIATKFVLDGTIKGPGLLAPY
SPEINDPIMKELKDKYGIYLKEKTVA

>YNR051C | BRE5
MGVTVQDICFAFLQNYYERMRTDPSKLAYFYASTAELTHTNYQSKSTNEKDDVLPTVKVT
GRENINKFFSRNDAKVRSLKLKLDTIDFQYTGHLHKSILIMATGEMFWTGTPVYKFCQTF
ILLPSSNGSTFDITNDIIRFISNSFKPYVLTDASLSQSNEENSVSAVEEDKIRHESGVEK
EKEKEKSPEISKPKAKKETVKDTTAPTESSTQEKPIVDHSQPRAIPVTKESKIHTETVPS
STKGNHKQDEVSTEELGNVTKLNEKSHKAEKKAAPIKTKEGSVEAINAVNNSSLPNGKEV
SDEKPVPGGVKEAETEIKPIEPQVSDAKESGNNASTPSSSPEPVANPPKMTWASKLMNEN
SDRISKNNTTVEYIRPETLPKKPTERKFEMGNRRDNASANSKNKKKPVFSTVNKDGFYPI
YIRGTNGLREEKLRSALEKEFGKVMRITAADNFAVVXXXXXXXXXXXXXXXXXXIDGIEV
CLERKTVKKPTSNNPPGIFTNGTRSHRKQPLKRKD

>YOL039W | RPP2A (RPL44, RPLA2)
MKYLAAYLLLNAAGNTPDATKIKAILESVGIEIEDEKVSSVLSALEGKSVDELITEGNEK
LAAVPAXXXXXXXXXXXXXXXXXXXXXXXXXXXXXXXXXMGFGLFD

>YOR106W | VAM3 (PTH1)
MSFFDIEAQSSKGNSQQEPQFSTNQKTKELSNLIETFAEQSRVXXXXXXXXXXXXXXXXL
RYKIETELIPNCTSVRDKIESNILIHQNGKLSADFKNLKTKYQSLQQSYNQXXXXXXXLKT
PISPGTSKERKDIHPRTEAVRQDPESSYISIKVNEQSPLLHNEGXXXXXXXXXXXXXXXG
LSXXXXXXXXXXXXXRSQQIGRIHTAVQEVNAIFHQLGSLVKXXXXXXVTTIDENISHLHD
NMQNANKQLTRADQHQRDRNKCGKVTLXXXXXXXXXXXXXXXS

>YOR014W | RTS1 (SCS1)
MMRGFKQRLIKKTTGXXXXXXXXXXXXXXXXXXXXXXXXXXXXXXXXXXXXXXXXHSSAS
STGSKSTTEKGKQSGSVPSQGKHHXXXXXXXXXXXXXXXXXXXXRXXXXXXXXXXXXXXX
XXRKGXXXXXXXXXXXXXXXXGXXXXXAAIMNPTPVLTVTKDDKSTSGEDHAHPTLLGAV
SAVPSSPISNASGTAVSSDVENGNSNNNNMNINTSNTQDANHASSQSIDIPRSSHSFERL
PTPTKLNPDTDLELIKTPQRHSSSRFEPSRYTPLTKLPNFNEVSPEERIPLFIAKVDQCN
TMXXXXXXXXXXXXXXXXXXXXLDELIEFLVTNRFTYTNEMYAHVVNMFKINLFRPIPPPV
NPVGDIYDPDEDEPVNELAWPHMQAVYEFFLRFVESPDFNHQIAKQYIDQDFILKLLELF
DSEDIRERDCLKTTLHRIYGKFLSLRSFIRRSMNNIFLQFIYETEKFNGVAELLEILGSI
INGFALPLKEEHKVFLVRILIPLHKVRCLSLYHPQLAYCIVQFLEKDPLLTEEVVMGLLR
YWPKINSTKXXXXXXXXXXXXXXXXXXXXXXXXXXXFVQLAKCISSPHFQVAEKVLSYWN
NEYFLNLCIENAEVILPIIFPALYELTSQLELDTANGEDSISDPYMLVEQAINSGSWNRA
IHAMAFKALKIFLETNPVXXXXXXXXXXXXXXXXXXXXXXXXEENWSKLEEYVKNLRINND
KDQYTIKNPELRNSFNTASENNTLNEENENDCDSEIQ

>YOR038C | HIR2 (SPT1)
MRLLKYPLDIHNEQVNALAALGPYIILAGSGGHVMAWRQQQLVDTAFDRVMIKDLKPEVS
FQVDQDTXXXXXXXXXXLETLYIGSEHRLWGYSGWLCRDTNNINSVEKMNSKLLFECKSP
STITDVKYDINLGILFVLLSNENKILLFRHKTXXXXXXXXXXXASKPITGIIDPTGQTFT
VMTSDRSILVYQINKTGTHKLINKLTQHVQMYPLHYRISMSPQADILPVINSVKGVPNNA
TSCTALLDRXXXXXXXXXXXXXPSSNGCRVLVYSPAFYEKPNLKKGXXXXXXXXXXXXXXD
GTILVWNTKRMKPLFNALQVSSTAINDMSWSQDGXXXXXXXXXXXXXXXXFQEKDLGVAL
PQTXXXXXXXXXXXXXXXXXXXLAEQIPKSFPENIKLEESASAAPIPNDIGRSAVGXXXXX
XXXXXXXXXXXXXIQSTSMEFNTPSYTVPRDLKRKPKEATPSNXXXXXXXXXXXELQPIDF

LDTGLLLPNTSFSRIRLATPKIRSTFKYSPINNPNLILDVKNGSGNEQRPTXXXXXXXXX
XXXXVLFQDFIPKLITICTAGDTFWSFCSEDGSIYIYSDSGRKLMAPLVLGVSISFLEAC
GTYLLCLTSIGELYCWNIEQKKLAFPTNTIYPLLNPSLRYSDDILTRAENXXXXXXXXXX
XXXXXXXNGDGYLFDKNMETWLLVSDGWWAYGSQYWDTTNTTGLXXXKANTDSFNGSESN
INEIVSDIKNDNQSIINFLECKTNDELNRKGRIKNLQRFARTILMKEGFENMEEIVTLSH
XXXXXXXXXXXXXPEEFSKLMMVYCIRLSELGYMDRLNDVFQWLYDDLPISGTGSAFADK
DFKRNLLKKILIACGDIRQVQRVTTRYAKEMNIIS

>YOR078W | BUD21 (UTP16, YOR29-29)
MSNGHVKFDADESQASASAVTDRQDDVLVISKKDKEVHSSSDEESDDDDAPQEEGLHSGK
SEVESQITQREEAIRLEQSQLRSKRRKQNELYAKQKKSVNETEVTDEVIAELPEELLKNI
DQKDEGSTQYSSSRHVTFDKLDESDENEEALAKAIKTXXXXXXXXXXXXXXXXGKFRVQL
LSTTQDSKTLPPKKESSIIRSKDRWLNRKALNKG

>YOR382W | FIT2
MKFSTIFGATTVMTAVSAAAVSSVMTTKTITATNGNNVYTKVVTDTADPIISYSTTRTVV
VSNSDATYTKVVTEGPDTTSEKSTTKTLTLTNXXXXXXXXXXXXXXXXXXXXXXXXXXXX
XXXXXXXXXXXXXXXXXXXXXXXLALGLISYLL

>YOR235W | IRC13
MGLYRPSKFFHPPIPHIPFTINPDFFSFHIQRLXXXXXXXXXXXXXXXXDIYKGFVFCCQ
LDLVHLFSYVFFLFLLKICVDVLQYVIYPKHFTHKKPGFENYSI

>YOR293W | RPS10A
MLMPKEDRNKIHQYLFQEGXXXAKKDFNQAKHEEIDTKNLYVIKALQSLTSKGYVKTQFS
WQYYYYTLTEEGVEYLREYLNLPEHIVPGTYIQERNPTQRPQRRY

>YPL157W | TGS1
MGRTFIXXXXXXXXXXXXXXHSNFRTLIKLLNNDAYKIESSKPLKNGKLFKYWKNXXXLF
SKIDSASIYMTDELWFSVTPERXXXXXXXXXXXXMPNAERILDVFCGGGGNTIQFAMQFP
YVYGVDYSIEHIYCTAKNAQSYGVDDRIWLKRGSWKKLVSKQKLSKIKYDCVFGSPPWGG
PEYLRNDVYDLEQHLKPMGITKMLKSFLKLSPNVIMFLPRNSDLNQLSRATRKVLGPFAK
CKVLYVKENGYMKGIFCMWGECFFNYEPASTENSRRXXXXXXXXXXXXXXLXXXXXXXXX
XXXKDNTVDIYDVNG

>YPR172W | ORF, uncharacterized
MAWTSTLPAHLLNLIKNSKYVHVATCSKDCIPSVALMNYIYVPGEKLFGQTDNKNDYIIF
VSPQDTQKFYNIKENPKVALLFHDWIIANNLSVGKESISGXXXXXXXXXXXXXXXXXXXX
XXXXXXXXXXXMSASIGGETEIVNPESEESKYYKDLILKANPDAKAFIFEKNTAVVKVRI
DNARVSNNENRTMFLSKGKS

>YFL001W | DEG1 (PUS3, HRM3)
MSNFIRRLVGKMKAISTGTNAIVSKKDSIYANWSKEQLIRRITELENAXXXXXXXXXXXXX
XXXXXXXXXXXXXXXXXXXXPKXXXXXXXXXXXXXXXXAYLGWNYNGLAVQKEYTPLPTV
EGTILEAMNKCKLVPSMVLQDYKFSRCGRTDKGVSAMNQVISLEVRSNLTDEEQRDPTND
SREIPYVHVLNQLLPDDIRISAVCLRPPPNFDARFSCVHRHYKYIFNGKNLNIEKMSKAA
SYFVGERDFRNFCKLDGSKQITNFKRTIISSKILPLSETFYCFDLVGSAFLWHQVRCMMA
ILFLVGQSLEVPEIVLRLTDIEKTPQRPVYEMANDIPLLLYDCKFPEMDWQEPTVDDYKA
IKFTTATEALTLHYELKAAVCNIFKDVLPTANTNNFSKTIINLGDGRGKVVGTYVKLEDR
SXXXXXXXXXXXXXXXXXNNKNK

>YNL284C | MRPL10
MKAERQTGLRNSFTTVIGRKLINTFVPSMMLTSVAGNDIFFRGLFKSPVLAFQSYRYVSI
LGQLKPSDGSTKSFKRLXXXXXXXXXXXXXXXXXXXXXXXXXXXXXXXXXTPIYKLFPKI
GFTNVGAKPXXXXXXXXXXWFHDKNRLHLQPGEVLDMNKMRKLGLVTGPIKYGVKILASG
KFHYNLPIALEXXXXXXXXXXXXXXXGGKFTARYYTPLGLRAHLNPQWFLEXXXXXXXXX
XXXXXXXXXXXXXXXXXGYLVMEKDKLLQDXXXXXXXXXXXXXXXXVKKSSLEIELEELS
PEKDWVPVVSNSKVMNIKALDH

>YLR238W | FAR10
MTGPGPEINKEEHPSSPGKKQITYXXXXXXXXXXXXXXXXXXXXXXXXXXXIADPTKSYF
PHSISRTPRRKYTYILVLTSLNGTFESKHVVIPFKPDGLKLGRPVANSNXXXXXXLRGGK
RVDSHTFSQVRSDNGNFDSRVLSRNHALLSCDPLTGKVYIRDLKSSNGTFINGQRIGSND
VEIKVGDVIDLGTDIDTKIEHRKISATVEELFVQPLLESPIFENEDSDDCHTITEKEEAA
AITSHIYGDSNNLELEEVILGSDTEILSGIFINNCIGTSPXXXXXXXXXXXXXXXXXXXX
XXXXXXXXXLINYTTHLEYTNKLLVEKNDQQLVKLQNGLRRKLSGKYEKIIEQNRNQVKQ
LERDHMFFKKSFEVKKRRNNEKQKSMEREIEDLKTRLEVERYKNSQMMKKNKQKEQELST
ASKKKTTEHDTRGVPGMNPKGTDKFSIKNTLCNHFTLLTFGTISIGIIAIVFKILSPN

>YBR201W | DER1
MDAVILNLLGDIPLVTRLWTIGCLVLSGLTSLRIVDPGKVVXXXXXXXXXXXXXXXXXXI
FDYGAFNWISMINIFVSANHLSTLENSFNLRRKFCWIIFLLLVILVKMTSIEQPAASLGV
LLHENLVYYELKKNGNQMNVRFFGAIDVSPSIFPIYMNAVMYFVYKRSWLEIAMNFMPGH
VIYYMDDIIGKIYGIDLCKSPYDWFRNTETP

>YBR076W | ECM8
MDYGYFFPAQRIEETNGVDFWIDSNAEFTQSKRPDSSTSTLSRVLTDTTNVSNNSGSLKR
KTIKNKIFPQRKIFNDSENFDFGKANTDCKHVFKSISKQLIFLPRCFQHHSIRGWMKDRY
SEFGYKIKRNQNCPPXXXXXXXXXXXXXXXXEESNPNXXXXXXXXXXXXXXXXXELMCRF
CQGNNWILAENYLKHLFFAHGILSEFKPHTLYHFESKLLKIQGKLNFKIQVLKEPEFSKK
ILNSLTVSIIPSPLAYYTQTLNGGFRRIHVKCPHCENWIRLGWCEYDEIIRDSFQDFESL
RNLNADYNGMSYIQTRNREDIEGIYENYFTHYIQCDLATFRTKCLYVQVITKSN

>YBR106W | PHO88
MNPQVSNIIIMLVMMQLSRRIDMEDPTIIMYIRILYCSSIGISWIIYQMARKRIVAKNDM
TTMKYVEPGNAMSGEGEKLQVTTVRDYDLKEIDSAIKSIYTGMAMMGXXXXXXXXXXXXX
XXSISPVKSALEHNEVKIHLFGKPATGDLKRPFKAPSLFGGMGQTGPKTDKKSIEEAERA
GNAGVKAE

>YGL174W | BUD13 (CWC26)
MALHQYLSETYGPTKPKNKTXXXXXXXXXXXXXXXXXXXXXRLSTLQXXXXXXXXXXXX
XXXXXXXXNIWKNLETNELSHAITHPSASSITGNESKNDLKEIRAQEPLVTVADKSKTRK
TIYRDAQGHKIQEDSKIDDSSFSRSKYEDEKAAEREQYLKNLNMGDVQKLGINVDAHDXX
XNQTASSLTIEDPAITFTHXXXXXXXXXXXXXXXXXXPAPENRFAIMPGSRWDGVHRSNG
FEEKWFAKQNEINEKKVQSYTLQEDY

>YGL199C | ORF, dubious
MEMYEISDTLWCGLVSLRCNAITSKMNQLDTNRLENSDRACDTNREYNMINARGAHRSQL
AEMSLSAAPIPSSEGLKNEGKCGYTTCFSLELISTTSRFSVXXXXXXXXXXXXXXXXXXP
MLSATPPEAAKGASGSMKSGSIVVSSLPYDILPALL

>YGL031C | RPL24A (RPL30A)
MKVEIDSFSGAKIYPGRGTLFVRGDSKIFRFQNSKSASLFKQRKNPRRIAWTVLFRKHHK
KGITEEVAKKRSRKTVKAQRPITGASLDLIKERRSLKPEVXXXXXXXXXXXXXXXXXXXX
XXXXXXXXXXXXXXXXXXXXQQAKGAFQKVAATSR

>YLR234W | TOP3 (EDR1)
MKVLCVAEKNSIAKAVSQILXXXRSTSRDSGYMYVKNYDFMFSGFPFARNGANCEVTMTS
VXXXXXXXXXXXXXXGWGKCAIQELFDAPLNEIMNNNQKKIASNIKREARNADYLMIWTD
CDREGEYIGWEIWQEAKRGNRLIQNDQVYRAVFSHLERQHILNAARNPSRLDMKSVHAVX
XXXXXXXXXXXXXXXLLTETLXXXXXXXXXXTKDGAKHRGGNKNDSQVVSYGTCQFPTLG
FVVDRFERIRNFVPEEFWYIQLVVENKDNGGXXXXQWDRGHLFDRLSVLTFYETCIETAG
NVAQXXXXXXXXXXXXXXXXXXTTVELQKNCARYLRLNAKQSLDAAEKLYQKGFISYPRTE
TDTFPHAMDLKSLVEKQAQLDQLAAGGRTAWASYXXXXXXXXXXXXXXXXXXXXXXXXXX
XXXXXXXXIVSLGPEANXXXXXXXXXXXXXXXXXXXXSEDAKGXXXXXXXXXXAVXXXXXX
XXXXXXXNFLDVYPWARWETTKQLPXXXXXXXXXXXXXXXXXGTTAPPKPMTESELILLM
DTNGIGTDATIAEHIDKIQVRNYVRSEKVGKETYLQPTTLGVSLVHGFEAIGLEDSFAKP
FQRREMEQDLKKICEGHASKTDVVKDIVEKYRKYWHKTNACKNTLLQVYDRVKASM

>YGL049C | TIF4632
MTDQRXXXXXXXXXXXXXXXXXXXDNQYSGANNSQPNNHYNENLYSAREPHNNKQYQSKN
GKYGTNKYNNRNNSQGNAQYYXXXXXXXXXXXXXXDYNPAMLPGMQWPANYYAPQMYYIPQ
QMVPVASPPYTHQPLNTNPEPPSTPKTTKIEITTKTGERLNLKKFHEEKKASKGEEKNDG
VXXXXXXXXXXXXXXXXVLPANEAVKDTLTETSNEKSTSEAENXXXXXXXXXXXXXXXXXX
XXXXXXXXXXXXXXXXXSNHDNTDTTKPNSVIESEPIKEAPKPTGEANEVVIDGKSXXXXX
XXXXXXXXXXXXXXFXXXXXXXXXXXXXXXXXXXXXXXXXXXXXXXGGKTVNKSDDETINSVI
TTEENTVKETEPSTSDIEMPTVSQLLETLGKAQPISDIYEFAYPENVERPDIKYKKPSVK
YTYGPTFLLQFKDKLKFRPDPAWVEAVSSKIVIPPHIARNKPKDSGRFGGDFRSPSMRGM
DHTSSSRVXXXXXXXXXXXXXXXSNRGYTXXXXXXXXXXXXXXXXXXXXXXXXLVPSANRWI
XXXXXXXXXXXXXXXXXXXELFDKEEVERKMKSLLNKLTLEMFDSISSEILDIANQSKWED
DGETLKIVIEQIFHKACDEPHWSSMYAQLCGKVVKDLDPNIKDKENEGKNGPKLVLHYLV
ARCHEEFEKGWADKLPAGEDGNPLEPEMMSDEYYIAAAAKRRGLGLVRFIGYLYCLNLLT
GKMMFECFRRLMKDLNNDPSEETLESVIELLNTVGEQFEHDKFVTPQATLEGSVLLDNLF
MLLQHIIDGGTISNRIKFKLIDVKELREIKHWNSAKKDAGPKTIQQIHQEEEQLRQKKNS
QRSNSRFNNHNQSNSNRYSSNRRNMQNTQRDXXXXXXXXXXXXXXXXXARKVEEVSQAPRA
NMFDALMNNDGDSD

>YBR093C | PHO5
MFKSVVYSILAASLANAGTIPLGKLADVDKIGTQKDIXXXXXXXXXXXXXXXXXXXISRDL
PEGCEMKQLQMVGRHGERYPTVSLAKTIKSTWYKLSNYTRQFNGSLSFLNXXXXXXXXXX
XXLEMETTFANSDDVLNPYTGEMNAKRHARDFLAQYGYMVENQTSFAVFTSNSKRCHDTA
QYFIDGLGDQFNITLQTVSEAESAGANTLSACNSCPAWDYDANDDIVNEYDTTYLDDIAK
RLNKENKGLNLTSTDASTLFSWCAFEVNAKGYSDVCDIFTKDELVHYSYYQDLHTYYHEG
PGYDIIKSVGSNLFNASVKLLKQSEIQDQKVWLSFTHDTDILNFLTTAGIIDDKNNLTAE
YVPFMGNTFHRSWYVPQGARVYTEXXXXXXXXXXXXXXXXXAVVPIETCSTGPGFSCEIND
FYDYAEKRVAGTDFLKVCNVSSVSNSTELTFYWDWNTTHYNASLLRQ

>YDR128W | MTC5 (SEA3)
MCSSINEGPYNSPTFGKSLSLKVDGGFNAVSINPSGRDIVLASRQGLYIIXXXXXXXXXX
XXXXXXXWQVADVQWSPHPAKPYWIVSTSNQKAIIWNLAKSSSNAIEFVLHGHSRAITDI
NFNPQHPDVLATCSVDTYVHAWDMRSPHRPFYSTSSWRSAASQVKWNYKDPNVLASSHGN
DIFVWDLRKGSTPLCSLKGHVSSVNSIDFNRFKYSEIMXXXNDGTVKFWDYSKSTTESKR
TVTTNFPIWRGRYLPFGEGYCIMPMXXXXXXXXXXXXXXXXXSEQNKKTKLQPIYAFKGH
SDRVIDFLWRSRHTCDGDYDDREFQLVTWSKDCDLKLWPXXXXXXXXXXXXXXXRLEEKL

PDYDYCSYNKEPENRENVQKNEFRRLRENFVXXXXXXXXXXXXXXXXXXXIRMNSATSQED
LFNETKIQNLGEEVSAIGHKFPKVVFEKISVSTRELCLTLNGPWSEENPDDYIFLRISIN
FPLNYPNKGDPPKFTIEENSNLTMSKRQEILSNLATIGQKYTDSNLYCLEPCIRFVLGEK
VSLEDIEEGQEPLLNFDIADHIDFEELSSLDSSYSDSQNPENLSSQSDIESYKEALVFPD
TSNQGLDFGRNLALDTTPVPNGCGSCWTATGELFCFFANXXXXXXXXXXXXXXXXXXXXGV
EKHPFKIEPQVLYDKEVDSSVITAADELXXXXXXXXXXXXXXXXXTNGXXXXXXXXXXXXX
XXXXXXXXXXXXILRNDIIVRTKIPILRGNFKAFXXXXXXXXXXXXXXTKXXXXXXXXXXX
XXXXXXRKELALEYLFMDATPEGFARNNALVAEKFDLDEISHXXXXXXXXXXXXXXXXXPY
TTIWNNHPMGIKWFIKEAIVXXXXXXXXXXXXXXXXXVILSARRKKIPARYYGQELENMEG
TIVFNDNESQNTSFWKGSDAFSTRSRSSTVTPNFYGNHLRGKNIHGGDNSSIRSDDHHAR
LRTHNTLNXXXXXXXXXXXXXXXXXXXXXPFHSRMPDIKVELLHDDIIEAYEQEDLLHLEV
SDIPKFQTYIYQYSKLLFRWGLPLERVKILKVSTDFXXXXXXXXXXXXXXXKKSPYNGVLT
HWIENNEFGEEKFLARNCNYCDLRVTRSSFICGNCQHVLHSSCARIWWEIGDECPSGCGC
NCPEMFDA

>YER155C | BEM2 (IPL2, SUP9, TSL1)
MKGLLWSKNXXXXXXXXXXXXXXXXSHKXXXXXXXXXXXXXXXXXXXXXXXXXSGASPYMXXX
XXXXXXXXXXXXXDNNRDKRKSSVFPPSKQYTSTSSSQVNLGMYHSDTNTRSSRSIASTL
KDDSPSVCXXXXXXXXXXXXXXXXXXXTPIAYXXXXXXXXXXXXXXXXXXLSPPQSRCXXXX
XXXXXXXXXXXXXXXXXXXXXXXXXXXXXXXXXXNVIHLNSENYDTTVFKTGWVNKSHGQX
XXXXXXXXXXXXXXXXXXXXXQNLRNDAYSRNRESRFYGXXXXXXXXXXXXXXXXXXXXXXX
XXXXXXXMAIDPQMLVPDYRLYRAQLKGCVLNLYKSGLNSNIKFFDPTLPASNSSIANEN
HQQKKQQTNNQAQAEALHQKQSFGQMGEPITLDLKYLSEVYPHPDLRQDSDGKIISGTIE
SLCHTVLFYPGPKQSDVPNEKSLSKTHRAVINLLLMFPLLDHFIKFLKVFNQFGLSFTKN
KSRLTNNSTQFYNISPAVDDSMTQRLALTAKTILDVFPGFLLDEPMLKTIISLLDTISLH
NDEISNNLKIKIANKHNELMKLTAFTRSLPMATSSXXXXXXXXXXXXXXXXXXXITTLADEV
HHINLKFDKVWAPKFDYSLLYDSKFINRRIVSLNPLVFNNDQNIHFLGRLLISHLFPTNP
EFSKKVTPKVRAELLDKWVQIGCRFEHLGDMVSWLAVATIICSIPVLRXXXWXXXXXXXXX
XXXXXXXXXTIIQLXXXXXXXXXXXXXXXXXXXXXXXXXXFTRANVISYFGDLLIHADDLPS
DTKFKYLEKKINRTKNAFHKWQQRLQAIDSTRHKTNSTENVRDNDSPNNVVYQLWKFHLS
QPPLNIEGIMKLSVQHEPPIIDQKAYSTIGSQRSALVTGSYLPIXXXXXXXXXXXXXXXXNT
LVGXXXXXXXXXXRXXXXXXXXXXXXXXXXXXASNSHTMGSLTDDAMSSKNDNNKVTGVGK
IDGPVIKEMSSXXXXXXXXXXXXXXXXXXXXXXXFHISDELVFKSVYDNDGKSRPASMVIE
TPKRFSQHSSMLINNPATPNQKMRDSLDTTGRLSKTLENMDFFNNIGQXXXXXXXXXXXX
XXXXXXLEKIFDLLVLTSNIFSKLVDTKDLENYYYHQRQRXXXXXXXXXXXXXXXXXXAFV
KLTMDNDXXXXXXXXXXXXXXXXXXXVLENMAKRYVGAKSCSVSISKILDRSDDSKMKINE
DTNLVSSSLYDQNFPVWDMKVTDDENINLIYMAKIQIGAAEAILHLVKNHYSDFTDDLCN
NSTLLDIIKIMEQEVSTEWPTRIANSKLQKSLPENFVIETENLLTTLTDLFHGIKSAYXX
XXXXXXXXXXXXXRIXXXXXXXXXXXXXXXXXXXXXXXXXXXXXXXXMIRSFQKLHSTNYEDILE
WIYQLDNFISKKFNLVSKKDWIVLFQELELLSKESLVSFFNYPLHFKSSKLINPGYLQLH
EFEISNLFTWISTLILKDDNGTESLFFEKLPQSIKLLIKLHTSLTTFFVMEISNVNKSSS
ERLTTCKVILQILNYIRWKNGSLDLFDSXXXXXXXXXXXXIPAFIETAIAHAIISPESRN
YELSWIKASEKLSDPTKGTQNLRSISNVLEKIDDIHIKRFIEIDDVFSKNCKNLCPCPGW
FISRLLEISQFVPNMSITNSKLINFDKRRFVNNIISNVLDLIPNEREFPLDIEMSDENPS
KRTTFGRILFNNFEDVNKVYXXXXXXXXXXXXXXXXXXXXXXXXXXXXXXXXXIEKIKREARK
LEVLLDQEKILKNSAALHQAVPKKNXXXXXXXXXXXXXXXXXYNINKNTGQTPSLGSVMES
NNSARNRRDSRASFSTNRSSVVSNSSHNGVXXXXXXXXXXXXXXXXXXNTXXXXXXXXXXX
XXXXXXXKSILPSILPEVDSMQLHDXXXXXXXXXXXXXXXXXXXINHRNIPAXXXAFKIVM
QNGHEYLIQTASSSDLTEWIKMIKASKRFSFHSKKYKGKTHNKIFGVPLEDVCERENTLI
PTIVVKLLEEIELRGLDEVGLYRIPXXXXXXXXXXXXXXXEEGATDNSFTLEDDRWFEVNA
IAGCFKMYLRELPDSLFSHAMVNDFTXXXXXXXXXXXXXXXXXRMMNELLQKLPTCYYQT
LKRXXXXXXXXXXXXXXXXXMDASNLAIVFSMSFINQEDLANSMGSRLGAVQTILQDFIKN
PNDYFKQ

>YGL105W | ARC1
MSDLVTKFESLIISKYPVSFTKEQSAQAAQWESVLKSGQIQPHLDQLNLVLRDNTFIVST
LYPTSTDVHVFEXXXXXXXXXXXXXXXXXXXXXXXXRHILRWIDYMQNLLEVSSTDKLEIN
HDLDLPHEVIEKKKKAPAGGAADAAAKADEDVSKKAKKQDHPRGKPDXXXXXXXXXXXXX
XXXXXXXXXXXQQQEQQNKAPEKPKPSAIDFRVGFIQKAIKHPDADSLYVSTIDVGDEEG
PRTVCSGLVKHFPLDAMQERYXXXXCNLKPVNMRGIKSTAMVLCGSNDDKVEFVEPPKDS
KAGDKVFFEGFGDEAPMKQLNPKKKIWEHLQPHFTTNDGLEVIFKDEEEKDHPVRKLTNA
KGESFKVASIANAQVR

>YGL136C | MRM2
MILVYNRIRSIIXXXXXXXXXXXXXXXXQXXXXXXXXXXXXXXXXXXVQNLRSRAAFKLMQI
DDKYRLFSKNRTDQRILDLGYAPGAWSQVARQRXXXXXXXXXXXXXXXXEPPHGVNSIQAN
ILAKRTHDLIRLFFSKHFQLNRHDDLHKDHGYFQNMLEEELTHVKDTELYREIFTSDDIY
ETPNTNSTLIEREKFPVDVIISDMYEPWPQTTGFWNNITNQAYFRMANTSGVSIRDHYQS
IDLCDAALVTAIDLLRPLGSFVCKLYTGXXXNLXXXXXXXXXXXXXXXXXPDASRDESKET
YYIGLKKKRNVDKLDVFSNS

>YGL154C | LYS5
MVKTTEVVSEVSKVAGVRPWAGIFVVEIQEDILADEFTFEALMRTLPLASQARILNKKSF
HDXXXXXXXXXXXXXXXXXIVTGLNFQELKFDKGSFGKPFLDNNRFLPFSMTIGEQYVAMF
LVKCVSTDEYQDVGIDIASPCNYGGREELELFKEVFSEREFNGLLKASDPCTIXXXXXXX
XXXXXXXXXXXXNXXXXXXXXXXXXXXXXXXXXSMCITLDEVPLIFHSQWFNNEIVTICMP
KSISDKINTNRPKLYNISLSTLIDYFIENDGL

>YGR092W | DBF2
MLSKSEKNVDLLAGNMSNLSFDGHGTPGGTGLFPNQNITKRRTRPAGINDSPSPVKPSFF
PYEDTSNMDIDEVSQPDMDVSNSPKKLPPKFYERATSNKTQRVVSVCKMYFLEHYCDMFD
YVISRRQRTKQVLEYLQQQSQLPNSDQIKLNEEWSSYLQREHQVLRKRRLKPKNRDFEMI
TQVGQGGYGQVYLARKKDTXXXXXXXXXXXXXXXXXXXXXXXXXXXXXXXXXXRSEWLVKL
LYAFQDLQSLYLAMEFVPGGDFRTLLINTRCLKSGHARFYISEMFCAVNALHDLGYTHRD
LKPENFLIDAKGHIKLTDFGLAAGTISNERIESMKIRLEKIKDLEFPAFTEKSIEDRRKM
YNQLREKEINYANSMVGSPDYMALEVLEGKKYDFTVDYWSLGCMLFESLVGYTPFSGSST
NETYDNLRRWKQTLRRPRQSDGRAAFSDRTWDLITRLIADPINRLRSFEHVKRMSYFADI
NFSTLRSMIPPFTPQLDSETDAGYFDDFTSEADMAKYADVFKRQDKLTAMVDDSAVSSKL
VGFTFRHRNGKQGSSGILFNGLEHSDPFSTFY

>YLR264W | RPS28B
MDSKTPVTLAKVIKVLGRTGSRGGVTQVRVEFLEDTSRTIVRNVKGPXXXXXXXXXXXXX
XXARRLR

>YOR070C | GYP1 (YOR29-21)
MGVRSAAKEMHERDHNXXXXXXXXXXXXXXXXXXXASSSKKPSLYKMNTTESTSLPSGYAS
SADRDRRTSDGNFEAMAKQQASTRRTSNSYSPLRYVNPTLSTASNESPRPALLLRQHHXX
XXXXQQPRHSSSGSVGNNCSNSTEPNKKGDRYFKDLDEDWSAVIDDYNMPIPILTNGGFG
TPVAPTRTLSRKSTXXXIXXXXXXXXXXXXXXXXXXXXXXXXXXXLQKEKTNDSKKTQLEIE
NERDVQELNSIIQRISKFDNILKDKTIINQQDLRQISWNGIPKIHRXXXXXXXXXXXXXX
XXXXXXXXXXXXXEYRDSLKHTFSDQHSRDIPTWHQIEIDXXXXXXXXXXXXXXXXXXXX
XXXXXLWAIRHPASGYVQGINDLVTPFFETFLTEYLPPSQIDDVEIKDPSTYMVDEQITD
LEADTFWCLTKLLEQITDNYIHGQPGILRQVKNLSQLVKRIDADLYNHFQNEHVEFIQFA
FRWMNCLLMREFQMGTVIRMWDTYLSETSQEVTSSYSMSSNDIKPPXXXXXXXXXXXXXX
XKDFQSPTTALSNMTPNNAVEDSGKMRQSSLNEFHVFVCAAFLIKWSDQLMEMDFQETIT
FLQNPPTKDWTETDIEMLLXXXXXXXXXXXXXXXXXXL

>YOR275C | RIM20
MSELLAIPLKRTLEVDFATELSKLXXXXXXXXXXXXXXXILKVVDARNNAIAPDISIDGL
SALKEYYVILLQLEKKFPNNQIEFTWFQTLSQKSRGTSQYSLQWEKLTIIYNIGCMYSLL
ALNSNNDAAESLKTSCLYFQNAAGCFKHVLDHQKNLETIPVVDDATLNALTSLMLAQAQE
CFWFKAVQDKHKDSLIAKLSQQIVDFYCEAINDAQRGKLIRSDWINHLKAKKAYFSAVTY
YRIALSFNEKKXXXXXXXXXXXXXXXXNESTLSSQAKFKTVVESSLKEAQRDNEFIYLQE
VXXXXXXXXXXXXXXXXXXXXXXXXIKKDETLFKDLIPIEVMXXXXXXXXXXXXXXXRV
TNPLASLNKLLKESXXXXXXXXXXXXXXXXXLSHYXXXXXXXXXXXKNVQVQLDNIEQIL
NEEAFTDNQLRLKHGTLNWTLPESSTTNTAYYXXXXXXXXXXXXXXXXXXXXXXXXXXX
XNLIGSEIRLPESNDPLTNKIKMIIQERNDYIDRTRRKSSEYRILPKIITSYKKNGTVDF
EPIFIGHLKYFDEDXXXXXXXXXXXXXXXXXVNLSKKNNPGRSGIEPKKMVRIDPRELYI
EDLRYSFKLLDEVKXXXXXXXXXXXXXXXXXNLYNEVQEYDTARRAEKARLDKSLTFED
Q

>YPL106C | SSE1 (LPG3, MSI3)
MSTPFGLDLGXXXXXXXXXXXXXXXXXXXXXXXXSTPSVVGFGPKNRYLGEXXXXXXXXX
XXXTVANLKRIIGLDYHHPDFEQESKHFTSKLVELDDKKTGXXXXXXGEKHVFSATQXXX
XXXXXXXXXXXXXXKANITDVCIAVPPWYTEEQRYNIADAARIAGLNPVRIVNDVTAAGV
SYGIFKTDLPEGEEKPRIVAFVDIGHSSYTCSIMAFKKGQLKVLGTACDXXXXXXXXXXX
XXXXXADEFKTKYKIDIRENPKAYNRILTAAEKLKKVLSANTNAPFSVESVMNDVDVSSQ
LSREELEELVKPLLERVTEPVTKALAQAKLSAEEVDFVEIIGGTTRIPTLKQSISEAFGK
PLSTTLNQDEAIAKGAAFICAIHSPTLRVRPFKFEDIHPYXXXXXXXXXXXXXHMEVFP
AGSSFPSTKLITLNRTGDFSMAASYTDITQLPPNTPEQIANWEITGVQLPEGQDSVPXXX
XXRCDPSGLHTXXXXXXXXXXXXXXPIPLXXXXXXXXXXXXXXXXXXXXXDDLTIVAHTF
GLDAKKLNELIEKENEMLAQDKLVAETEDRKNTLXXXXXXXXXXXXXXXAPFASDAEKTK
LQGMLNKAEEWLYDEGFDSIXXXXXXXXXXXXXXXXXXXXXXXXXXXXXXXXXXXXXXXX
XXXXXXXXXXXXXXXXXXXXXXXXXTEGDVDMD

>YDR034C | LYS14
MFESVNLDENXXXXXXXXXXXXXXXXXXXXXXXXXXXSFTNSGTSTSCFEPKNNLPSLSF
LNARAXXXXXXXXXXXXXXXXXXXIGGENVESTTSSNDGSNENXXXXXXXXXXXXXXXXX
ISQADDNGHSSLTPNPAVTSTVTDKKGNXXXXXXXXXXXXXXXXXXMKCDETKPTCWQCA
RLNRQCVYVLNPKNKKRRTSNAQRVKEFRKHSTSLDNDHNNAXXXXXXXXXXXXXXXXXX
NLSEDTTDPKPITDNGKNVPXXXXXXXXXXXXXXXXXXNGYDVNLLMQNLNDMVNMKLHD
SYLLNEELKGLDLPDLDIPELLPASNVNSSVPISFLVNNVITFNTKLSSFKLGGIHDKYL
KIFYYDCLDSIAPFFQNQGNPLRDILLSFAKNEAYLLSSILATGASIAYRKSNNLEDERN
YCAYLSHCLSLLGEQFKNESNVLNRIEPIILTVIMLAWDCIYSMNSQWRSHLKGVTDLFK
KINAGNSSKVLNVAKCWFKVMETFASISTVFGGSLIDNNDLDAIFDPYDYQYVDSLKFLN
IMTPLNEFNLLRGHKEDFDLVIKEVFKSLNTIRSTEKNYFSKEEGLFTKKLDYLLLSSQT
SSEKSKDQISYFNTQKILVEIDKQLDYEFIDKSGIIPSDNQSHPRISNIHDNAIDMVTLK
NGEEVAISXXXXXXXXXXXXXXXXXXXXXXGMPKESSTIQQVVKKIMSFFKFLDSDSPPQ
NSRTCYSNFAVLIAGLNAMDEETRAIVKRYYKINGGKFQKLTEHNLNRLEKVWYGKNQNY
RLEEQDVLTW

>YJL124C | LSM1 (SPB8)
MSANSKDRNQSNQDAKRQQQNFPKKISEGEADLYLDQYNFTTTAAIVSSVDRKIFVLLRD
GRMLFGVLRTFDQYANLILQDCVERIYFSXXXXXXXXXXXXXXXXXXXXXXXXXVDIDKE
DQPLEAMERIPFKEAWLTKQKNDEKRFKEETHKGKKMARHGIVYDFHKSDMY

>YGL045W | RIM8 (PAL3, YGL046W, ART9)
MSLLRLWNKESRAPSKIKSHGIVGSYGNSMLAHNNVKQFRIDIDEPHRVWKPNESITGEA
VIDIKRDITNVAIKLSLVCEVRVKTGNSPTSKNKRIEKTLEKSTFLYGQDYVKTAFSAKE

KKPHVDKTTILNGLSKGEHRFPFRIRIPRGRGMLSSIKFERGSITYFLSCTLESLNNING
LKKPEARCEREFAVIVPLDVSRLPKPKTKTVVLQSASMVQNKKNKSTEDESSSYTQLTQK
STTSNSSSSSVNSKTSPLPNKTVTISVDIPQAGFMIGEIIPIDVKIDHXXXXXXXXXXXX
XXXXXXXXXXXXXXXXXXXXXXXICQSISPIYINPETLQFQSRVYLKVPLDAFSTLTTVG
KFFSFQYYIEVMVNLSKKNVVYTESNRIIGTPIGEQNGLGVENNINRIQRKMLRMVNPET
LENDSEGYESSIFFKDMVNVEKLKRLRNVTGMSIETVIGTTRSEQQQSDASIPSQSSITA
PQNSPSNLRDWLAPLNAYDSDDVPVPKYSPNDKVSVPSEDKQELEQKRLQQLESDPPPCD
DY

Rangeof lowcomplexityregionswithp-values:

>YBL087C
135-137 p-value: 0.000e+00

>YAL018C
55-71 p-value: 3.583e-01
85-99 p-value: 4.927e-02
188-213 p-value: 5.761e-02

>YDL081C
65-81 p-value: 3.416e-15

>YDL136W
73-91 p-value: 1.474e-02

>YDR076W
53-68 p-value: 1.477e-02
152-154 p-value: 0.000e+00

>YLR181C
123-137 p-value: 3.642e-03
188-203 p-value: 7.773e-03
222-238 p-value: 2.896e-01
280-293 p-value: 2.045e-01
312-327 p-value: 4.125e-01

>YML062C
260-275 p-value: 3.766e-06
345-381 p-value: 2.494e-01
385-387 p-value: 0.000e+00

>YML063W
4-19 p-value: 1.504e-04
156-165 p-value: 1.587e-01
233-246 p-value: 5.227e-03

>YML121W
6-18 p-value: 2.628e-02
106-121 p-value: 3.739e-01
147-163 p-value: 3.768e-07
222-235 p-value: 8.926e-03
270-277 p-value: 2.437e-06

>YML017W
107-133 p-value: 3.523e-03
398-417 p-value: 1.589e-06
419-449 p-value: 4.673e-14
453-488 p-value: 1.160e-35
562-584 p-value: 1.753e-06

>YNL091W
5-21 p-value: 1.289e-10
26-42 p-value: 3.116e-02
71-103 p-value: 1.281e-01
144-166 p-value: 2.604e-03
206-230 p-value: 5.133e-02
452-483 p-value: 6.255e-16
524-538 p-value: 7.998e-09
556-580 p-value: 1.163e-30
617-635 p-value: 8.642e-02
637-668 p-value: 2.712e-70
671-690 p-value: 3.957e-11
692-709 p-value: 3.856e-10
712-730 p-value: 4.682e-04
732-755 p-value: 2.592e-14
992-1006 p-value: 1.856e-16

>YNR050C
105-122 p-value: 5.315e-01
263-279 p-value: 1.706e-01

>YNR051C
457-474 p-value: 2.093e-03

>YOL039W
67-99 p-value: 7.962e-06

>YOR106W
44-59 p-value: 4.544e-02
112-117 p-value: 1.344e-01
165-179 p-value: 2.032e-20
183-195 p-value: 1.421e-01
223-227 p-value: 1.214e-04
268-282 p-value: 1.654e-07

>YOR014W
16-55 p-value: 3.184e-43
85-104 p-value: 1.504e-17
106-122 p-value: 1.466e-05
126-141 p-value: 2.916e-10
143-147 p-value: 0.000e+00
303-321 p-value: 1.686e-01
550-576 p-value: 2.590e-02
679-701 p-value: 1.310e-01

>YOR038C
68-77 p-value: 1.977e-08
153-163 p-value: 6.273e-03
250-261 p-value: 1.094e-03
286-299 p-value: 2.306e-02
335-350 p-value: 5.636e-02
364-381 p-value: 5.410e-01
416-433 p-value: 5.955e-03
464-473 p-value: 2.976e-02
532-544 p-value: 2.096e-01
651-667 p-value: 1.021e-01
705-707 p-value: 0.000e+00
781-793 p-value: 3.567e-01

>YOR078W
158-173 p-value: 3.317e-02

>YOR382W
93-143 p-value: 3.373e-04

>YOR235W
34-49 p-value: 3.428e-03

>YOR293W
20-22 p-value: 0.000e+00

>YPL157W
7-20 p-value: 1.193e-06
56-58 p-value: 0.000e+00
83-94 p-value: 1.707e-02
277-290 p-value: 1.457e-04
292-303 p-value: 3.269e-03

>YPR172W
101-131 p-value: 2.137e-07

>YFL001W
49-80 p-value: 1.432e-01
83-98 p-value: 1.005e-01
422-437 p-value: 4.083e-01

>YNL284C
78-110 p-value: 2.240e-08
130-139 p-value: 1.788e-01
192-206 p-value: 2.714e-03
232-257 p-value: 2.871e-01
271-286 p-value: 3.359e-01

>YLR238W
25-51 p-value: 4.124e-01
110-115 p-value: 0.000e+00
281-309 p-value: 1.130e-01

>YBR201W
42-59 p-value: 4.060e-01

>YBR076W
136-150 p-value: 2.179e-01
157-174 p-value: 7.746e-02

>YBR106W
108-122 p-value: 3.375e-01

>YGL174W
21-42 p-value: 1.507e-06
49-68 p-value: 2.152e-02
179-181 p-value: 0.000e+00
200-217 p-value: 3.927e-01

>YGL199C
102-119 p-value: 8.051e-02

>YGL199C
102-119 p-value: 1.043e-01

>YGL031C
101-140 p-value: 5.496e-05

>YLR234W
21-23 p-value: 0.000e+00
62-75 p-value: 9.180e-02
180-195 p-value: 4.180e-01
202-211 p-value: 3.508e-02
272-275 p-value: 8.493e-17
305-321 p-value: 1.638e-01
395-428 p-value: 1.248e-02
438-457 p-value: 2.567e-01
464-472 p-value: 4.063e-02
475-487 p-value: 2.991e-01
506-522 p-value: 9.845e-02

>YGL049C
6-24 p-value: 1.031e-20
82-94 p-value: 8.293e-10
182-197 p-value: 1.659e-02
224-256 p-value: 5.860e-02
296-314 p-value: 1.244e-03
316-343 p-value: 8.565e-04
489-502 p-value: 3.374e-05
509-531 p-value: 3.174e-05
541-558 p-value: 3.331e-02
872-887 p-value: 4.075e-02

>YBR093C
38-55 p-value: 2.487e-01
111-122 p-value: 1.869e-18
385-400 p-value: 1.942e-02

>YDR128W
51-67 p-value: 2.241e-01
219-221 p-value: 0.000e+00
266-282 p-value: 8.873e-04
340-354 p-value: 1.831e-01
392-409 p-value: 5.340e-01
640-658 p-value: 2.933e-02
689-704 p-value: 3.624e-03
708-732 p-value: 2.591e-13
755-767 p-value: 4.268e-01
770-786 p-value: 5.685e-02
823-838 p-value: 3.381e-03
861-876 p-value: 2.368e-02
969-988 p-value: 2.156e-02
1057-1070 p-value: 1.519e-03

>YER155C
10-24 p-value: 3.391e-22
28-50 p-value: 8.634e-22
58-73 p-value: 1.722e-20
129-146 p-value: 2.870e-03
152-168 p-value: 4.605e-01
177-213 p-value: 7.774e-11
240-260 p-value: 9.957e-13
279-307 p-value: 4.444e-04
576-592 p-value: 3.403e-01
709-711 p-value: 0.000e+00
713-729 p-value: 7.680e-02
735-758 p-value: 9.180e-02
885-898 p-value: 1.638e-08
904-913 p-value: 5.864e-14
915-931 p-value: 4.790e-02
972-993 p-value: 2.488e-01
1069-1086 p-value: 1.662e-01
1121-1137 p-value: 1.832e-01
1148-1165 p-value: 1.044e-06
1319-1333 p-value: 4.308e-01
1336-1362 p-value: 4.338e-05
1529-1540 p-value: 1.261e-04
1701-1730 p-value: 3.698e-02
1766-1781 p-value: 1.615e-01
1831-1847 p-value: 8.076e-07
1850-1867 p-value: 7.333e-03
1886-1903 p-value: 1.613e-01
1912-1914 p-value: 0.000e+00
2006-2019 p-value: 2.001e-04
2067-2083 p-value: 2.790e-01
2104-2119 p-value: 3.595e-01

>YGL105W
73-95 p-value: 9.144e-02
168-191 p-value: 4.887e-20

262-265 p-value: 0.000e+00

>YGL136C
13-27 p-value: 5.693e-02
29-45 p-value: 2.108e-01
94-108 p-value: 3.091e-02
269-271 p-value: 0.000e+00
274-289 p-value: 2.612e-01

>YGL154C
63-78 p-value: 9.094e-07
174-192 p-value: 7.368e-02
194-212 p-value: 4.120e-01

>YGR092W
200-232 p-value: 3.249e-02

>YLR264W
48-62 p-value: 4.527e-01

>YOR070C
17-34 p-value: 7.808e-03
119-124 p-value: 1.087e-45
195-197 p-value: 0.000e+00
199-223 p-value: 5.656e-03
287-313 p-value: 9.641e-02
341-365 p-value: 1.629e-01
527-541 p-value: 2.681e-08
620-636 p-value: 1.888e-01

>YOR275C
25-39 p-value: 3.663e-02
252-267 p-value: 2.056e-02
302-325 p-value: 1.201e-10
343-358 p-value: 1.287e-01
375-391 p-value: 4.404e-01
396-406 p-value: 6.515e-03
453-481 p-value: 1.411e-01
555-571 p-value: 3.839e-01
615-632 p-value: 4.228e-03

>YPL106C
11-34 p-value: 1.032e-01
52-63 p-value: 1.333e-01
102-107 p-value: 6.683e-01
118-134 p-value: 4.555e-02
230-245 p-value: 3.052e-02
401-414 p-value: 1.255e-02
478-482 p-value: 2.903e-04
492-505 p-value: 8.929e-06
510-530 p-value: 3.344e-04
575-589 p-value: 3.617e-05
621-685 p-value: 8.506e-13

>YDR034C
11-37 p-value: 1.637e-03
66-84 p-value: 3.803e-01
104-166 p-value: 4.924e-02
223-278 p-value: 9.878e-02
669-690 p-value: 1.002e-02

>YJL124C
90-114 p-value: 2.532e-03

>YGL045W
289-323 p-value: 3.321e-01

Underlinedlowcomplexityregions:

>YBL087C
MSGNGAQGTKFRISLGLPVGAIMNCADNSGARNLYIIAVKGSGSRLNRLPAASLGDMVMA
TVKKGKPELRKKVMPAIVVRQAKSWRRRDGVFLYFEDNAGVIANPKGEMKGSAITGPVGK
ECADLWPRVASNSGVVV

>YAL018C
MSFTGSLALAGIGGLVYKFGGGQSYEKLPYVNIPFNQYLDKVYKKHFSKVMSRTRYVLMN
FFKDAFTGGAFMYPFKGFLEFNTNKSSYSTTMLGILSSYLIMFALVSFVYWATITPMYTA
FLIVLGPIGLFIAIFHSFLQANVFTLLFMRLSHFNNHLVEVCLEKNGLEENLSEVKPIKY
YAPINSIYFWAYYFPFKLVKYMLGLSVLFVLLVISFFPLIGPILFHILISPFITQIYFTK
VLRLQNFDNIQRRENIYLHAGQYASFGFLAGLIESVPILAGFAISTNTIGSVLFNLDHPM
VPENLVETQAEIEAAPQDINQQPNQ

>YDL081C
MSTESALSYAALILADSEIEISSEKLLTLTNAANVPVENIWADIFAKALDGQNLKDLLVN
FSAGAAAPAGVAGGVAGGEAGEAEAEKEEEEAKEESDDDMGFGLFD

>YDL136W
MAGVKAYELRTKSKEQLASQLVDLKKELAELKVQKLSRPSLPKIKTVRKSIACVLTVINE
QQREAVRQLYKGKKYQPKDLRAKKTRALRRALTKFEASQVTEKQRKKQIAFPQRKYAIKA

>YDR076W
MSLGIPLSQLIVESPKPLSSGITGLDEILNLGFQARSIYEIFGPPGIGKTNFGIQLVCNS
LEGIQQSEINDDKILWIETFQEMPINILRERFQKFKIVEENVKRVRITKFGQLLYFFQNL
FKLSQSVRYKLVIIDGFSQLVCDHLCTLSKRGGGMIDKTIHELKCRHLILIFTVMTKYTH
STGSTIIVLNDCMNTAFQSNEFESLEEYYEILDDGSNFFVNSNNERRKNNVHILKSALVA
NIAMGSKDSTWEVFLRDRIGLFRDWNEQVDETVFVKSKRVKASSSQSNEGCTTIKEMRIN
KRNFENLRIAIVFNLHGEDRKREGRNLKRSRSSDDRNYIVKFDFDKATGQLRDIIDLKPD
TANIASFPTLSTSSSSCSQVFNNIDSNDNPLPNAEGKEEIIYDSEG

>YLR181C
MASNAARVVATAKDFDKVGLGIIGYYLQLYAVELILSEEDRSQEMTALATELLDTIEAFK
KEIGGESEAEDSDKSLHVMNTLIHDQEKAKIYMLNFTMSLYNEKLKQLKDGPWDVMLKRS
LWCCIDLFSCILHLWKENISETSTNSLQKRIKYCKIYLSKLAKGEIGSSDEKTLDYADFA
DDSEEIKDEDVDHQTSDLENNNNDKVEGLAPKDQTTSYEPVDEVPEFIDDADSVNEEEQT
VDKNEDAITKDEQQVVKKEVDLTRPSAPSEPAAAEHKSYTKDELTKIMDRASKIEQIQKL

AKYAISALNYEDLPTAKDELTKALDLLNSI

>YML062C
MPLSQKQIDQVRTKVHYSEVDTPFNKYLDILGKVTKLTGSIINGTLSNDDSKIEKLTEQN
ISQLKESAHLRFLDLQSSIDTKKVADENWETCQQETLAKLENLKDKLPDIKSIHSKLLLR
IGKLQGLYDSVQVINREVEGLSEGRTSLVVTRAEWEKELGTDLVKFLIEKNYLKLVDPGL
KKDSSEERYRIYDDFSKGPKELESINASMKSDIENVRQEVSSYKEKWLRDAEIFGKITSI
FKEELLKRDGLLNEAEGDNIDEDYESDEDEERKERFKRQRSMVEVNTIENVDEKEESDHE
YDDQEDEENEEEDDMEVDVEDIKEDNEVDGESSQQEDNSRQGNNEETDKETGVIEEPDAV
NDAEEADSDHSSRKLGGTTSDFSASSSVEEVK

>YML063W
MAVGKNKRLSRGKKGLKKKVVDPFTRKEWFDIKAPSTFENRNVGKTLVNKSTGLKNASDA
LKGRVVEVCLADLQGSEDHSFRKVKLRVDEVQGKNLLTNFHGMDFTTDKLRSMVRKWQTL
IEANVTVKTSDDYVLRIFAIAFTRKQANQVKRHSYAQSSHIRAIRKVISEILTREVQNST
LAQLTSKLIPEVINKEIENATKDIFPLQNIHVRKVKLLKQPKFDVGALMALHGEGSGEEK
GKKVSGFKDEVLETV

>YML121W
MSSNNRKKLLLMGRSGSGKSSMRSIIFSNYSAFDTRRLGATIDVEHSHLRFLGNMTLNLW
DCGGQDVFMENYFTKQKDHIFQMVQVLIHVFDVESTEVLKDIEIFAKALKQLRKYSPDAK
IFVLLHKMDLVQLDKREELFQIMMKNLSETSSEFGFPNLIGFPTSIWDESLYKAWSQIVC
SLIPNMSNHQSNLKKFKEIMNALEIILFERTTFLVICSSNGENSNENHDSSDNNNVLLDP
KRFEKISNIMKNFKQSCTKLKSGFKTLILNNNIYVSELSSNMVCFIVLKDMNIPQELVLE
NIKKAKEFFQ

>YML017W
MGTNNTSNNNGTTKKMSLEEFLGNDTLGESVWDEEDINLDAISNTTNIDILKQTKAGEHQ
RDGHQQHPHGGHGPMNRSRFSNAGPFGGGSMGDFANHHHPLQHQQGPPYIVKFSDLPPRF
SNFDIEDLFQAKFTKFIKFKLFWEINKNPSISTLKSGSIFDQNFKRDSKVAFVELYTSRD
MDKILNYWTTPLKEIYHITTAPAEFEDFKDYSTKVKLLTDPKDDAGKPFITKTQRSKSNP
FGSAKPVDTQSKILDIEEKMENLHVEDTTTLRASLIPSSDSMATTATGSKITILKKQTPT
EEESHSATPTPKPLSYSEVVERSVVNETSKKGTPLSKLDSPALELQSKPDKSDEFKGGDE
QGFEKGGDDKAQLDVSNDKDKGSETDVDKQFTFKNVEREHSMSRTKYNGNHNNNNGNFRG
SNRYRGGPNGSSYKGGHNNRGNRGGYRGGSSYNNNNNNTNDNNNNNNNNSSSNNNNGSRYH
DRQNNEEGLTSDSSLDASGNKKNDFTNSTSNTQQYSIFKPASGFLGQGNNDSIRNNGRGN
YNSSGMNGGSRGRGFGRGRGFGRGAYNNRGSRGGRGSSGNYSNYNNRTTDMPL

>YNL091W
MPPNSKSKRRKNKSKQHNKKNGNSDPEQSINPTQLVPRMEPELYHTESDYPTSRVIKRAP
NGDVIVEPINTDDDKKERTANLTHNKDSMDSASSLAFTLDSHWESLSPEEKKTILRIEKE
EVFNVIRNYQDDHSCSCSVCGRRHLAMDQEMERIYNTLYAMDKDKDPETNPIKFHLGIIK
ELQISKNQQQNDLSSTKGEVVKNFLSSSTVGSLKEEVLHFKQKQLSKQEQAHNETADNTS
LLEENLNNIHINKTSSEISANFNSVSDEELQQKYSNFTKTFISSHPKIAEEYVQKMMMYP
NIRALTDDLMNSNGQGFLNAIEDFVRDGQIQASKKDDSITEDEASSTDLTDPKEFTTMLH
SGKPLTEDEYADLQRNIAERMTNAYDTASKKFKDVSQLEKELFTRFMSGRDKKSFRELII
QSFKNKFDGELGPSVLAATLSSCFSSQSKDTSLDTDSIYEDEDEEDYDDYSEYAEDSEEV
SEYEGIEAVEKPEHDEKSNGIRETLHLSYDHDHKRQNHPHHHYHSTSTHSEDELSEEEYI
SDIELPHDPHKHFHRDDDILDGDEDEPEEEDENEGDDEEDTYDSGLDETDRLEEGRKLIQ
IAITKLLQSRIMASYHEKQADNNRLKLLQELEEEKRKKREKEEKKQKKREKEKEKKRLQQ
LAKEEEKRKREEEKERLKKELEEREMRRREAQRKKVEEAKRKKDEERKRRLEEQQRREEM
QEKQRKQKEELKRKREEEKKRIREQKRLEQEKLQKEKEEEERQRLIAEDALRKQKLNEEQ
TSANILSAKPFTENGVGNPVSSQSHPNMTNYQEDNSCSINDEILKMVNSVAASKPVSPTG

FNVHDLLLPSTNNQMPAMEQSHLPQPGNQNNHFGTTTIPNALDLATKSSLQTENNYLMNS
QTLENTSLLMHNNSSPTKLLPNDFGLSSWGGLTNTMSINPTCKPPVIQTSEMESQAHKSS
PQATMPSFGLPNGGTHRKSFTDELNTLTSMLSSSGFADTSLSSSGFPPSQRSVWNDQKSS
FSGPSTAGNFNNSSIQSGMLLAPTLGSVESFPNRTSIWDSSTTPMMNKSELSGRNITSTA
QDSPAFMASNIWSSNSQYNSPYLTSNVLQSPQISSGVDESHILDSIYNTYLAISPQDSLN
PYIAIGTLFQNLVGLNLDYSTFINKLISMQGAYNCEFFTDNNGSITHVRFARQTPAGHSK
GLLNQLFSGLNDPTATPFTSRPHTSTRASFPIASSTTQTS

>YNR050C
MGKNVLLLGSGFVAQPVIDTLAANDDINVTVACRTLANAQALAKPSGSKAISLDVTDDSA
LDKVLADNDVVISLIPYTFHPNVVKSAIRTKTDVVTSSYISPALRELEPEIVKAGITVMN
EIGLDPGIDHLYAVKTIDEVHRAGGKLKSFLSYCGGLPAPEDSDNPLGYKFSWSSRGVLL
ALRNSAKYWKDGKIETVSSEDLMATAKPYFIYPGYAFVCYPNRDSTLFKDLYHIPEAETV
IRGTLRYQGFPEFVKALVDMGMLKDDANEIFSKPIAWNEALKQYLGAKSTSKEDLIASID
SKATWKDDEDRERILSGFAWLGLFSDAKITPRGNALDTLCARLEELMQYEDNERDMVVLQ
HKFGIEWADGTTETRTSTLVDYGKVGGYSSMAATVGYPVAIATKFVLDGTIKGPGLLAPY
SPEINDPIMKELKDKYGIYLKEKTVA

>YNR051C
MGVTVQDICFAFLQNYYERMRTDPSKLAYFYASTAELTHTNYQSKSTNEKDDVLPTVKVT
GRENINKFFSRNDAKVRSLKLKLDTIDFQYTGHLHKSILIMATGEMFWTGTPVYKFCQTF
ILLPSSNGSTFDITNDIIRFISNSFKPYVLTDASLSQSNEENSVSAVEEDKIRHESGVEK
EKEKEKSPEISKPKAKKETVKDTTAPTESSTQEKPIVDHSQPRAIPVTKESKIHTETVPS
STKGNHKQDEVSTEELGNVTKLNEKSHKAEKKAAPIKTKEGSVEAINAVNNSSLPNGKEV
SDEKPVPGGVKEAETEIKPIEPQVSDAKESGNNASTPSSSPEPVANPPKMTWASKLMNEN
SDRISKNNTTVEYIRPETLPKKPTERKFEMGNRRDNASANSKNKKKPVFSTVNKDGFYPI
YIRGTNGLREEKLRSALEKEFGKVMRITAADNFAVVDFETQKSQIDALEKKKKSIDGIEV
CLERKTVKKPTSNNPPGIFTNGTRSHRKQPLKRKD

>YOL039W
MKYLAAYLLLNAAGNTPDATKIKAILESVGIEIEDEKVSSVLSALEGKSVDELITEGNEK
LAAVPAAGPASAGGAAAASGDAAAEEEKEEEAAEESDDDMGFGLFD

>YOR106W
MSFFDIEAQSSKGNSQQEPQFSTNQKTKELSNLIETFAEQSRVLEKECTKIGSKRDSKEL
RYKIETELIPNCTSVRDKIESNILIHQNGKLSADFKNLKTKYQSLQQSYNQRKSLFPLKT
PISPGTSKERKDIHPRTEAVRQDPESSYISIKVNEQSPLLHNEGQHQLQLQEEQEQQQQG
LSQEELDFQTIIHQERSQQIGRIHTAVQEVNAIFHQLGSLVKEQGEQVTTIDENISHLHD
NMQNANKQLTRADQHQRDRNKCGKVTLIIIIVVCMVVLLAVLS

>YOR014W
MMRGFKQRLIKKTTGSSSSSSSKKKDKEKEKEKSSTTSSTSKKPASASSSSHGTTHSSAS
STGSKSTTEKGKQSGSVPSQGKHHSSSTSKTKTATTPSSSSSSSRSSSVSRSGSSSTKKT
SSRKGQEQSKQSQQPSQSQKQGSSSSSAAIMNPTPVLTVTKDDKSTSGEDHAHPTLLGAV
SAVPSSPISNASGTAVSSDVENGNSNNNNMNINTSNTQDANHASSQSIDIPRSSHSFERL
PTPTKLNPDTDLELIKTPQRHSSSRFEPSRYTPLTKLPNFNEVSPEERIPLFIAKVDQCN
TMFDFNDPSFDIQGKEIKRSTLDELIEFLVTNRFTYTNEMYAHVVNMFKINLFRPIPPPV
NPVGDIYDPDEDEPVNELAWPHMQAVYEFFLRFVESPDFNHQIAKQYIDQDFILKLLELF
DSEDIRERDCLKTTLHRIYGKFLSLRSFIRRSMNNIFLQFIYETEKFNGVAELLEILGSI
INGFALPLKEEHKVFLVRILIPLHKVRCLSLYHPQLAYCIVQFLEKDPLLTEEVVMGLLR
YWPKINSTKEIMFLNEIEDIFEVIEPLEFIKVEVPLFVQLAKCISSPHFQVAEKVLSYWN
NEYFLNLCIENAEVILPIIFPALYELTSQLELDTANGEDSISDPYMLVEQAINSGSWNRA
IHAMAFKALKIFLETNPVLYENCNALYLSSVKETQQRKVQREENWSKLEEYVKNLRINND

KDQYTIKNPELRNSFNTASENNTLNEENENDCDSEIQ

>YOR038C
MRLLKYPLDIHNEQVNALAALGPYIILAGSGGHVMAWRQQQLVDTAFDRVMIKDLKPEVS
FQVDQDTTGDIFFITGDLETLYIGSEHRLWGYSGWLCRDTNNINSVEKMNSKLLFECKSP
STITDVKYDINLGILFVLLSNENKILLFRHKTFDKLSEITIDKASKPITGIIDPTGQTFT
VMTSDRSILVYQINKTGTHKLINKLTQHVQMYPLHYRISMSPQADILPVINSVKGVPNNA
TSCTALLDRNNNYKVTKTLVTPSSNGCRVLVYSPAFYEKPNLKKGTSTRYNLIATSGSTD
GTILVWNTKRMKPLFNALQVSSTAINDMSWSQDGFTLFAISNDATLYTFAFQEKDLGVAL
PQTEIKSLQEVNKKLPKLEEPLAEQIPKSFPENIKLEESASAAPIPNDIGRSAVGKKPTK
KKTANNQTNGIKTIQSTSMEFNTPSYTVPRDLKRKPKEATPSNIAPGSKKQKKELQPIDF
LDTGLLLPNTSFSRIRLATPKIRSTFKYSPINNPNLILDVKNGSGNEQRPTIVKLTSKVL
DQDQVLFQDFIPKLITICTAGDTFWSFCSEDGSIYIYSDSGRKLMAPLVLGVSISFLEAC
GTYLLCLTSIGELYCWNIEQKKLAFPTNTIYPLLNPSLRYSDDILTRAENITLCSITKKG
VPLVTLSNGDGYLFDKNMETWLLVSDGWWAYGSQYWDTTNTTGLSSSKANTDSFNGSESN
INEIVSDIKNDNQSIINFLECKTNDELNRKGRIKNLQRFARTILMKEGFENMEEIVTLSH
LENKILISIRLEEPEEFSKLMMVYCIRLSELGYMDRLNDVFQWLYDDLPISGTGSAFADK
DFKRNLLKKILIACGDIRQVQRVTTRYAKEMNIIS

>YOR078W
MSNGHVKFDADESQASASAVTDRQDDVLVISKKDKEVHSSSDEESDDDDAPQEEGLHSGK
SEVESQITQREEAIRLEQSQLRSKRRKQNELYAKQKKSVNETEVTDEVIAELPEELLKNI
DQKDEGSTQYSSSRHVTFDKLDESDENEEALAKAIKTKKRKTLKNLRKDSVKRGKFRVQL
LSTTQDSKTLPPKKESSIIRSKDRWLNRKALNKG

>YOR382W
MKFSTIFGATTVMTAVSAAAVSSVMTTKTITATNGNNVYTKVVTDTADPIISYSTTRTVV
VSNSDATYTKVVTEGPDTTSEKSTTKTLTLTNGSGSSTNLYTKTVTQAVESSTSSSSSSS
SSSSSASSSGAAPAAFQGASVGALALGLISYLL

>YOR235W
MGLYRPSKFFHPPIPHIPFTINPDFFSFHIQRLKAKANPENFLICFPPPDIYKGFVFCCQ
LDLVHLFSYVFFLFLLKICVDVLQYVIYPKHFTHKKPGFENYSI

>YOR293W
MLMPKEDRNKIHQYLFQEGVVVAKKDFNQAKHEEIDTKNLYVIKALQSLTSKGYVKTQFS
WQYYYYTLTEEGVEYLREYLNLPEHIVPGTYIQERNPTQRPQRRY

>YPL157W
MGRTFIHASKIKHAARKRKHHSNFRTLIKLLNNDAYKIESSKPLKNGKLFKYWKNRRRLF
SKIDSASIYMTDELWFSVTPERIACFLANFVKACMPNAERILDVFCGGGGNTIQFAMQFP
YVYGVDYSIEHIYCTAKNAQSYGVDDRIWLKRGSWKKLVSKQKLSKIKYDCVFGSPPWGG
PEYLRNDVYDLEQHLKPMGITKMLKSFLKLSPNVIMFLPRNSDLNQLSRATRKVLGPFAK
CKVLYVKENGYMKGIFCMWGECFFNYEPASTENSRRESSEKEELSSENEELSKRKKHEST
TTTKDNTVDIYDVNG

>YPR172W
MAWTSTLPAHLLNLIKNSKYVHVATCSKDCIPSVALMNYIYVPGEKLFGQTDNKNDYIIF
VSPQDTQKFYNIKENPKVALLFHDWIIANNLSVGKESISGTPTPTSIPHEEQRQSELLNL
LQELNQAELNQMSASIGGETEIVNPESEESKYYKDLILKANPDAKAFIFEKNTAVVKVRI
DNARVSNNENRTMFLSKGKS

>YFL001W

MSNFIRRLVGKMKAISTGTNAIVSKKDSIYANWSKEQLIRRITELENANKPHSEKFQHIE
DNKKRKISQEEVTRSKAKKAPKKFDFSKHNTRFIALRFAYLGWNYNGLAVQKEYTPLPTV
EGTILEAMNKCKLVPSMVLQDYKFSRCGRTDKGVSAMNQVISLEVRSNLTDEEQRDPTND
SREIPYVHVLNQLLPDDIRISAVCLRPPPNFDARFSCVHRHYKYIFNGKNLNIEKMSKAA
SYFVGERDFRNFCKLDGSKQITNFKRTIISSKILPLSETFYCFDLVGSAFLWHQVRCMMA
ILFLVGQSLEVPEIVLRLTDIEKTPQRPVYEMANDIPLLLYDCKFPEMDWQEPTVDDYKA
IKFTTATEALTLHYELKAAVCNIFKDVLPTANTNNFSKTIINLGDGRGKVVGTYVKLEDR
SVMEPVEVVNAKYSKKKNNKNK

>YNL284C
MKAERQTGLRNSFTTVIGRKLINTFVPSMMLTSVAGNDIFFRGLFKSPVLAFQSYRYVSI
LGQLKPSDGSTKSFKRLGRGPSSGLGKTSGRGQKGQKARGKVKSWFEGGQTPIYKLFPKI
GFTNVGAKPLKELNLKRIQWFHDKNRLHLQPGEVLDMNKMRKLGLVTGPIKYGVKILASG
KFHYNLPIALEASRASAKAIAAIEKAGGKFTARYYTPLGLRAHLNPQWFLEKRGRVPLQA
RPTKRRDIDFYSKEEKRGYLVMEKDKLLQDIKEAQNKGSRHFLKQNVKKSSLEIELEELS
PEKDWVPVVSNSKVMNIKALDH

>YLR238W
MTGPGPEINKEEHPSSPGKKQITYNSIPKNANLIDGSTNSSKRPIEKYDKRIADPTKSYF
PHSISRTPRRKYTYILVLTSLNGTFESKHVVIPFKPDGLKLGRPVANSNSSSSSSLRGGK
RVDSHTFSQVRSDNGNFDSRVLSRNHALLSCDPLTGKVYIRDLKSSNGTFINGQRIGSND
VEIKVGDVIDLGTDIDTKIEHRKISATVEELFVQPLLESPIFENEDSDDCHTITEKEEAA
AITSHIYGDSNNLELEEVILGSDTEILSGIFINNCIGTSPTLSNIIKTLAMEIPFSKCDN
FKLQSMENFLINYTTHLEYTNKLLVEKNDQQLVKLQNGLRRKLSGKYEKIIEQNRNQVKQ
LERDHMFFKKSFEVKKRRNNEKQKSMEREIEDLKTRLEVERYKNSQMMKKNKQKEQELST
ASKKKTTEHDTRGVPGMNPKGTDKFSIKNTLCNHFTLLTFGTISIGIIAIVFKILSPN

>YBR201W
MDAVILNLLGDIPLVTRLWTIGCLVLSGLTSLRIVDPGKVVYSYDLVFKKGQYGRLLYSI
FDYGAFNWISMINIFVSANHLSTLENSFNLRRKFCWIIFLLLVILVKMTSIEQPAASLGV
LLHENLVYYELKKNGNQMNVRFFGAIDVSPSIFPIYMNAVMYFVYKRSWLEIAMNFMPGH
VIYYMDDIIGKIYGIDLCKSPYDWFRNTETP

>YBR076W
MDYGYFFPAQRIEETNGVDFWIDSNAEFTQSKRPDSSTSTLSRVLTDTTNVSNNSGSLKR
KTIKNKIFPQRKIFNDSENFDFGKANTDCKHVFKSISKQLIFLPRCFQHHSIRGWMKDRY
SEFGYKIKRNQNCPPSACVQALYNTSRSNTEESNPNSLDSLIMYKYMRYSEKKKELMCRF
CQGNNWILAENYLKHLFFAHGILSEFKPHTLYHFESKLLKIQGKLNFKIQVLKEPEFSKK
ILNSLTVSIIPSPLAYYTQTLNGGFRRIHVKCPHCENWIRLGWCEYDEIIRDSFQDFESL
RNLNADYNGMSYIQTRNREDIEGIYENYFTHYIQCDLATFRTKCLYVQVITKSN

>YBR106W
MNPQVSNIIIMLVMMQLSRRIDMEDPTIIMYIRILYCSSIGISWIIYQMARKRIVAKNDM
TTMKYVEPGNAMSGEGEKLQVTTVRDYDLKEIDSAIKSIYTGMAMMGFMHLYLKYTNPLF
MQSISPVKSALEHNEVKIHLFGKPATGDLKRPFKAPSLFGGMGQTGPKTDKKSIEEAERA
GNAGVKAE

>YGL174W
MALHQYLSETYGPTKPKNKTKKKKKESKSDANSDKTSLIVKERLSTLQQEQEKSGVASFS
KFDKQKSKNIWKNLETNELSHAITHPSASSITGNESKNDLKEIRAQEPLVTVADKSKTRK
TIYRDAQGHKIQEDSKIDDSSFSRSKYEDEKAAEREQYLKNLNMGDVQKLGINVDAHDKK
KNQTASSLTIEDPAITFTHDKERTVKTSLLGRKLYDKPAPENRFAIMPGSRWDGVHRSNG
FEEKWFAKQNEINEKKVQSYTLQEDY

>YGL199C
MEMYEISDTLWCGLVSLRCNAITSKMNQLDTNRLENSDRACDTNREYNMINARGAHRSQL
AEMSLSAAPIPSSEGLKNEGKCGYTTCFSLELISTTSRFSVCSTVSSSVPLLPEFCIVPP
MLSATPPEAAKGASGSMKSGSIVVSSLPYDILPALL

>YGL199C
MEMYEISDTLWCGLVSLRCNAITSKMNQLDTNRLENSDRACDTNREYNMINARGAHRSQL
AEMSLSAAPIPSSEGLKNEGKCGYTTCFSLELISTTSRFSVCSTVSSSVPLLPEFCIVPP
MLSATPPEAAKGASGSMKSGSIVVSSLPYDILPALL

>YGL031C
MKVEIDSFSGAKIYPGRGTLFVRGDSKIFRFQNSKSASLFKQRKNPRRIAWTVLFRKHHK
KGITEEVAKKRSRKTVKAQRPITGASLDLIKERRSLKPEVRKANREEKLKANKEKKKAEK
AARKAEKAKSAGTQSSKFSKQQAKGAFQKVAATSR

>YLR234W
MKVLCVAEKNSIAKAVSQILGGGRSTSRDSGYMYVKNYDFMFSGFPFARNGANCEVTMTS
VAGHLTGIDFSHDSHGWGKCAIQELFDAPLNEIMNNNQKKIASNIKREARNADYLMIWTD
CDREGEYIGWEIWQEAKRGNRLIQNDQVYRAVFSHLERQHILNAARNPSRLDMKSVHAVG
TRIEIDLRAGVTFTRLLTETLRNKLRNQATMTKDGAKHRGGNKNDSQVVSYGTCQFPTLG
FVVDRFERIRNFVPEEFWYIQLVVENKDNGGTTTFQWDRGHLFDRLSVLTFYETCIETAG
NVAQVVDLKSKPTTKYRPLPLTTVELQKNCARYLRLNAKQSLDAAEKLYQKGFISYPRTE
TDTFPHAMDLKSLVEKQAQLDQLAAGGRTAWASYAASLLQPENTSNNNKFKFPRSGSHDD
KAHPPIHPIVSLGPEANVSPVERRVYEYVARHFLACCSEDAKGQSMTLVLDWAVERFSAS
GLVVLERNFLDVYPWARWETTKQLPRLEMNALVDIAKAEMKAGTTAPPKPMTESELILLM
DTNGIGTDATIAEHIDKIQVRNYVRSEKVGKETYLQPTTLGVSLVHGFEAIGLEDSFAKP
FQRREMEQDLKKICEGHASKTDVVKDIVEKYRKYWHKTNACKNTLLQVYDRVKASM

>YGL049C
MTDQRGPPPPHPQQANGYKKFPPHDNQYSGANNSQPNNHYNENLYSAREPHNNKQYQSKN
GKYGTNKYNNRNNSQGNAQYYNNRFNNGYRLNNNDYNPAMLPGMQWPANYYAPQMYYIPQ
QMVPVASPPYTHQPLNTNPEPPSTPKTTKIEITTKTGERLNLKKFHEEKKASKGEEKNDG
VEQKSKSGTPFEKEATPVLPANEAVKDTLTETSNEKSTSEAENTKRLFLEQVRLRKAAME
RKKNGLISETEKKQETSNHDNTDTTKPNSVIESEPIKEAPKPTGEANEVVIDGKSGASVK
TPQHVTGSVTKSVTFNEPENESSSQDVDELVKDDDTTEISDTTGGKTVNKSDDETINSVI
TTEENTVKETEPSTSDIEMPTVSQLLETLGKAQPISDIYEFAYPENVERPDIKYKKPSVK
YTYGPTFLLQFKDKLKFRPDPAWVEAVSSKIVIPPHIARNKPKDSGRFGGDFRSPSMRGM
DHTSSSRVSSKRRSKRMGDDRRSNRGYTSRKDREKAAEKAEEQAPKEEIAPLVPSANRWI
PKSRVKKTEKKLAPDGKTELFDKEEVERKMKSLLNKLTLEMFDSISSEILDIANQSKWED
DGETLKIVIEQIFHKACDEPHWSSMYAQLCGKVVKDLDPNIKDKENEGKNGPKLVLHYLV
ARCHEEFEKGWADKLPAGEDGNPLEPEMMSDEYYIAAAAKRRGLGLVRFIGYLYCLNLLT
GKMMFECFRRLMKDLNNDPSEETLESVIELLNTVGEQFEHDKFVTPQATLEGSVLLDNLF
MLLQHIIDGGTISNRIKFKLIDVKELREIKHWNSAKKDAGPKTIQQIHQEEEQLRQKKNS
QRSNSRFNNHNQSNSNRYSSNRRNMQNTQRDSFASTKTGSFRNNQRNARKVEEVSQAPRA
NMFDALMNNDGDSD

>YBR093C
MFKSVVYSILAASLANAGTIPLGKLADVDKIGTQKDIFPFLGGAGPYYSFPGDYGISRDL
PEGCEMKQLQMVGRHGERYPTVSLAKTIKSTWYKLSNYTRQFNGSLSFLNDDYEFFIRDD
DDLEMETTFANSDDVLNPYTGEMNAKRHARDFLAQYGYMVENQTSFAVFTSNSKRCHDTA
QYFIDGLGDQFNITLQTVSEAESAGANTLSACNSCPAWDYDANDDIVNEYDTTYLDDIAK
RLNKENKGLNLTSTDASTLFSWCAFEVNAKGYSDVCDIFTKDELVHYSYYQDLHTYYHEG

PGYDIIKSVGSNLFNASVKLLKQSEIQDQKVWLSFTHDTDILNFLTTAGIIDDKNNLTAE
YVPFMGNTFHRSWYVPQGARVYTEKFQCSNDTYVRYVINDAVVPIETCSTGPGFSCEIND
FYDYAEKRVAGTDFLKVCNVSSVSNSTELTFYWDWNTTHYNASLLRQ

>YDR128W
MCSSINEGPYNSPTFGKSLSLKVDGGFNAVSINPSGRDIVLASRQGLYIIDLDDPFTPPR
WLHHITPWQVADVQWSPHPAKPYWIVSTSNQKAIIWNLAKSSSNAIEFVLHGHSRAITDI
NFNPQHPDVLATCSVDTYVHAWDMRSPHRPFYSTSSWRSAASQVKWNYKDPNVLASSHGN
DIFVWDLRKGSTPLCSLKGHVSSVNSIDFNRFKYSEIMSSSNDGTVKFWDYSKSTTESKR
TVTTNFPIWRGRYLPFGEGYCIMPMVGGNNAVYLINLCDDDDSEQNKKTKLQPIYAFKGH
SDRVIDFLWRSRHTCDGDYDDREFQLVTWSKDCDLKLWPISDSIYGKVNFDRGKRLEEKL
PDYDYCSYNKEPENRENVQKNEFRRLRENFVTTSGLKKNKTNHITWLSGIRMNSATSQED
LFNETKIQNLGEEVSAIGHKFPKVVFEKISVSTRELCLTLNGPWSEENPDDYIFLRISIN
FPLNYPNKGDPPKFTIEENSNLTMSKRQEILSNLATIGQKYTDSNLYCLEPCIRFVLGEK
VSLEDIEEGQEPLLNFDIADHIDFEELSSLDSSYSDSQNPENLSSQSDIESYKEALVFPD
TSNQGLDFGRNLALDTTPVPNGCGSCWTATGELFCFFANEKKPEKKQNAIIKLSQKEAGV
EKHPFKIEPQVLYDKEVDSSVITAADELKARPKRYVDTLGLGGGTNGDSRTYFDDETSSD
DSFDSVADDWDDILRNDIIVRTKIPILRGNFKAFSSVHSESGKTVESTKKNKNLVISKNF
SSLLSDRKELALEYLFMDATPEGFARNNALVAEKFDLDEISHCWQILSDMLIDQSDYDPY
TTIWNNHPMGIKWFIKEAIVYFERQQNLQMLAMLCCVILSARRKKIPARYYGQELENMEG
TIVFNDNESQNTSFWKGSDAFSTRSRSSTVTPNFYGNHLRGKNIHGGDNSSIRSDDHHAR
LRTHNTLNGSSKFTEPAQKQGSRAISSSPFHSRMPDIKVELLHDDIIEAYEQEDLLHLEV
SDIPKFQTYIYQYSKLLFRWGLPLERVKILKVSTDFRSSYSSQGIPPNNNKKSPYNGVLT
HWIENNEFGEEKFLARNCNYCDLRVTRSSFICGNCQHVLHSSCARIWWEIGDECPSGCGC
NCPEMFDA

>YER155C
MKGLLWSKNRKSSTASASSSSTSTSHKTTTASTASSSSPSSSSQTIRNSTSGASPYMHSH
HHHGQGHSHHRGEDNNRDKRKSSVFPPSKQYTSTSSSQVNLGMYHSDTNTRSSRSIASTL
KDDSPSVCSEDEISNSSSQKSNAQDETPIAYKKSAHSKDSLLPSRSSSLSPPQSRCSTGT
TLEKSLNTSGISNSSGTNNNNSNNNNDNEQKQRNVIHLNSENYDTTVFKTGWVNKSHGQT
VATNYNSSMTAPSSSSSSSSQNLRNDAYSRNRESRFYGNDGSSLKNDDSSSTTATNSGND
VASARSSMAIDPQMLVPDYRLYRAQLKGCVLNLYKSGLNSNIKFFDPTLPASNSSIANEN
HQQKKQQTNNQAQAEALHQKQSFGQMGEPITLDLKYLSEVYPHPDLRQDSDGKIISGTIE
SLCHTVLFYPGPKQSDVPNEKSLSKTHRAVINLLLMFPLLDHFIKFLKVFNQFGLSFTKN
KSRLTNNSTQFYNISPAVDDSMTQRLALTAKTILDVFPGFLLDEPMLKTIISLLDTISLH
NDEISNNLKIKIANKHNELMKLTAFTRSLPMATSSTHELEIILDPSHFLSLDITTLADEV
HHINLKFDKVWAPKFDYSLLYDSKFINRRIVSLNPLVFNNDQNIHFLGRLLISHLFPTNP
EFSKKVTPKVRAELLDKWVQIGCRFEHLGDMVSWLAVATIICSIPVLRSSSWKYVPDQSL
KTIFKDWVPTIIQLERRQRTSKSTSSVFILAPPNLDDDFTRANVISYFGDLLIHADDLPS
DTKFKYLEKKINRTKNAFHKWQQRLQAIDSTRHKTNSTENVRDNDSPNNVVYQLWKFHLS
QPPLNIEGIMKLSVQHEPPIIDQKAYSTIGSQRSALVTGSYLPILFNELFPNYSLFPKNT
LVGAASDAKLPPPRSSARLSKSLSISEPIPIASNSHTMGSLTDDAMSSKNDNNKVTGVGK
IDGPVIKEMSSKQSNKQRLLKSVRDVFNIDMDVFHISDELVFKSVYDNDGKSRPASMVIE
TPKRFSQHSSMLINNPATPNQKMRDSLDTTGRLSKTLENMDFFNNIGQVSDSLKESIIRV
VLKSSSLEKIFDLLVLTSNIFSKLVDTKDLENYYYHQRQRGHSTRGLSDDNIGLLDYAFV
KLTMDNDIFTETFFNTYKSFTTTTTVLENMAKRYVGAKSCSVSISKILDRSDDSKMKINE
DTNLVSSSLYDQNFPVWDMKVTDDENINLIYMAKIQIGAAEAILHLVKNHYSDFTDDLCN
NSTLLDIIKIMEQEVSTEWPTRIANSKLQKSLPENFVIETENLLTTLTDLFHGIKSAYQK
QLYRPIGVNRTQKRITDILNSFNTFSFTDLNNIIDDPSFSDDMIRSFQKLHSTNYEDILE
WIYQLDNFISKKFNLVSKKDWIVLFQELELLSKESLVSFFNYPLHFKSSKLINPGYLQLH
EFEISNLFTWISTLILKDDNGTESLFFEKLPQSIKLLIKLHTSLTTFFVMEISNVNKSSS
ERLTTCKVILQILNYIRWKNGSLDLFDSEEDESPHAICPHIPAFIETAIAHAIISPESRN

YELSWIKASEKLSDPTKGTQNLRSISNVLEKIDDIHIKRFIEIDDVFSKNCKNLCPCPGW
FISRLLEISQFVPNMSITNSKLINFDKRRFVNNIISNVLDLIPNEREFPLDIEMSDENPS
KRTTFGRILFNNFEDVNKVYRKKTKKVSESEAISERFQEQGVFNEILVNEIEKIKREARK
LEVLLDQEKILKNSAALHQAVPKKNRKSVIISGTHSDNDHSYNINKNTGQTPSLGSVMES
NNSARNRRDSRASFSTNRSSVVSNSSHNGVSKKIGGFFRRPFSIGGFNTSSSNYSLNSIL
SQEVSSNKSILPSILPEVDSMQLHDLKPSYSLKTFEIKSIMEIINHRNIPAYYYAFKIVM
QNGHEYLIQTASSSDLTEWIKMIKASKRFSFHSKKYKGKTHNKIFGVPLEDVCERENTLI
PTIVVKLLEEIELRGLDEVGLYRIPGSIGSINALKNAFDEEGATDNSFTLEDDRWFEVNA
IAGCFKMYLRELPDSLFSHAMVNDFTDLAIKYKAHAMVNEEYKRMMNELLQKLPTCYYQT
LKRIVFHLNKVHQHVVNNKMDASNLAIVFSMSFINQEDLANSMGSRLGAVQTILQDFIKN
PNDYFKQ

>YGL105W
MSDLVTKFESLIISKYPVSFTKEQSAQAAQWESVLKSGQIQPHLDQLNLVLRDNTFIVST
LYPTSTDVHVFEVALPLIKDLVASSKDVKSTYTTYRHILRWIDYMQNLLEVSSTDKLEIN
HDLDLPHEVIEKKKKAPAGGAADAAAKADEDVSKKAKKQDHPRGKPDEETLKKLREEAKA
KKAAKKAANAKQQQEQQNKAPEKPKPSAIDFRVGFIQKAIKHPDADSLYVSTIDVGDEEG
PRTVCSGLVKHFPLDAMQERYVVVVCNLKPVNMRGIKSTAMVLCGSNDDKVEFVEPPKDS
KAGDKVFFEGFGDEAPMKQLNPKKKIWEHLQPHFTTNDGLEVIFKDEEEKDHPVRKLTNA
KGESFKVASIANAQVR

>YGL136C
MILVYNRIRSIISSSLGRIHVRYNSNSQNRWLNRQLKDPYTKEAKVQNLRSRAAFKLMQI
DDKYRLFSKNRTDQRILDLGYAPGAWSQVARQRSSPNSMILGVDILPCEPPHGVNSIQAN
ILAKRTHDLIRLFFSKHFQLNRHDDLHKDHGYFQNMLEEELTHVKDTELYREIFTSDDIY
ETPNTNSTLIEREKFPVDVIISDMYEPWPQTTGFWNNITNQAYFRMANTSGVSIRDHYQS
IDLCDAALVTAIDLLRPLGSFVCKLYTGEEENLFKKRMQAVFTNVHKFKPDASRDESKET
YYIGLKKKRNVDKLDVFSNS

>YGL154C
MVKTTEVVSEVSKVAGVRPWAGIFVVEIQEDILADEFTFEALMRTLPLASQARILNKKSF
HDRCSNLCSQLLQLFGCSIVTGLNFQELKFDKGSFGKPFLDNNRFLPFSMTIGEQYVAMF
LVKCVSTDEYQDVGIDIASPCNYGGREELELFKEVFSEREFNGLLKASDPCTIFTYLWSL
KESYTKFTGTGLNTDLSLIDFGAISFFPAEGASMCITLDEVPLIFHSQWFNNEIVTICMP
KSISDKINTNRPKLYNISLSTLIDYFIENDGL

>YGR092W
MLSKSEKNVDLLAGNMSNLSFDGHGTPGGTGLFPNQNITKRRTRPAGINDSPSPVKPSFF
PYEDTSNMDIDEVSQPDMDVSNSPKKLPPKFYERATSNKTQRVVSVCKMYFLEHYCDMFD
YVISRRQRTKQVLEYLQQQSQLPNSDQIKLNEEWSSYLQREHQVLRKRRLKPKNRDFEMI
TQVGQGGYGQVYLARKKDTKEVCALKILNKKLLFKLNETKHVLTERDILTTTRSEWLVKL
LYAFQDLQSLYLAMEFVPGGDFRTLLINTRCLKSGHARFYISEMFCAVNALHDLGYTHRD
LKPENFLIDAKGHIKLTDFGLAAGTISNERIESMKIRLEKIKDLEFPAFTEKSIEDRRKM
YNQLREKEINYANSMVGSPDYMALEVLEGKKYDFTVDYWSLGCMLFESLVGYTPFSGSST
NETYDNLRRWKQTLRRPRQSDGRAAFSDRTWDLITRLIADPINRLRSFEHVKRMSYFADI
NFSTLRSMIPPFTPQLDSETDAGYFDDFTSEADMAKYADVFKRQDKLTAMVDDSAVSSKL
VGFTFRHRNGKQGSSGILFNGLEHSDPFSTFY

>YLR264W
MDSKTPVTLAKVIKVLGRTGSRGGVTQVRVEFLEDTSRTIVRNVKGPVRENDILVLMESE
REARRLR

>YOR070C

MGVRSAAKEMHERDHNSDSSSLVTSLMKSWRISSASSSKKPSLYKMNTTESTSLPSGYAS
SADRDRRTSDGNFEAMAKQQASTRRTSNSYSPLRYVNPTLSTASNESPRPALLLRQHHQR
HHHHQQPRHSSSGSVGNNCSNSTEPNKKGDRYFKDLDEDWSAVIDDYNMPIPILTNGGFG
TPVAPTRTLSRKSTSSSINSISNMGTSAVRNSSSSFTYPQLPQLQKEKTNDSKKTQLEIE
NERDVQELNSIIQRISKFDNILKDKTIINQQDLRQISWNGIPKIHRPVVWKLLIGYLPVN
TKRQEGFLQRKRKEYRDSLKHTFSDQHSRDIPTWHQIEIDIPRTNPHIPLYQFKSVQNSL
QRILYLWAIRHPASGYVQGINDLVTPFFETFLTEYLPPSQIDDVEIKDPSTYMVDEQITD
LEADTFWCLTKLLEQITDNYIHGQPGILRQVKNLSQLVKRIDADLYNHFQNEHVEFIQFA
FRWMNCLLMREFQMGTVIRMWDTYLSETSQEVTSSYSMSSNDIKPPVTPTEPRVASFVTP
TKDFQSPTTALSNMTPNNAVEDSGKMRQSSLNEFHVFVCAAFLIKWSDQLMEMDFQETIT
FLQNPPTKDWTETDIEMLLSEAFIWQSLYKDATSHWL

>YOR275C
MSELLAIPLKRTLEVDFATELSKLIDTTSFQTASFFQSDILKVVDARNNAIAPDISIDGL
SALKEYYVILLQLEKKFPNNQIEFTWFQTLSQKSRGTSQYSLQWEKLTIIYNIGCMYSLL
ALNSNNDAAESLKTSCLYFQNAAGCFKHVLDHQKNLETIPVVDDATLNALTSLMLAQAQE
CFWFKAVQDKHKDSLIAKLSQQIVDFYCEAINDAQRGKLIRSDWINHLKAKKAYFSAVTY
YRIALSFNEKKQFGNVVKALQMGLQFINESTLSSQAKFKTVVESSLKEAQRDNEFIYLQE
VPSELPSIKPALMVKPSSSATLLPSIKKDETLFKDLIPIEVMEYCTAYNERQDEYVEQRV
TNPLASLNKLLKESLTTFQIPQGLTKVSEAELSHYQASLNNLLINNKNVQVQLDNIEQIL
NEEAFTDNQLRLKHGTLNWTLPESSTTNTAYYEKLKKLRGYLDEGSAIDKQTNELFQSID
KNLIGSEIRLPESNDPLTNKIKMIIQERNDYIDRTRRKSSEYRILPKIITSYKKNGTVDF
EPIFIGHLKYFDEDLRYVNSTKEENIKLIEEVNLSKKNNPGRSGIEPKKMVRIDPRELYI
EDLRYSFKLLDEVKENLSAGTAFYENLITSTSNLYNEVQEYDTARRAEKARLDKSLTFED
Q

>YPL106C
MSTPFGLDLGNNNSVLAVARNRGIDIVVNEVSNRSTPSVVGFGPKNRYLGETGKNKQTSN
IKNTVANLKRIIGLDYHHPDFEQESKHFTSKLVELDDKKTGAEVRFAGEKHVFSATQLAA
MFIDKVKDTVKQDTKANITDVCIAVPPWYTEEQRYNIADAARIAGLNPVRIVNDVTAAGV
SYGIFKTDLPEGEEKPRIVAFVDIGHSSYTCSIMAFKKGQLKVLGTACDKHFGGRDFDLA
ITEHFADEFKTKYKIDIRENPKAYNRILTAAEKLKKVLSANTNAPFSVESVMNDVDVSSQ
LSREELEELVKPLLERVTEPVTKALAQAKLSAEEVDFVEIIGGTTRIPTLKQSISEAFGK
PLSTTLNQDEAIAKGAAFICAIHSPTLRVRPFKFEDIHPYSVSYSWDKQVEDEDHMEVFP
AGSSFPSTKLITLNRTGDFSMAASYTDITQLPPNTPEQIANWEITGVQLPEGQDSVPVKL
KLRCDPSGLHTIEEAYTIEDIEVEEPIPLPEDAPEDAEQEFKKVTKTVKKDDLTIVAHTF
GLDAKKLNELIEKENEMLAQDKLVAETEDRKNTLEEYIYTLRGKLEEEYAPFASDAEKTK
LQGMLNKAEEWLYDEGFDSIKAKYIAKYEELASLGNIIRGRYLAKEEEKKQAIRSKQEAS
QMAAMAEKLAAQRKAEAEKKEEKKDTEGDVDMD

>YDR034C
MFESVNLDENSPEDRELAKVLSPPGSYLSPASLDSGSSFTNSGTSTSCFEPKNNLPSLSF
LNARAGSLGGIFNHKQMTSPSNSNIGGENVESTTSSNDGSNENAGHPTTSEQDQNADHPT
ISQADDNGHSSLTPNPAVTSTVTDKKGNTVKRKYSRNGCSECKRRRMKCDETKPTCWQCA
RLNRQCVYVLNPKNKKRRTSNAQRVKEFRKHSTSLDNDHNNARKRQHSSCKAEKKKKVRQ
NLSEDTTDPKPITDNGKNVPLDEIESLEIPNLDLTTTMNGYDVNLLMQNLNDMVNMKLHD
SYLLNEELKGLDLPDLDIPELLPASNVNSSVPISFLVNNVITFNTKLSSFKLGGIHDKYL
KIFYYDCLDSIAPFFQNQGNPLRDILLSFAKNEAYLLSSILATGASIAYRKSNNLEDERN
YCAYLSHCLSLLGEQFKNESNVLNRIEPIILTVIMLAWDCIYSMNSQWRSHLKGVTDLFK
KINAGNSSKVLNVAKCWFKVMETFASISTVFGGSLIDNNDLDAIFDPYDYQYVDSLKFLN
IMTPLNEFNLLRGHKEDFDLVIKEVFKSLNTIRSTEKNYFSKEEGLFTKKLDYLLLSSQT
SSEKSKDQISYFNTQKILVEIDKQLDYEFIDKSGIIPSDNQSHPRISNIHDNAIDMVTLK
NGEEVAISWYDISHQTQVLSFLLIVLLKLLGMPKESSTIQQVVKKIMSFFKFLDSDSPPQ

NSRTCYSNFAVLIAGLNAMDEETRAIVKRYYKINGGKFQKLTEHNLNRLEKVWYGKNQNY
RLEEQDVLTW

>YJL124C
MSANSKDRNQSNQDAKRQQQNFPKKISEGEADLYLDQYNFTTTAAIVSSVDRKIFVLLRD
GRMLFGVLRTFDQYANLILQDCVERIYFSEENKYAEEDRGIFMIRGENVVMLGEVDIDKE
DQPLEAMERIPFKEAWLTKQKNDEKRFKEETHKGKKMARHGIVYDFHKSDMY

>YGL045W
MSLLRLWNKESRAPSKIKSHGIVGSYGNSMLAHNNVKQFRIDIDEPHRVWKPNESITGEA
VIDIKRDITNVAIKLSLVCEVRVKTGNSPTSKNKRIEKTLEKSTFLYGQDYVKTAFSAKE
KKPHVDKTTILNGLSKGEHRFPFRIRIPRGRGMLSSIKFERGSITYFLSCTLESLNNING
LKKPEARCEREFAVIVPLDVSRLPKPKTKTVVLQSASMVQNKKNKSTEDESSSYTQLTQK
STTSNSSSSSVNSKTSPLPNKTVTISVDIPQAGFMIGEIIPIDVKIDHYKPFYAPAGLTT
TLVRICRVGGAGKDDPMETFRKDICQSISPIYINPETLQFQSRVYLKVPLDAFSTLTTVG
KFFSFQYYIEVMVNLSKKNVVYTESNRIIGTPIGEQNGLGVENNINRIQRKMLRMVNPET
LENDSEGYESSIFFKDMVNVEKLKRLRNVTGMSIETVIGTTRSEQQQSDASIPSQSSITA
PQNSPSNLRDWLAPLNAYDSDDVPVPKYSPNDKVSVPSEDKQELEQKRLQQLESDPPPCD
DY
