# Supplementary material for: Stress Granule-Defective Mutants Deregulate Stress Responsive Transcripts
Source: PLoS Genet. 2014 Nov 6;10(11):e1004763. doi: 10.1371/journal.pgen.1004763 (PMC4222700; doi:10.1371/journal.pgen.1004763)
Supplement: Table S3 — Statistical significance P values for changes in heat-induced mutation rates in wt and mutant strains. The P values corresponding to the data in Fig. 4A are listed. (DOCX) [file pgen.1004763.s012.docx]

**Supplementary Table S3**

Statistical P values for the observed differences in heat-induced mutation rate shown in Figure 4 A

| P-value | treated vs. untreated | wt vs. mutants (under heat stress) |
| --- | --- | --- |
| BY 4741 | 0.843 | 1 |
| *top3Δ* | 1.11 × 10^-5^ | 2.42 × 10^-5^ |
| *ypr172wΔ* | 0.0132 | 0.106 |
| *mft1Δ* | 0.000396 | 0.184 |
| *tif4632Δ* | 0.00621 | 0.335 |
| *vta1Δ* | 0.113 | 0.0433 |
| *set3Δ* | 0.00610 | 0.00167 |
| *fit2Δ* | 8.54 × 10^-5^ | 0.0183 |
| *mrpl10Δ* | 8.23 × 10^-6^ | 0.242 |
| *ski3Δ* | 0.0286 | 0.0217 |
| *ptk2Δ* | 0.000814 | 0.106 |
| *pcp1Δ* | 0.149 | 0.0942 |
| *gtr2Δ* | 0.158 | 0.680 |
| *gtr1Δ* | 5.10 × 10^-7^ | 0.000159 |
