# Supplementary material for: Stress Granule-Defective Mutants Deregulate Stress Responsive Transcripts
Source: PLoS Genet. 2014 Nov 6;10(11):e1004763. doi: 10.1371/journal.pgen.1004763 (PMC4222700; doi:10.1371/journal.pgen.1004763)
Supplement: Text S1 — Supporting information reference. (DOCX) [file pgen.1004763.s013.docx]

Text S1. Supporting Information Reference.

Swisher KD, Parker R (2010) Localization to, and effects of Pbp1, Pbp4, Lsm12, Dhh1, and Pab1 on stress granules in *Saccharomyces cerevisiae*. PLoS One 5: e10006.
